# Reductive Cyclopentamerization of Carbon Monoxide to the Elusive Croconate Radical Trianion

**DOI:** 10.1021/jacs.6c02757

**Published:** 2026-03-19

**Authors:** Arpan Mondal, Thayalan Rajeshkumar, Alexander Steiner, Jinkui Tang, Laurent Maron, Richard A. Layfield

**Affiliations:** † Department of Chemistry, School of Life Sciences, 1948 University of Sussex, Brighton BN1 9RH, U.K.; ‡ Laboratoire de Physique et Chimie des Nano-objets, 27091 Université de Toulouse, Institut National des Sciences Appliquées, CNRS, 31077 Cedex 4 Toulouse, France; § Department of Chemistry, University of Liverpool, Crown St, Liverpool L69 7ZD, U.K.; ∥ State Key Laboratory of Rare Earth Resource Utilization, Changchun Institute of Applied Chemistry, Chinese Academy of Sciences, Changchun 130022, P.R. China

## Abstract

Coupling reactions of carbon monoxide represent an ideal synthetic route to cyclic oxocarbons. Whereas oxocarbon anions based on rings of three, four and six carbon atoms have been synthesized from CO coupling, cyclic five-membered croconate anions have evaded capture by this method. Here, we show that CO coupling initiated by rare-earth dinitrogen complexes in concert with molybdenum hexacarbonyl yields rare-earth complexes of the exotic croconate radical trianion, [C_5_O_5_]^3–^, a species only observed previously under cryogenic irradiation. Contrasting reactions of the rare-earth dinitrogen complexes with CO alone produce ketene-carboxylate anions, highlighting the crucial templating role of molybdenum in forming croconate. Spectroscopic and computational analyses establish the radical nature of the croconate anion and reveal a heterobimetallic reaction pathway for its assembly with multielectron transfer. This discovery surmounts a long-standing challenge in oxocarbon chemistry, illustrating how strongly reducing rare-earth compounds and mixed-metal cooperativity can enable the isolation of sought-after organic radicals.

## Graphical abstract

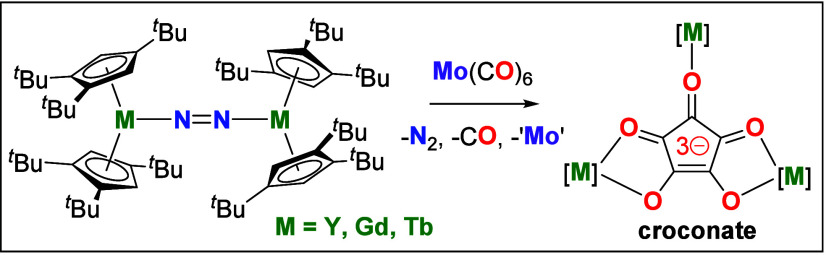

Oxocarbon materials, especially croconate anions [C_5_O_5_]^*x*−^, currently attract interest owing to their applications as high-capacity electrodes in alkali-ion batteries.^1–3^ Carbon monoxide is an attractive precursor to oxocarbons, whereas the classical organic synthesis of such compounds is challenging, often requiring multistep preparation of functionalized precursors.^4–7^ Traditional oxocarbon synthesis also suffers from low yields and purification issues, typically yielding only dianionic oxocarbons rather than the more highly charged species relevant to energy storage.^8–11^ In contrast, a variety of linear, branched or cyclic oxocarbon anions with the formula [C_*n*_O_*n*_]^*x*−^ (*x* up to 6) can be synthesized directly from CO in reactions promoted by strongly reducing f-element^12–16^ and main-group compounds,^17–25^ typically under mild conditions. Milestones include cyclization of CO to the deltate and squarate dianions promoted by uranium­(III) organometallics,^26,27^ and formation of the rhodizonate hexa-anion driven by a magnesium­(I) reagent^28^ (Scheme 1a). Despite the remarkable advances, five-membered croconate anions have resisted all attempts to synthesize them using CO coupling strategies.

**Scheme 1:**
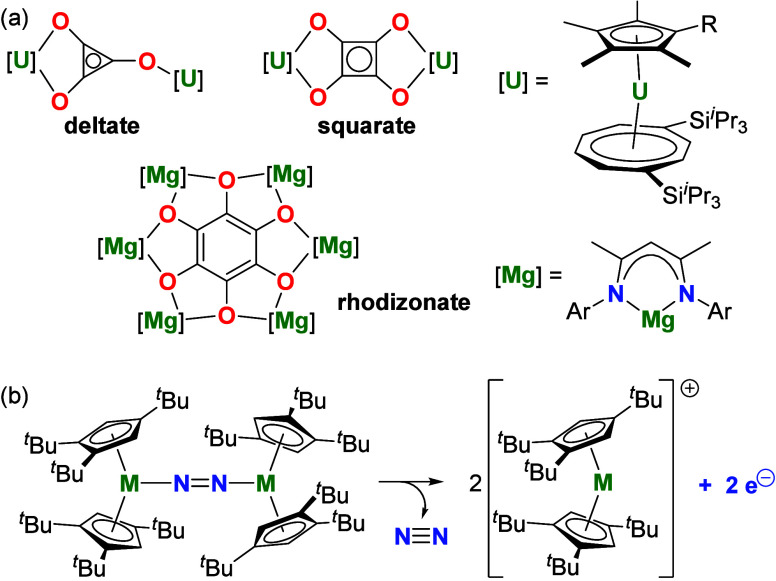
^a^

a (a) Examples of cyclic oxocarbon anions formed by CO homologation. (b) Masked divalent reactivity of rare-earth dinitrogen complexes.

Compounds of rare-earth elements in the oxidation state +2 are powerful reducing agents with potential use in CO coupling.^29,30^ However, the divalent oxidation state is unstable for most of these elements, which restricts synthetic applications.^31^ An alternative strategy is to use the idea of “masked” divalent oxidation states, in which rare-earth complexes containing the metal in the stable oxidation state +3 store electrons on a redox-active ligand before delivering them to a substrate.^32,33^ This approach is exemplified by rare-earth complexes of the reduced dinitrogen ligand [N_2_]^2–^,^34–39^ which can initiate multielectron reduction processes with elimination of N_2_ (Scheme 1b).^40,41^

The capacity of rare-earth dinitrogen complexes to assemble extended oxocarbon frameworks via CO homologation remains to be fully exploited. Here, we show that the rare-earth metallocenes [{(Cp^ttt^)_2_M}_2_(μ-1,2-N_2_)] (M = Y, Gd, Tb; Cp^ttt^ = 1,2,4-tri­(*tert*-butyl)­cyclopentadienyl) react with molybdenum hexacarbonyl as a CO surrogate to produce isolable complexes of the croconate radical trianion, [C_5_O_5_]^3–^ (Scheme 2). The crystals obtained from the reactions were analyzed by X-ray diffraction, revealing that the croconate anions are bound to three rare-earth metallocenes in the complexes [{(Cp^ttt^)_2_M}_3_(C_5_O_5_)] (**1**_**M**_).

**Scheme 2:**
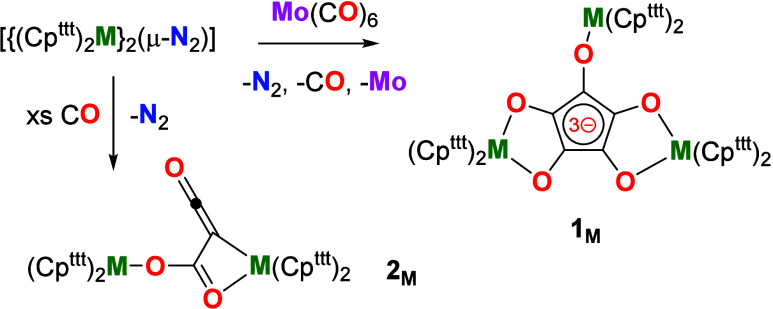
Reactions of Rare-Earth Dinitrogen Complexes with Mo­(CO)_6_ and CO to give **1**_**M**_ and **2**_**M**_, Respectively (M = Y, Tb, Gd)

The molecular structures of **1**_**M**_ feature a planar croconate ligand bound to two metal centers in a bidentate manner and to the third metal in a monodentate manner (Figure 1a, Figures S1, S2, Tables S1, S2). Relative to their dinitrogen precursor complexes,^40,41^ the M–(Cp^ttt^)_cent_ distances in **1**_**M**_ are slightly longer and the (Cp^ttt^)-M-(Cp^ttt^) angles are slightly narrower (‘cent’ denotes the center of the cyclopentadienyl ring). For example, in **1**_**Gd**_, the M–(Cp^ttt^)_cent_ distance of 2.4606(15) Å is longer by 0.025 Å and the (Cp^ttt^)-M-(Cp^ttt^) angle is 4.3° narrower than in [{(Cp^ttt^)_2_Gd}­(μ-1,2-N_2_)], probably for steric reasons. The C–C bond distances in the croconate ligands of **1**_**M**_ lie in narrow ranges (Table S2), for example 1.418(8)-1.439(8) Å in **1**_**Gd**_, consistent with delocalization of π-electron density around the five-membered ring.

**Figure 1:**
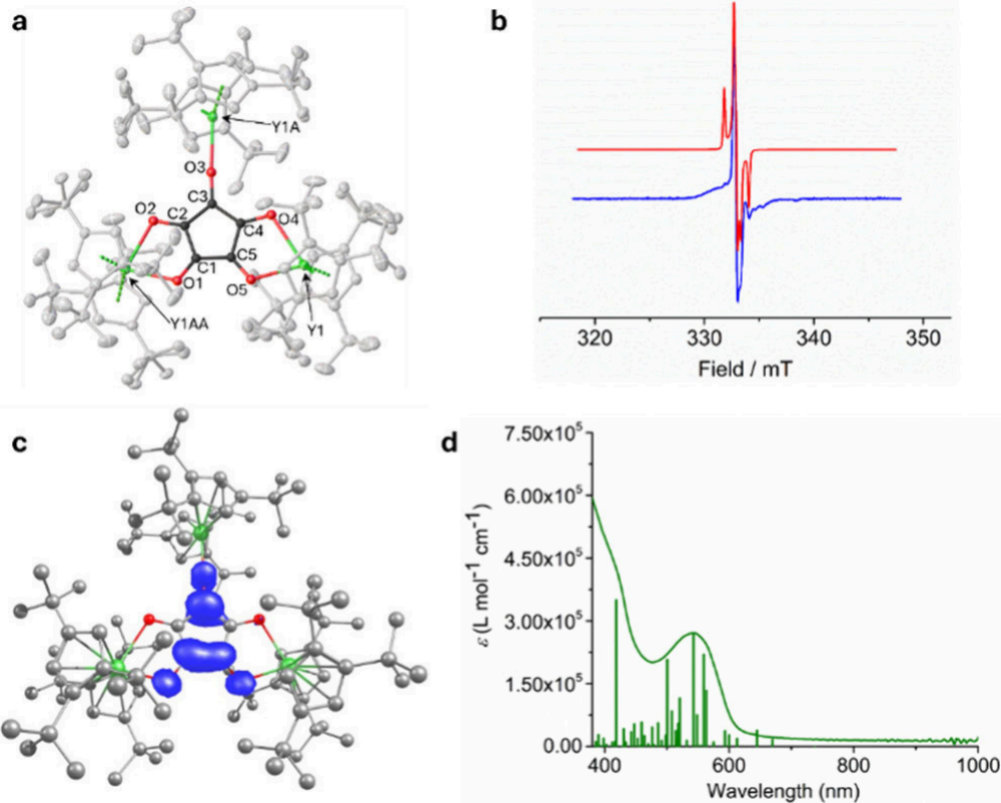
(a) Molecular structure of [{(Cp^ttt^)_2_Y}_3_(C_5_O_5_)] (**1**_**Y**_). (b) X-Band EPR spectrum of **1**_**Y**_ in toluene at 100 K (blue) with simulation (red) using *g* = 2.01778, 2.01442, and 2.00861, and *A*(^89^Y) = 27.6954, 2.05741, and 21.0984 MHz. (c) DFT-calculated spin density of **1**_**Y**_ at the TPSSh/def2-TZVP/def2-SVP level of theory (isovalue = 0.002). (d) UV–visible spectrum of **1**_**Y**_ in toluene, with electronic transitions calculated at the TPSSh/def-TZVP level of theory shown as vertical lines.

The FTIR and Raman spectra of **1**_**M**_ display features resembling those reported for croconic acid and alkali metal croconate salts (Figures S5, S7–S9).^42,43^ For **1**_**Gd**_, three strong Raman bands corresponding to C–O stretching occur at 1546, 1619, and 1684 cm^–1^, and two weaker bands located at 1108 and 1216 cm^–1^ are associated with C–C stretching. A strong band at 678 cm^–1^ is assigned to croconate out-of-plane bending. The crystallographic and Raman data therefore support the assignment of **1**_**M**_ as a croconate trianion [C_5_O_5_]^3–^ bound to three [(Cp^ttt^)_2_M]^+^ units.

An implication of the croconate ligand in **1**_**M**_ forming as a trianion is that it should be an *S* = 1/2 radical. It is therefore noteworthy that essentially all known croconate salts and coordination complexes are based on the dianionic form of the oxocarbon.^44,45^ The croconate radical trianion has previously only been observed upon X-ray irradiation of [CsNa­(C_5_O_5_)] at 77 K,^46^ emphasizing its exotic and highly reactive nature. The only other proposed croconate radical is the putative mono- anion, which has been proposed to form during the charging/discharging cycles of sodium-ion batteries based on croconate cathodes.^1,3^ Focusing on **1**_**Y**_, the X-band EPR spectrum at 100 K shows a resonance characteristic of an organic radical (Figure 1b). Consistent with this, a density functional theory (DFT) calculation of the spin density in **1**_**Y**_ shows that the unpaired electron is distributed across three carbon and three oxygen atoms of the [C_5_O_5_]^3–^ anion (Figure 1c, Table S5). A comparable spin distribution was also calculated for the croconate ligand in **1**_**Gd**_ along with seven unpaired electrons on the metal centers, consistent with three Gd^3+^ ions bridged by [C_5_O_5_]^3–^ (Figure S22). The magnetic susceptibility of **1**_**Gd**_ also agrees with the assignment of formal charges to the metal centers and the ligand. Thus, the molar magnetic susceptibility (χ_M_) of **1**_**Gd**_ gives a χ_M_ *T* value of 23.54 cm^3^ K mol^–1^ at 300 K, close to the theoretical value of 24.02 cm^3^ K mol^–1^ for three Gd^3+^ ions and one unpaired electron (Figures S14, S15).^47^ Magnetic susceptibility measurements on **1**_**Tb**_ are likewise consistent with three Tb^3+^ ions and an organic radical (Figures S16, S17).

DFT calculations of the frontier molecular orbitals (MOs) for **1**_**Y**_ and **1**_**Gd**_ show that the highest-occupied MO (HOMO) and the lowest-unoccupied MO (LUMO) are predominantly croconate-based (Figures S23, S24). To validate the calculated electronic structure of **1**_**Y**_ and **1**_**Gd**_ against experiment, time-dependent DFT (TD-DFT) calculations of the electronic excitations were performed and compared to their UV–visible spectra. The three spectra are similar (Figure 1d, Figure S12), with an intense absorption below 400 nm and a broad absorption centered on λ_max_ = 524, 544, and 557 nm for **1**_**Y**_, **1**_**Gd**_ and **1**_**Tb**_, respectively. The calculated transitions for **1**_**Y**_ (Figure 1d, Table S6) and **1**_**Gd**_ (Figure S26, Table S7) reproduce the experimental spectra well, showing that the low-energy absorption corresponds to transitions from a Cp^ttt^-based bonding MO and a metal d-orbital to a croconate π* orbital (Figures S25, S27).

To identify the influence of mixed-metal cooperativity in the formation of **1**_**M**_, reactions of the gadolinium and terbium dinitrogen complexes with excess CO were performed. The products were analyzed by crystallography and found to be dimetallic complexes with the metals bridged by dianionic ketene-carboxylate ligands, i.e., [{(Cp^ttt^)_2_M}_2_(C_3_O_3_)] (**2**_**M**_, M = Gd, Tb) (Scheme 2) (Figures S3, S4, S6, S10, S11, Tables S3, S4). Reactions of the dinitrogen complexes with Mo­(CO)_6_ were also conducted under an atmosphere of CO and produced only the croconate complexes, with no ketene-carboxylate products detected. This observation supports a molybdenum-templated mechanism rather than one governed by free CO in solution, signposting a crucial role for molybdenum in promoting CO cyclization. Furthermore, the reactions of [{(Cp^ttt^)_2_M}_2_(μ-1,2-N_2_)] (M = Y, Gd, Tb) with Cr­(CO)_6_ and W­(CO)_6_ were attempted to establish if the reactivity is general for group 6 metals. Intractable oils were obtained from the reactions of Cr­(CO)_6_ with the dinitrogen complexes, and the reactions with W­(CO)_6_ did not yield any crystalline material. These observations also suggest that Mo­(CO)_6_ is necessary to form the croconate ligands observed in **1**_**M**_.

The magnetic properties of **2**_**M**_ are also consistent with the presence of two weakly coupled M^3+^ ions (Figures S18–S21), and their UV–visible spectra are featureless above 400 nm (Figure S13), underscoring the distinct electronic structure of the croconate ligand in **1**_**M**_. The product of reacting CO gas with the trivalent dinitrogen complexes is the same as that reported for the reaction of the thulium­(II) metallocene (Cp^ttt^)_2_Tm with CO,^29^ corroborating the notion that the dinitrogen complexes can indeed be regarded as masked divalent reagents. Consequently, this synthetic approach enables divalent reactivity for rare-earth elements that do not normally adopt this oxidation state.^48,49^

To gain insight into the templating role of Mo­(CO)_6_, a mechanism for the formation of **1**_**Y**_ was explored computationally at the B3PW91 level of theory, including dispersion corrections and solvent effects (Scheme 3). The initial step involves displacement of N_2_ by Mo­(CO)_6_ to form the trimetallic intermediate **Int1**, with two isocarbonyl linkages to one yttrium center and a third isocarbonyl linkage to the other yttrium. The first C–C bond forms between two CO ligands via **TS1** to give **Int2** and is calculated to have a small Δ*G* of 1.9 kcal mol^–1^. A second C–C coupling proceeds via **TS2** to form **Int3** and is thermodynamically slightly unfavorable. This is followed by a strongly favorable step involving reduction by a third electron and incorporation of the third yttrium center, forming **Int4** (Δ*G* = – 45.7 kcal mol^–1^). Formation of the third C–C bond occurs through **TS3** to give **Int5**, which rearranges to position the {Mo–C–O–Y} connectivity close to one terminus of the growing carbon chain in **Int6**. An alternative pathway to the third C–C bond that does not involve the additional reduction was identified but was calculated to be unfavorable (Figure S28). The fourth C–C coupling then proceeds via **TS4** to give **Int7**, corresponding to a six-membered {MoC_5_} metallacycle. Closure of the five-membered ring occurs via **TS5** to afford the croconate complex **Int8** in an energetically favorable step (Δ*G* = – 32.1 kcal mol^–1^), featuring side-on coordination of the croconate ligand to molybdenum and stabilization from a C–H interaction involving a Cp^ttt^ ligand.

**Scheme 3:**
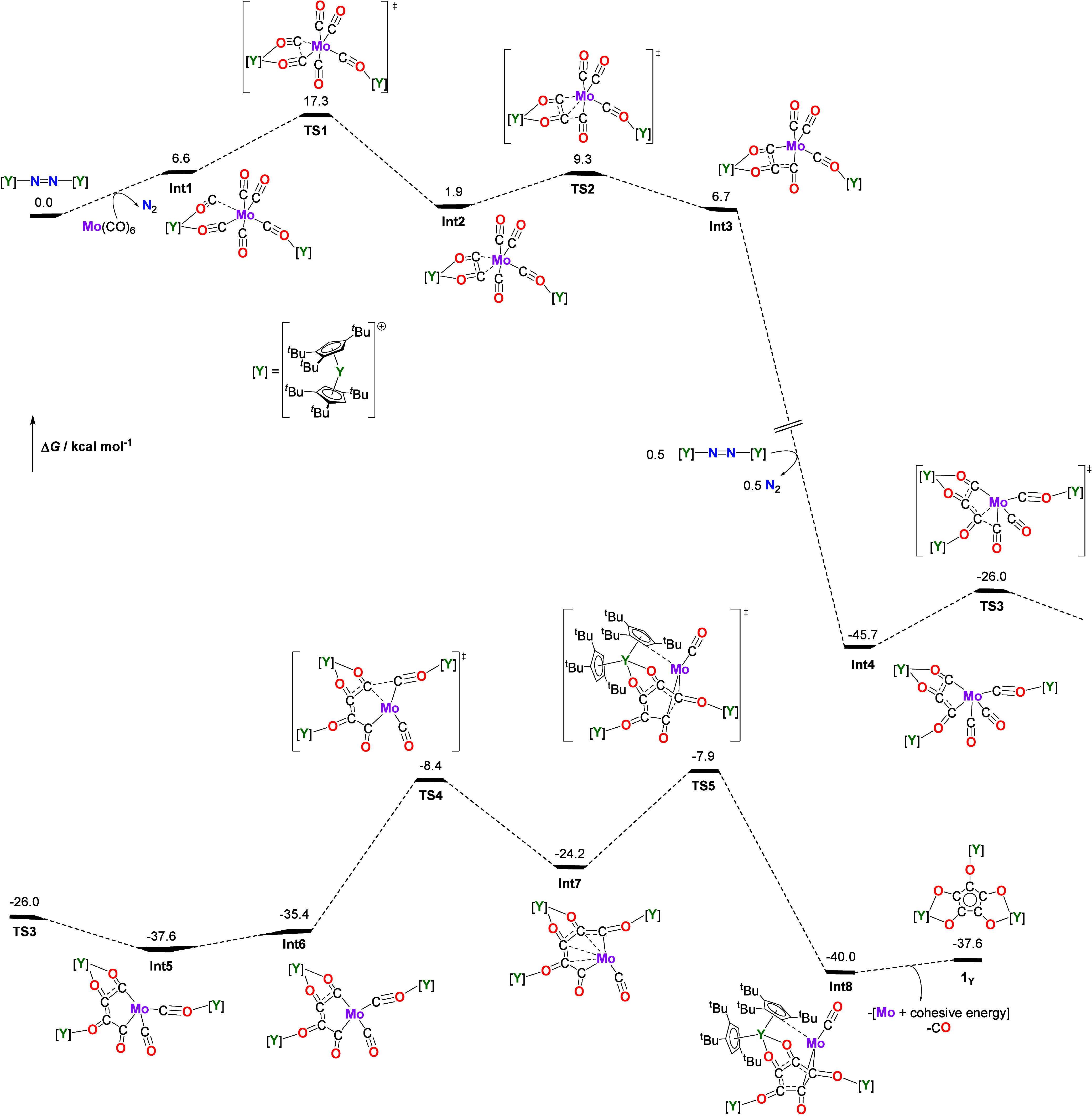
DFT-Calculated Mechanism for the Formation of **1**_**Y**_ ^a^

a Numbers represent free energy changes in kcal mol^–1^ relative to [{(Cp^ttt^)_2_M}_2_(μ-1,2-N_2_)]. Calculations were performed at the B3PW91 level of theory with dispersion corrections. Solvent (toluene) effects were accounted for with single-point energy calculations.

The relatively large Δ*G* increase accompanying formation of **TS5** from **Int4** (37.8 kcal mol^–1^) is consistent with the slow reaction at room temperature and the low isolated yield of **1**_**Y**_. This large barrier and the low yield may reflect the bulky nature of the molecule and is reminiscent of a comparable barrier reported for the aluminum­(I)-initiated homologation of CO to a [C_4_O_4_]^4–^ chain.^25^ The final step leading to **1**_**Y**_ is calculated to occur with Δ*G* = 2.4 kcal mol^–1^ with respect to **Int8**. However, the presumed formation of molybdenum metal provides a substantial driving force from the cohesive energy, while release of CO provides a favorable entropic term. Overall, these effects render the transformation slightly favorable in terms of free energy.

Hybrid DFT methods such as B3PW91 are known to exhibit systematic uncertainties for multimetallic, open-shell transition-metal systems.^50–52^ Consequently, the computed free energies should be regarded semiquantitatively, with greater emphasis on the relative ordering of intermediates and transition states than on the precise magnitude of individual barriers. The calculated profile remains energetically reasonable, and the highest barrier is consistent with the experimentally observed slow reaction. The overall pathway shows a feasible multistep C–C coupling pathway and the proposed templating role of Mo­(CO)_6_. The reaction is driven largely by enthalpy via the bond forming steps, with entropy penalties incurred owing to the reorganization required to form the intermediates and product. Without molybdenum, the local concentration of CO in the coordination sphere of yttrium is insufficient to support cyclization, leading to ketene-carboxylate formation, consistent with the mechanism reported for [{(Cp^ttt^)_2_Tm}_2_(C_3_O_3_)].^29^

In conclusion, compounds **1**_**M**_ containing the croconate radical trianion have been isolated directly from CO homologation. Overcoming the limits of traditional multistep synthesis, our approach exploits the powerful reducing capacity of masked divalent rare-earth dinitrogen complexes in synergy with molybdenum to assemble a croconate framework from the simplest oxocarbon building block. Experimental and computational studies confirm stabilization of the nonaromatic anion [C_5_O_5_]^3–^, hitherto observed only as a fleeting excited state. The contrasting reactions of the dinitrogen complexes with Mo­(CO)_6_ versus CO alone highlight the key role of heterobimetallic cooperativity in directing extended CO homologation, potentially signposting a general strategy for stabilizing otherwise inaccessible oxocarbon frameworks.

## Supplementary Material



## Data Availability

Additional research data supporting this publication are available as supplementary information at DOI: 10.25377/sussex.30139255.

## Notes

Synthesis, spectroscopic characterization, crystallography details, magnetic measurements, computational details (PDF)
