# Supplementary material for: Reductive Cyclopentamerization of Carbon Monoxide to the Elusive Croconate Radical Trianion
Source: J Am Chem Soc. 2026 Mar 19;148(12):12507–12. doi: 10.1021/jacs.6c02757 (PMC13047684; doi:10.1021/jacs.6c02757)
Supplement: Supplementary file 1 [file ja6c02757_si_001.pdf]

Reductive Cyclopentamerization of Carbon Monoxide to the Elusive Croconate Radical Tri-Anion

Arpan Mondal,[1] Thayalan Rajeshkumar,[2] Alexander Steiner,[3] Jinkui Tang,[4] Laurent Maron,[2]* Richard A. Layfield[1]*

[1] Department of Chemistry, School of Life Sciences, University of Sussex, Brighton, BN1 9RH, U.K.
[2] Laboratoire de Physique et Chimie des Nano-objets, Université de Toulouse, Institut National des Sciences Appliquées, CNRS, 31077 Cedex 4 Toulouse, France.
[3] Department of Chemistry, University of Liverpool, Crown St, Liverpool L69 7ZD, U.K.
[4] State Key Laboratory of Rare Earth Resource Utilization, Changchun Institute of Applied Chemistry, Chinese Academy of Sciences, Changchun, 130022, P.R. China.

*E-mail: laurent.maron@irsamc.ups-tlse.fr, r.layfield@sussex.ac.uk

Contents

General Considerations page 2
Synthesis Methods page 2-3
X-ray Crystallography page 4-11
FTIR Spectra page 12-13
Raman Spectra page 14-18
UV-visible Spectra page 19-20
Magnetic Property Measurements page 21-29
DFT Calculations page 30-40
Optimized Geometries page 41-139
References page 140-141

General Considerations

All reactions were carried out under rigorous anaerobic, anhydrous conditions using argon or nitrogen atmospheres and Schlenk and glove-box techniques. All solvents were refluxed over an appropriate drying agent for a minimum of three days (molten potassium for toluene and Na/K alloy for hexane) and then distilled and degassed via a minimum of three freeze-pump-thaw cycles. Solvents were then stored in ampoules over potassium mirrors. Literature procedures were used to synthesise [$\{(Cp^{ttt})_2M\}_2(\mu\text{-}1,2\text{-}N_2)$] (M = Y, Gd, Tb).[1,2] $Mo(CO)_6$ was purchased from Sigma-Aldrich and used without further purification.

FTIR spectra were recorded on a Bruker Alpha FTIR spectrometer with a platinum-diamond ATR module, housed in a glovebox.

Raman spectra were measured using a Renishaw inVia confocal Raman microscope using an excitation laser wavelength of 532 nm and 1800 mm^{-1} grating. Laser power was kept below 0.5 mW to reduce heating effects. Measurements used a 20× magnification objective, resulting in a laser spot size of about 1.60 mm. Spectra were baseline corrected, and cosmic rays were removed using WiRE software. For the baseline, the software automatically uses an 'intelligent fitting', which excludes regions with peaks and fits the rest of the spectrum using a polynomial expression.

X-Band EPR spectra were recorded at the PEPR Facility, Imperial College, in CW mode on a Bruker EMX spectrometer equipped with a Bruker ER049X SuperX microwave bridge, a Bruker ER4122SHQE resonator, and an Oxford Instruments ITC503 temperature controller.

Magnetic measurements were performed on a Quantum Design MPMS3 SQUID magnetometer equipped with a 7 T magnet. Samples were prepared by gently crushing the crystalline materials before transferring them to a 7 mm NMR tube and covering them in eicosane. Then the tubes were flame sealed under a static vacuum. The eicosane was melted in a water bath at 40 °C to prevent crystallite torquing. Diamagnetic corrections were performed using Pascal's constants.[4]

Synthesis Methods

Synthesis of [$\{(Cp^{ttt})_2Y\}_3(C_5O_5)$] ($\mathbf{1_Y}$)

Toluene (6 mL) was added to a mixture of [$\{(Cp^{ttt})_2Y\}_2(\mu\text{-}1,2\text{-}N_2)$] (100 mg, 0.087 mmol) and $Mo(CO)_6$ (11.5 mg, 0.043 mmol) at –78°C, and the suspension was slowly warmed to room temperature and stirred for 24 hours, producing a deep red-purple solution. Following filtration, the solvent was removed under reduced pressure to yield a solid residue, which was redissolved in a minimum amount of toluene and allowed to evaporate slowly for crystallization. Crystals suitable for single crystal X-ray diffraction were obtained after six days from the concentrated toluene solution (15 mg, 15% based on yttrium). **Elemental analysis** (%), found (calculated) for $C_{107}H_{174}O_5Y_3$: C 70.89 (71.11); H 9.68 (9.70). **FTIR** ($\tilde{\upsilon}/cm^{-1}$): 3000-2800 (m, br.), 1685 (w, br.), 1556 (str., s), 1457 (str., s), 1419 (str., s), 1392 (str., s), 1355 (str., s), 1239 (str., s), 820 (str., s), 683 (str., s). **Raman** ($\tilde{\upsilon}/cm^{-1}$): 1688, 1622, 1550, 1222, 1116, 681. Compound $\mathbf{1_Y}$ has very low solubility in most common solvents, e.g., THF, precluding analysis by NMR spectroscopy.

Synthesis of [{(Cp^{ttt})$_2$Gd}$_3$(C_5O_5)] (1_{Gd})

Toluene (6 mL) was added to a mixture of [{(Cp^{ttt})$_2$Gd}$_2$(μ-1,2-N_2)] (100 mg, 0.078 mmol) and $Mo(CO)_6$ (10.3 mg, 0.039 mmol), and the suspension was stirred for 24 hours, producing a red-purple solution. Following filtration, the solvent was then removed under reduced pressure to give a solid residue, which was redissolved in a minimum amount of cold hexane and kept at –35 °C for 3 days, leading to the formation of red-purple microcrystals of **1_{Gd}** (28 mg, 27% based on gadolinium). Crystals suitable for single crystal X-ray diffraction were grown by the slow evaporation of a concentrated hexane solution. **Elemental analysis** (%), found (calculated) for $C_{107}H_{174}O_5Gd_3$: C 63.20 (63.87); H 8.68 (8.72). **FTIR** ($\tilde{\upsilon}$/cm^{-1}): 3000-2800 (m, br.), 1686 (w, br.), 1552 (str., s), 1455 (str., s), 1418 (str., s), 1389 (str., s), 1356 (str., s), 1241 (str., s), 818 (str., s), 682 (str., s). **Raman** ($\tilde{\upsilon}$/cm^{-1}): 1684, 1619, 1546, 1216, 1108, 678.

Synthesis of [{(Cp^{ttt})$_2$Tb}$_3$(C_5O_5)] (1_{Tb})

Compound **1_{Tb}** was synthesised using the procedure described for **1_{Gd}**, using [{(Cp^{ttt})$_2$Tb}$_2$(μ-1,2-N_2)] (100 mg, 0.078 mmol) and $Mo(CO)_6$ (10.3 mg, 0.052 mmol), and isolated as deep red-purple microcrystals (30 mg, 28% based on terbium). Crystals suitable for single crystal X-ray diffraction were grown by slow evaporation of a concentrated hexane solution. **Elemental analysis** (%), found (calculated) for $C_{107}H_{174}O_5Tb_3$: C 63.30 (63.71); H 8.61 (8.69). **FTIR** ($\tilde{\upsilon}$ /cm^{-1}): 3000-2800 (m, br.), 1689 (w, br.), 1559 (str., s), 1456 (str., s), 1421 (str., s), 1392 (str., s), 1356 (str., s), 1240 (str., s), 820 (str., s), 681 (str., s). **Raman** ($\tilde{\upsilon}$/cm^{-1}): 1686, 1620, 1549, 1218, 1112, 679.

Synthesis of [{(Cp^{ttt})$_2$Gd}$_2$(C_3O_3)] (2_{Gd})

Hexane (6 mL) was purged with excess CO and added to [{(Cp^{ttt})$_2$Gd}$_2$(μ-1,2-N_2)] (100 mg, 0.078 mmol) at –78°C. The reaction was slowly warmed up to room temperature and stirred for 24 hours, producing a light red-purple solution. Following filtration, the solvent was removed under reduced pressure to give a solid residue, which was washed three times with a minimum amount of cold hexane, leading to the formation of a light, yellow-pink solid of **2_{Gd}** (28 mg, 27% based on gadolinium). Crystals suitable for single crystal X-ray diffraction were grown by the slow evaporation of a concentrated hexane solution. **Elemental analysis** (%), found (calculated) for $C_{71}H_{116}O_3Gd_2$: C 63.87 (64.01); H 8.69 (8.78). **FTIR** ($\tilde{\upsilon}$/cm^{-1}): 3000-2800 (br., m), 2242 (s, str.), 2063 (s, str.), 1486-1434 (br., m), 1358 (s, str.) 1266-1238 (m, str.), 1001 (s, str.), 824-813 (d, str.), 683-678 (d, str.). **Raman** ($\tilde{\upsilon}$/cm^{-1}): 1458, 1390, 1250, 783.

Synthesis of [{(Cp^{ttt})$_2$Tb}$_2$(C_3O_3)] (2_{Tb})

Compound **2_{Tb}** was synthesised using the procedure described for **2_{Gd}**, using [{(Cp^{ttt})$_2$Tb}$_2$(μ-1,2-N_2)] (100 mg, 0.078 mmol), and isolated as light pink colour solid (30 mg, 28% based on terbium). Crystals suitable for single crystal X-ray diffraction were grown by slow evaporation of a concentrated hexane solution. **Elemental analysis** (%), found (calculated) for $C_{71}H_{116}O_3Tb_2$: C 63.39 (63.85); H 8.67 (8.76). **FTIR** ($\tilde{\upsilon}$/cm^{-1}): 3000-2800 (br., m), 2242 (s, str.), 2064 (s, str.), 1486-1434 (br., m), 1358 (s, str.), 1259-1235 (d, str.), 1003 (s, str.), 825-815 (d, str.), 683-677 (d, str.). **Raman** ($\tilde{\upsilon}$/cm^{-1}): 1447, 1387, 1250, 785.

X-Ray Crystallography

Single-crystal X-ray diffraction measurements were carried out on a Bruker D8 Venture Metaljet diffractometer using Ga-K$_\alpha$ radiation (λ = 1.34139 Å). Structures were solved in Olex2 with SHELXT using intrinsic phasing and were refined with SHELXL using least squares minimisation.[3-5] Anisotropic thermal parameters were used for non-hydrogen atoms and isotropic parameters for hydrogen atoms. Hydrogen atoms on carbons were added geometrically and refined using a riding model. The croconate ions in crystals of $\mathbf{1_Y}$, $\mathbf{1_{Gd}}$ and $\mathbf{1_{Tb}}$ are disordered since the complexes are centred on Wyckoff sites 2a of symmetry 32. It was possible to refine the croconate positions with moderately strong same-distance restraints rather than as rigid groups. Croconate atoms were refined isotropically to minimise correlations. Bond lengths and angles of the croconate ion should be treated with some caution in view of the disorder.

Table S1. Crystal data and structure refinement parameters for $\mathbf{1_Y}$, $\mathbf{1_{Gd}}$ and $\mathbf{1_{Tb}}$.

	$\mathbf{1_Y}$	$\mathbf{1_{Gd}}$	$\mathbf{1_{Tb}}$
CCDC ref. code	2479491	2429528	2429527
Empirical formula	$C_{107}H_{174}O_5Y_3$	$C_{107}H_{174}O_5Gd_3$	$C_{107}H_{174}O_5Tb_3$
Formula weight	1807.18	2012.20	2017.21
Crystal system	trigonal	trigonal	trigonal
Space group	$P\bar{3}c1$	$P\bar{3}c1$	$P\bar{3}c1$
a/Å	16.4260(4)	16.4412(3)	16.4193(5)
b/Å	16.4260(4)	16.4412(3)	16.4193(5)
c/Å	21.8446(8)	21.9536(7)	21.9257(9)
α/°	90	90	90
β/°	90	90	90
γ/°	120	120	120
V/Å^3	5104.3(3)	5139.3(2)	5119.1(4)
Z	2	2	2
ρ_{calc}/g cm^{-3}	1.176	1.300	1.309
F (000)	1946.0	2096.0	2102.0
Reflections collected	40417	134878	108720
Independent reflections	3111	4464	3513
R_{int} (%)	0.090	0.0724	0.0724
GOF on F^2	1.046	0.988	1.008
R_1^{a}	0.0492	0.0324	0.0309
wR_2^{b}	0.1341	0.0953	0.0864

^{a} $R_1[I>2\sigma(I)] = \sum||F_o|-|F_c||/\sum|F_o|$; ^{b} wR_2[all data] $= [\sum\{w(F_o^2-F_c^2)^2\}/\sum\{w(F_o^2)^2\}]^{1/2}$

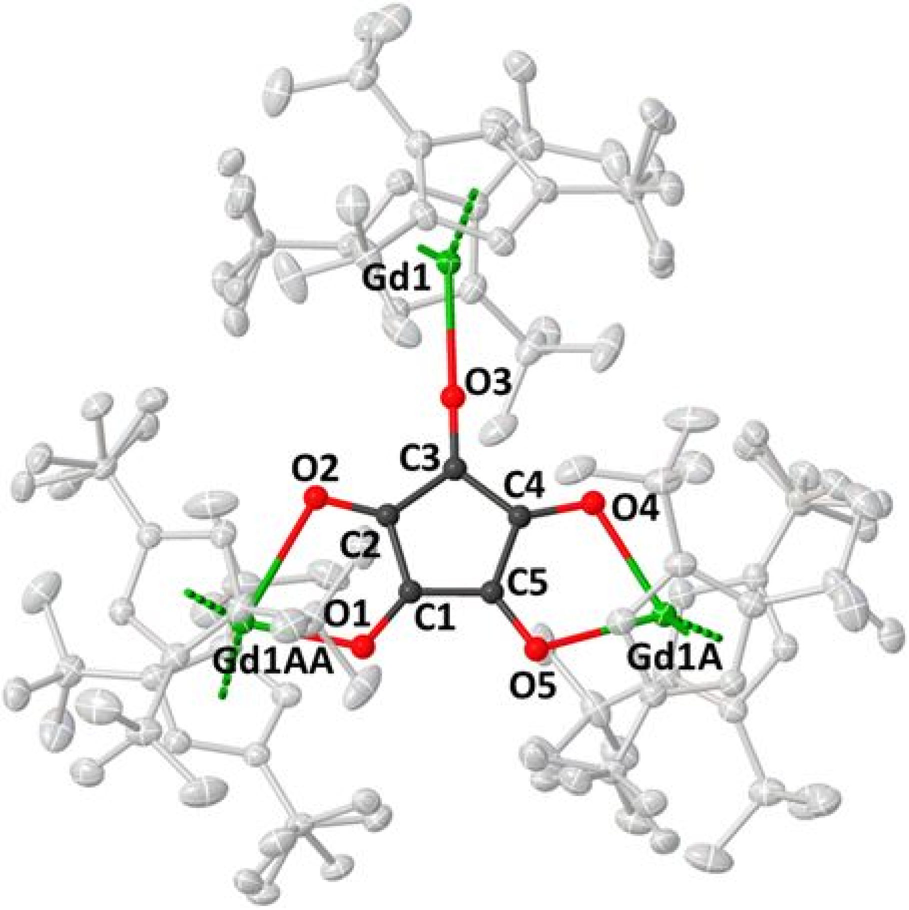

Figure S1. Thermal ellipsoid representations (30% probability) of the molecular structure of $\mathbf{1}_{\mathbf{Gd}}$. Hydrogen atoms are omitted for clarity.

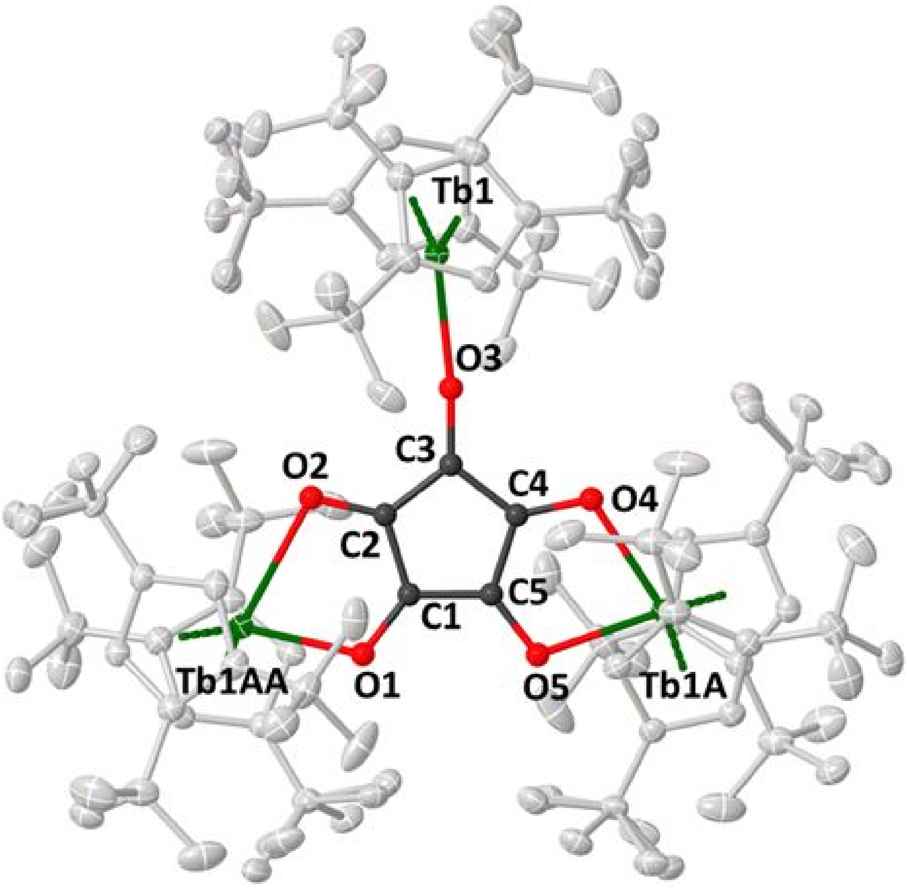

Figure S2. Thermal ellipsoid representations (30% probability) of the molecular structure of $\mathbf{1}_{\mathbf{Tb}}$. Hydrogen atoms are omitted for clarity.

Table S2. Selected bond lengths (Å) and angles (°) for $\mathbf{1_Y}$, $\mathbf{1_{Gd}}$ and $\mathbf{1_{Tb}}$.

	$\mathbf{1_Y}$	$\mathbf{1_{Gd}}$	$\mathbf{1_{Tb}}$
M–C	Y1-C10: 2.766(4)<br>Y1-C20: 2.736(4)<br>Y1-C30: 2.639(4)<br>Y1-C40: 2.685(4)<br>Y1-C50: 2.684(5)	Gd1-C10: 2.812(3)<br>Gd1-C20: 2.768(3)<br>Gd1-C30: 2.679(3)<br>Gd1-C40: 2.722(3)<br>Gd1-C50: 2.728(4)	Tb1-C10: 2.795(4)<br>Tb1-C20: 2.752(4)<br>Tb1-C30: 2.658(3)<br>Tb1-C40: 2.703(4)<br>Tb1-C50: 2.709(5)
M–$(Cp^{ttt})_{cent}$	2.4167(19)	2.4606(15)	2.4413(18)
M–O	2.22(2)<br>2.347(19)<br>2.341(11)<br>2.49(2)<br>2.52(2)	2.161(9)<br>2.336(10)<br>2.323(9)<br>2.395(10)<br>2.563(12)	2.171(10)<br>2.313(12)<br>2.314(11)<br>2.355(12)<br>2.556(13)
C–C (C_5O_5 ligand)	C1-C2: 1.451(14)<br>C2-C3: 1.44(2)<br>C3-C4: 1.44(2)<br>C4-C5: 1.451(17)<br>C5-C1: 1.43(3)	C1-C2: 1.435(8)<br>C2-C3: 1.439(8)<br>C3-C4: 1.429(8)<br>C4-C5: 1.439(8)<br>C5-C1: 1.418(8)	C1-C2: 1.442(10)<br>C2-C3: 1.437(10)<br>C3-C4: 1.430(10)<br>C4-C5: 1.442(10)<br>C5-C1: 1.427(10)
C–O	C1-O1: 1.299(19)<br>C2-O2: 1.30(3)<br>C3-O3: 1.307(15)<br>C4-O4: 1.30(3)<br>C5-O5: 1.299(19)	C1-O1: 1.291(8)<br>C2-O2: 1.294(8)<br>C3-O3: 1.299(8)<br>C4-O4: 1.302(8)<br>C5-O5: 1.295(8)	C1-O1: 1.309(10)<br>C2-O2: 1.312(9)<br>C3-O3: 1.325(9)<br>C4-O4: 1.322(9)<br>C5-O5: 1.313(9)
Cp-M-Cp	139.69(11)	140.34(8)	140.31(10)

Table S3. Crystal data and structure refinement parameters for $\mathbf{2_{Gd}}$ and $\mathbf{2_{Tb}}$.

	$\mathbf{2_{Gd}}$	$\mathbf{2_{Tb}}$
CCDC ref. code	2429526	2429525
Empirical formula	$C_{71}Gd_2O_3H_{116}$	$C_{71}O_3Tb_2H_{116}$
Formula weight	1332.13	1335.47
Crystal system	orthorhombic	orthorhombic
Space group	$Pca2_1$	$Pca2_1$
a/Å	21.3009(9)	21.2717(8)
b/Å	10.8658(4)	10.8618(4)
c/Å	28.9346(12)	28.8710(11)
α/°	90	90
β/°	90	90
γ/°	90	90
V/Å^3	6697.0(5)	6670.6(4)
Z	4	4
ρ_{calc}/g cm^{-3}	1.321	1.330
F (000)	2776.0	2784.0
Reflections collected	84723	58154
Independent reflections	11556	11408
R_{int} (%)	0.1077	0.0538
GOF on F^2	1.058	1.082
$R_1{}^a$	0.0491	0.0446
$wR_2{}^b$	0.1190	0.1184

^{a} $R_1[I>2\sigma(I)] = \sum||F_o|-|F_c||/\sum|F_o|$; ^{b} wR_2[all data] $= [\sum\{w(F_o^2-F_c^2)^2\}/\sum\{w(F_o^2)^2\}]^{1/2}$

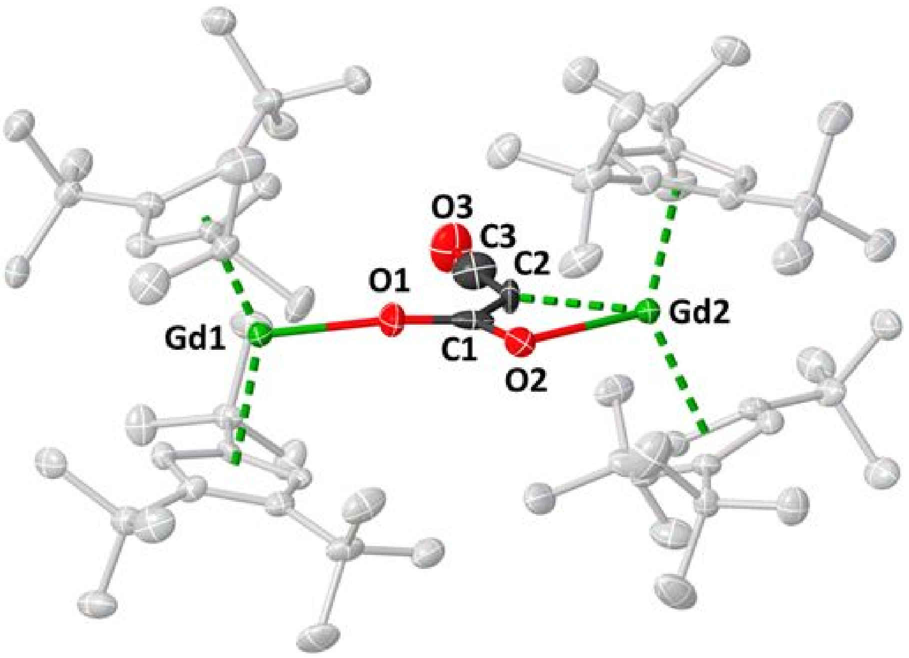

Figure S3. Thermal ellipsoid representations (30% probability) of the molecular structure of $\mathbf{2_{Gd}}$. Hydrogen atoms are omitted for clarity.

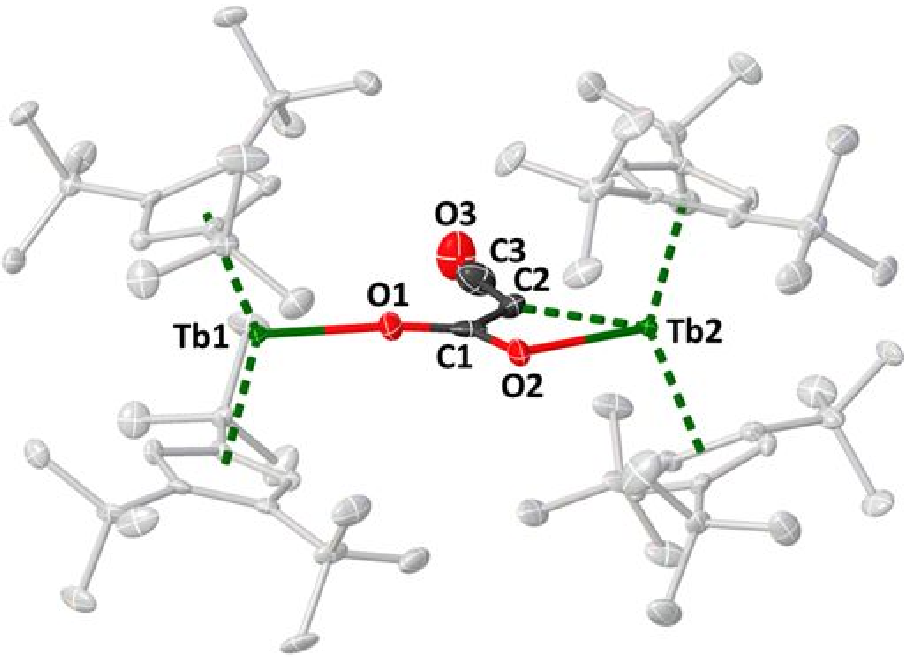

Figure S4. Thermal ellipsoid representations (30% probability) of the molecular structure of $\mathbf{2_{Tb}}$. Hydrogen atoms are omitted for clarity.

Table S4. Selected bond lengths (Å) and angles (°) for $\mathbf{2_{Gd}}$ and $\mathbf{2_{Tb}}$.

	$\mathbf{2_{Gd}}$	$\mathbf{2_{Tb}}$
M–C (Cp^{ttt} ligand)	Gd1-C4: 2.694(11)<br>Gd1-C5: 2.817(12)<br>Gd1-C6: 2.814(12)<br>Gd1-C7: 2.685(11)<br>Gd1-C8: 2.681(11)<br>Gd1-C21: 2.825(12)<br>Gd1-C22: 2.825(13)<br>Gd1-C23: 2.683(12)<br>Gd1-C24: 2.708(11)<br>Gd1-C25: 2.712(10)<br>Gd2-C38: 2.663(10)<br>Gd2-C39: 2.776(10)<br>Gd2-C40: 2.757(12)<br>Gd2-C41: 2.665(11)<br>Gd2-C42: 2.659(10)<br>Gd2-C55: 2.675(12)<br>Gd2-C56: 2.685(12)<br>Gd2-C57: 2.754(12)<br>Gd2-C58: 2.781(11)<br>Gd2-C59: 2.669(11)	Tb1-C4: 2.654(10)<br>Tb1-C5: 2.775(11)<br>Tb1-C6: 2.758(9)<br>Tb1-C7: 2.683(9)<br>Tb1-C8: 2.679(10)<br>Tb1-C21: 2.776(9)<br>Tb1-C22: 2.670(10)<br>Tb1-C23: 2.651(8)<br>Tb1-C24: 2.634(9)<br>Tb1-C25: 2.762(8)<br>Tb2-C38: 2.799(10)<br>Tb2-C39: 2.674(10)<br>Tb2-C40: 2.681(10)<br>Tb2-C41: 2.676(10)<br>Tb2-C42: 2.814(10)<br>Tb2-C55: 2.769(10)<br>Tb2-C56: 2.652(9)<br>Tb2-C57: 2.667(10)<br>Tb2-C58: 2.686(10)<br>Tb2-C59: 2.795(9)
M–Cp^{ttt}_{cent}	2.459(5), 2.475(5)<br>2.430(5), 2.421(5)	2.425(4), 2.412(4)<br>2.435(5), 2.448(5)
M–O	2.153(9), 2.352(12)	2.129(9), 2.336(9)
M–C2 (C_3O_3 ligand)	2.514(12)	2.491(15)
C–O	C1-O1: 1.309(19)<br>C1-O2: 1.263(16)<br>C3-O3: 1.20(2)	C1-O1: 1.251(17)<br>C1-O2: 1.292(16)<br>C3-O3: 1.17(2)
C–C (C_3O_3 ligand)	C1-C2: 1.417(19)<br>C2-C3: 1.30(2)	C1-C2: 1.43(2)<br>C2-C3: 1.34(2)
Cp-M-Cp	143.01(19), 145.50(19)	144.09(14), 143.14(16)

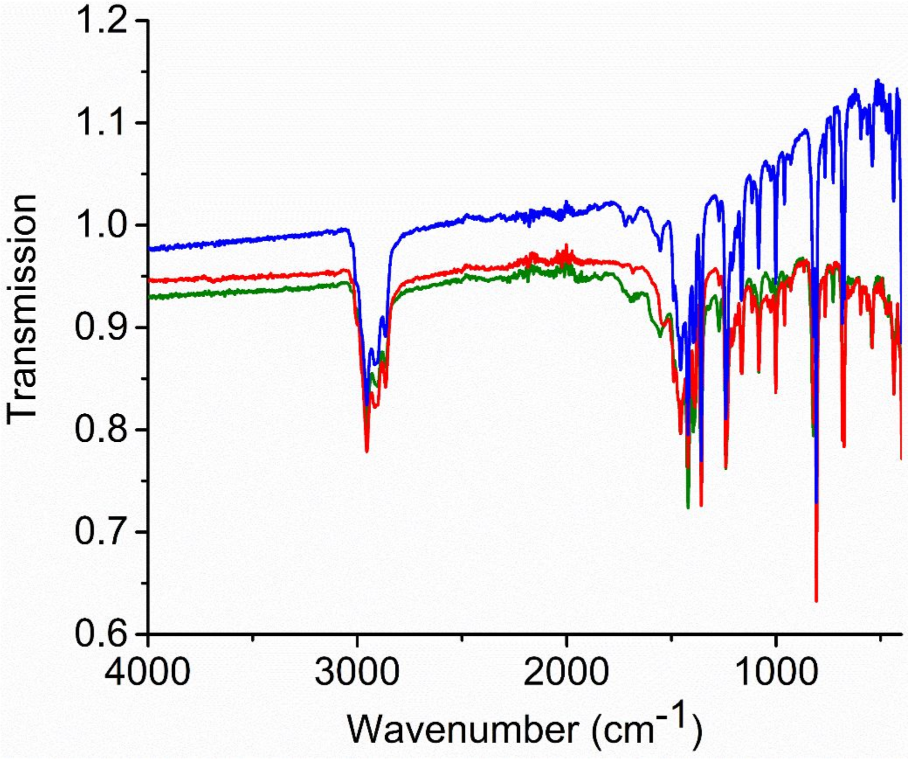

Figure S5. FTIR spectra of $\mathbf{1_Y}$ (green), $\mathbf{1_{Gd}}$ (red) and $\mathbf{1_{Tb}}$ (blue).

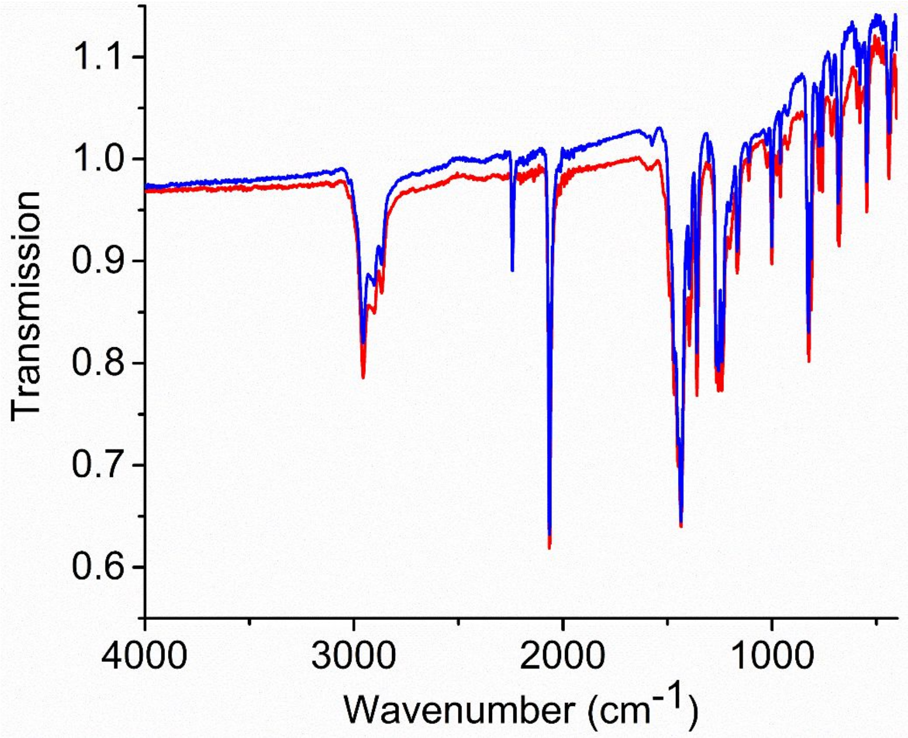

Figure S6. FTIR spectra of $\mathbf{2}_{\mathbf{Gd}}$ (red) and $\mathbf{2}_{\mathbf{Tb}}$ (blue).

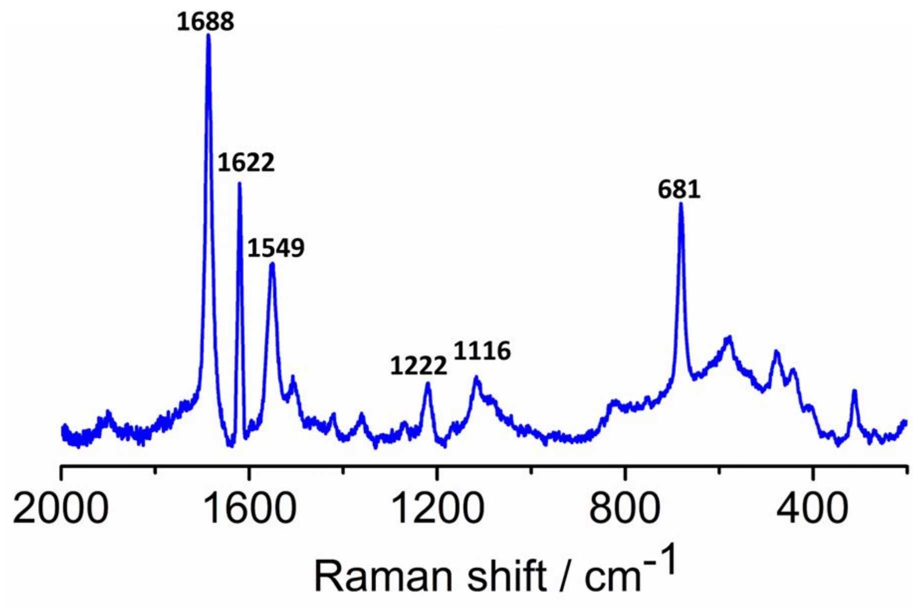

Figure S7. Normalized Raman spectrum of $\mathbf{1_Y}$ with baseline correction.

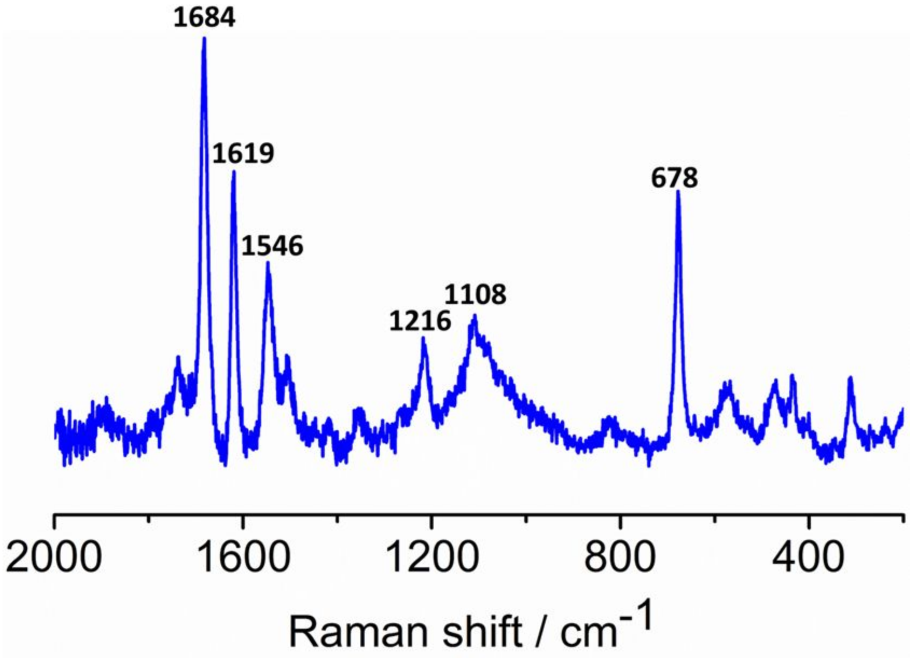

Figure S8. Normalized Raman spectrum of $\mathbf{1_{Gd}}$ with baseline correction.

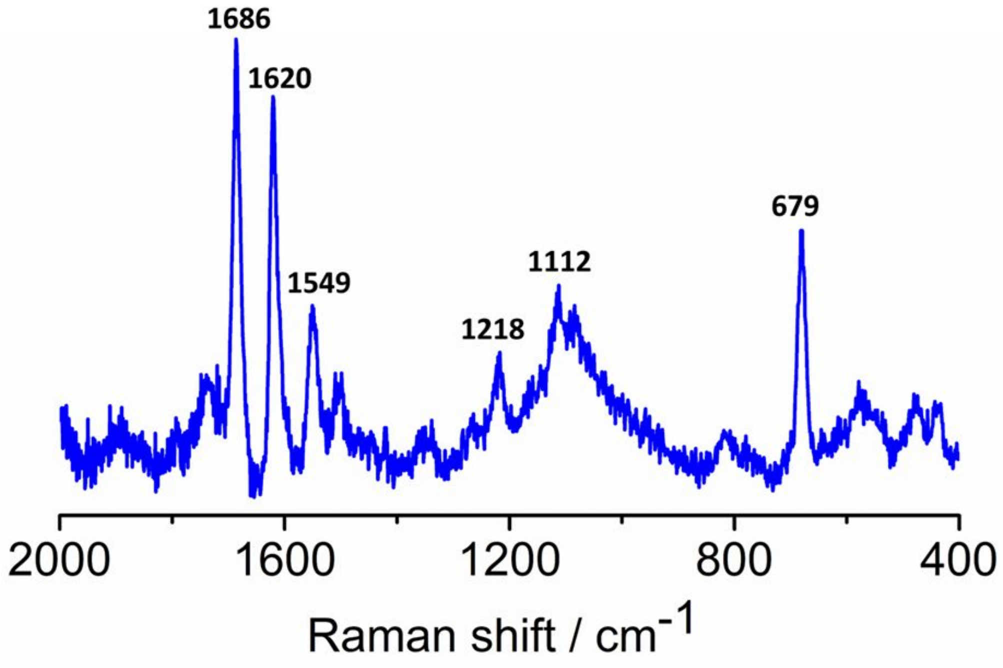

Figure S9. Normalized Raman spectrum of $\mathbf{1_{Tb}}$ with baseline correction.

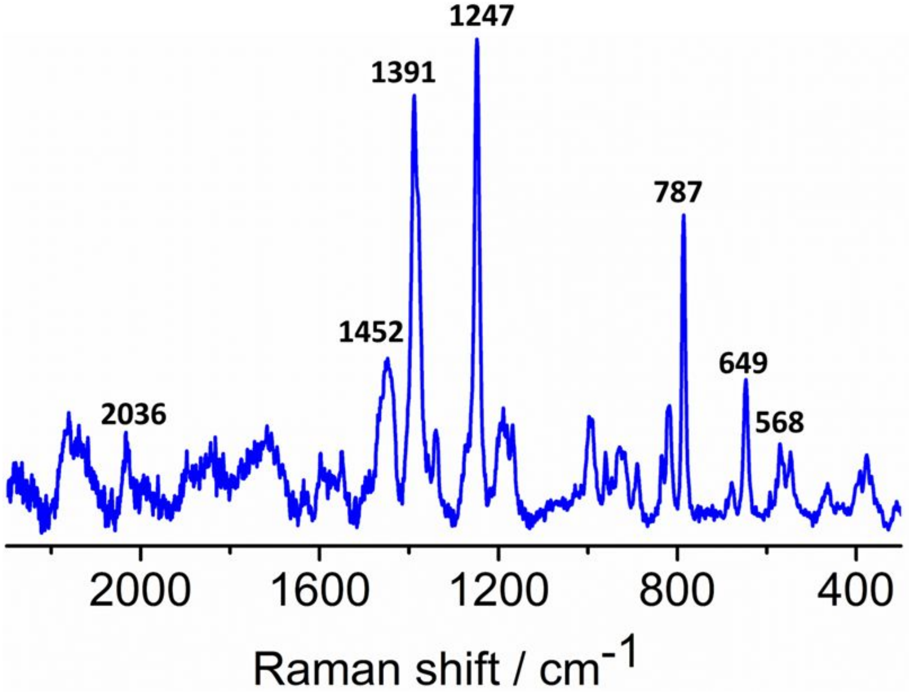

Figure S10. Normalized Raman spectrum of $\mathbf{2_{Gd}}$ with baseline correction.

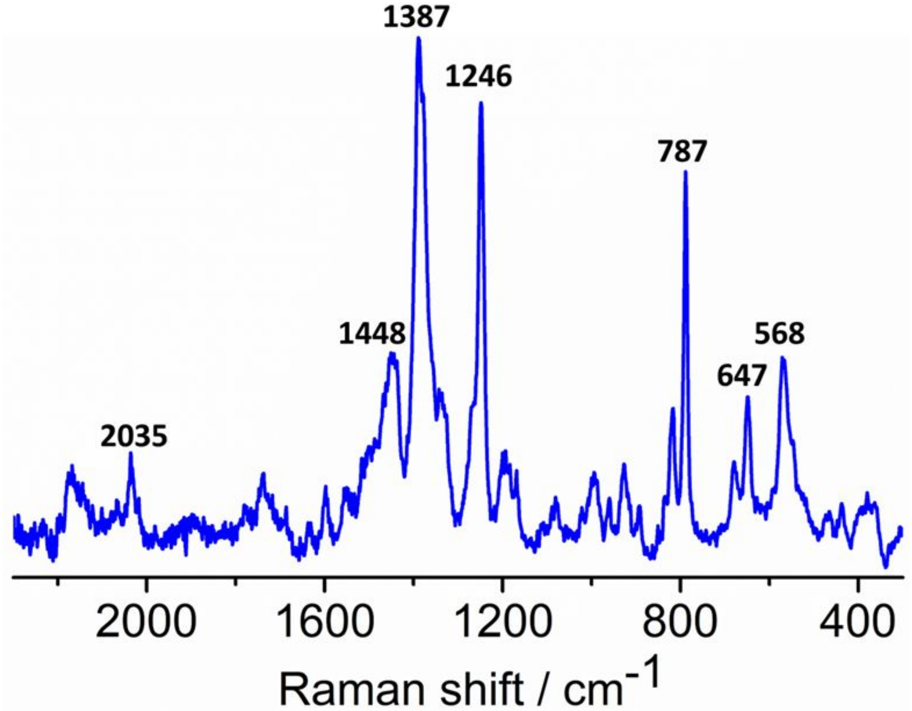

Figure S11. Normalized Raman spectrum of $\mathbf{2_{Tb}}$ with baseline correction.

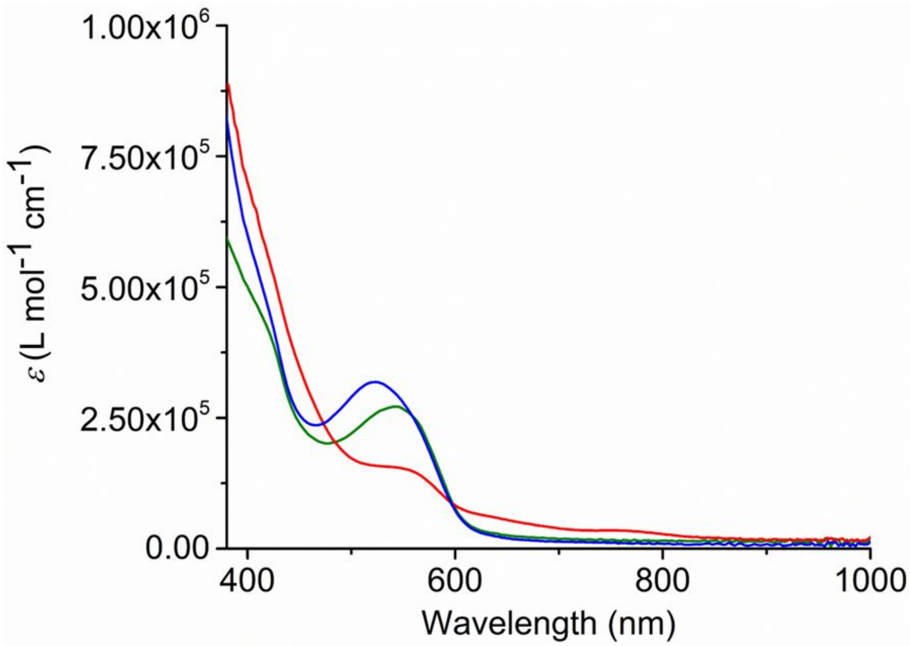

Figure S12. UV/vis spectra of $\mathbf{1_Y}$ (green, λ_{max} = 544 nm) $\mathbf{1_{Gd}}$ (red, λ_{max} = 557 nm) and $\mathbf{1_{Tb}}$ (blue, λ_{max} = 524 nm) in toluene.

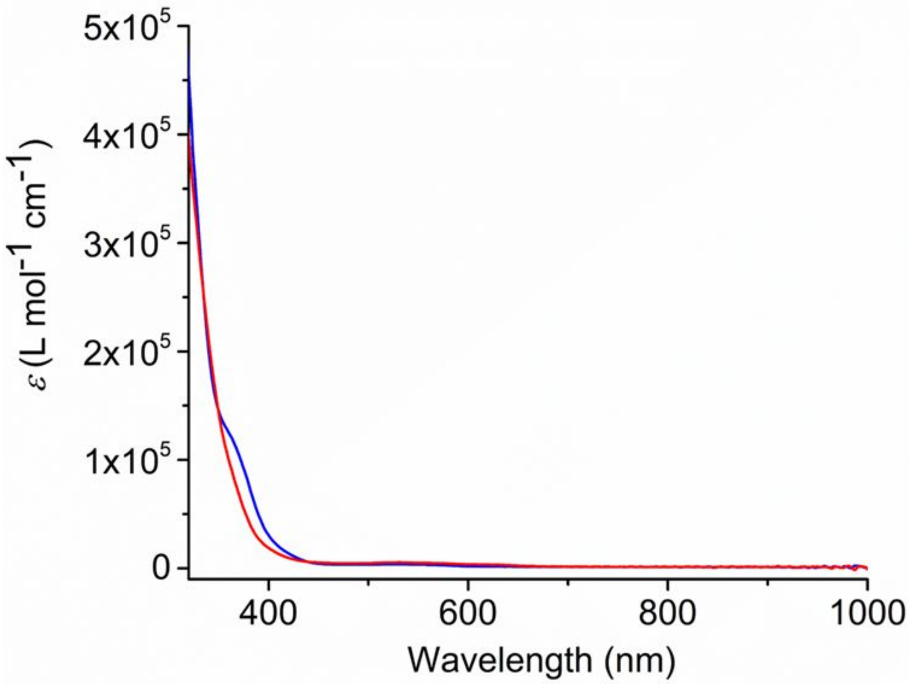

Figure S13. UV/vis spectra of $\mathbf{2_{Gd}}$ (blue) and $\mathbf{2_{Tb}}$ (red) in toluene.

Magnetic Property Measurements

Magnetic measurements were performed using a Quantum Design MPMS3 SQUID magnetometer equipped with a 7 T magnet. Samples were prepared by gently crushing the crystalline materials before transferring them to a 7 mm NMR tube and covering them in eicosane. Then the tubes were flame sealed under a static vacuum. The eicosane was melted in a water bath at 40 °C to prevent crystallite torquing. Diamagnetic corrections were performed using Pascal's constants.[6] Data fits for the gadolinium complexes were achieved using the PHI software.[7]

The following spin Hamiltonian was used to fit the magnetic data for $\mathbf{1_{Gd}}$:

$$\hat{H} = -2J_{\mathrm{Gd-Rad}}\left(\hat{S}_{\mathrm{Gd1}} \cdot \hat{S}_{\mathrm{rad}} + \hat{S}_{\mathrm{Gd2}} \cdot \hat{S}_{\mathrm{rad}} + \hat{S}_{\mathrm{Gd3}} \cdot \hat{S}_{\mathrm{rad}}\right)$$

$$+g\mu_{\mathrm{B}}\left(\hat{S}_{\mathrm{Gd1}} + \hat{S}_{\mathrm{Gd2}} + \hat{S}_{\mathrm{Gd3}} + \hat{S}_{\mathrm{rad}}\right) \cdot B$$

$$+ zJ'(\hat{S}_{\mathrm{Gd1,z}} + \hat{S}_{\mathrm{Gd2,z}} + \hat{S}_{\mathrm{Gd3,z}} + \hat{S}_{\mathrm{rad,z}}) \quad \text{(eqn. S1)}$$

The following spin Hamiltonian was used to fit the magnetic data for $\mathbf{2_{Gd}}$:

$$\hat{H} = -2J_{\mathrm{GdGd}}\hat{S}_{\mathrm{Gd1}} \cdot \hat{S}_{\mathrm{Gd2}} + g\mu_{\mathrm{B}}\left(\hat{S}_{Gd1} + \hat{S}_{Gd2}\right) \cdot B + zJ'(\hat{S}_{Gd1,z} + \hat{S}_{Gd2,z}) \quad \text{(eqn. S2)}$$

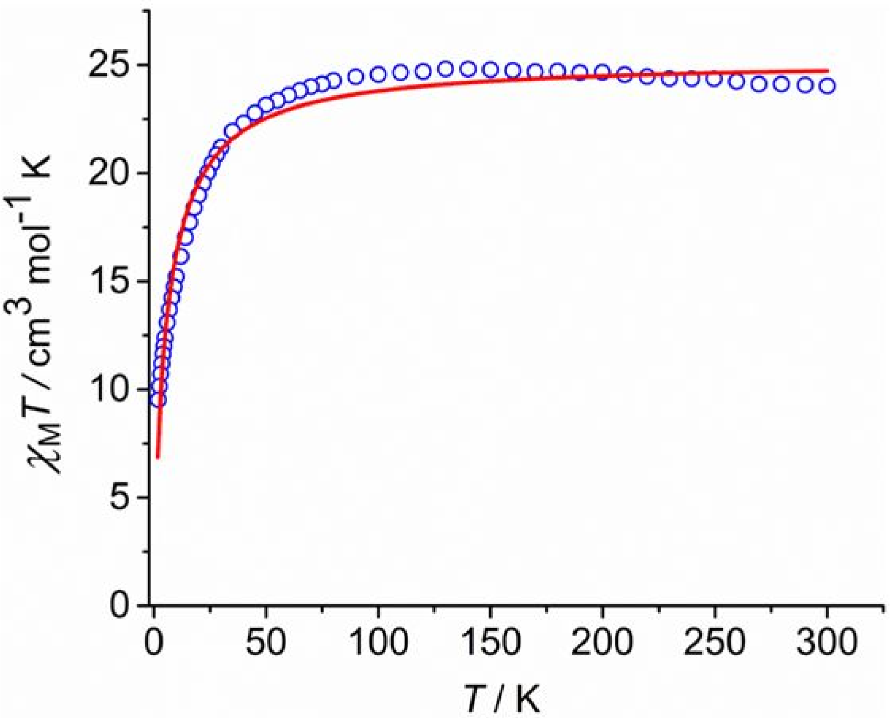

Figure S14. $\chi_M T(T)$ data for $\mathbf{1_{Gd}}$ in an applied field of 1000 Oe. $\chi_M T$ is 23.54 cm^3 K mol^{-1} at 300 K and 9.46 cm^3 K mol^{-1} at 2 K. The red line represents the best fit using the isotropic spin Hamiltonian shown in equation S1 with g = 2.00, $J_{\text{Gd-rad}}$ = –0.52 cm^{-1} and zJ' = –0.01 cm^{-1}.

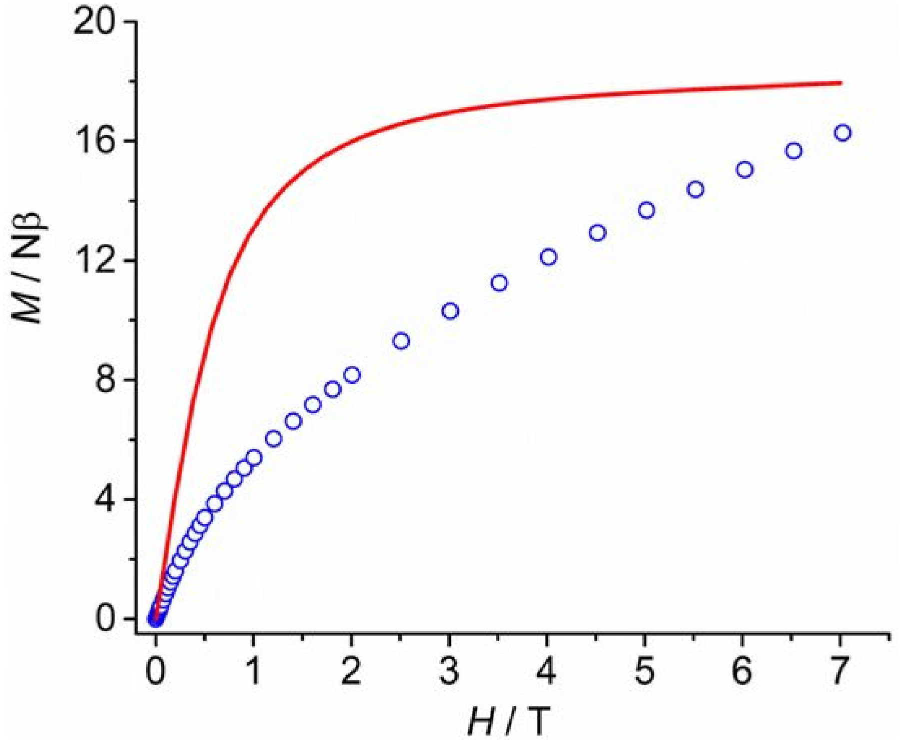

Figure S15. Magnetization versus field data for $\mathbf{1_{Gd}}$ at 2 K. The magnetization is 16.25 Nβ at 7 T and 2 K.

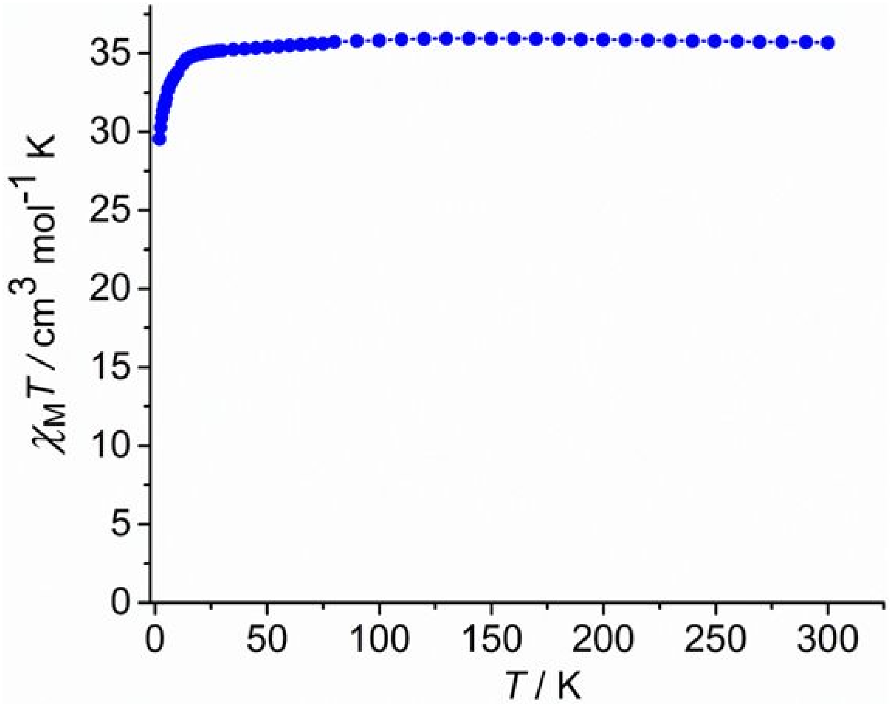

Figure S16. $\chi_M T(T)$ data for $\mathbf{1_{Tb}}$ in an applied field of 1000 Oe. $\chi_M T$ is 35.66 cm^3 K mol^{-1} at 300 K and 29.49 cm^3 K mol^{-1} at 2 K. The solid line is a guide for the eye.

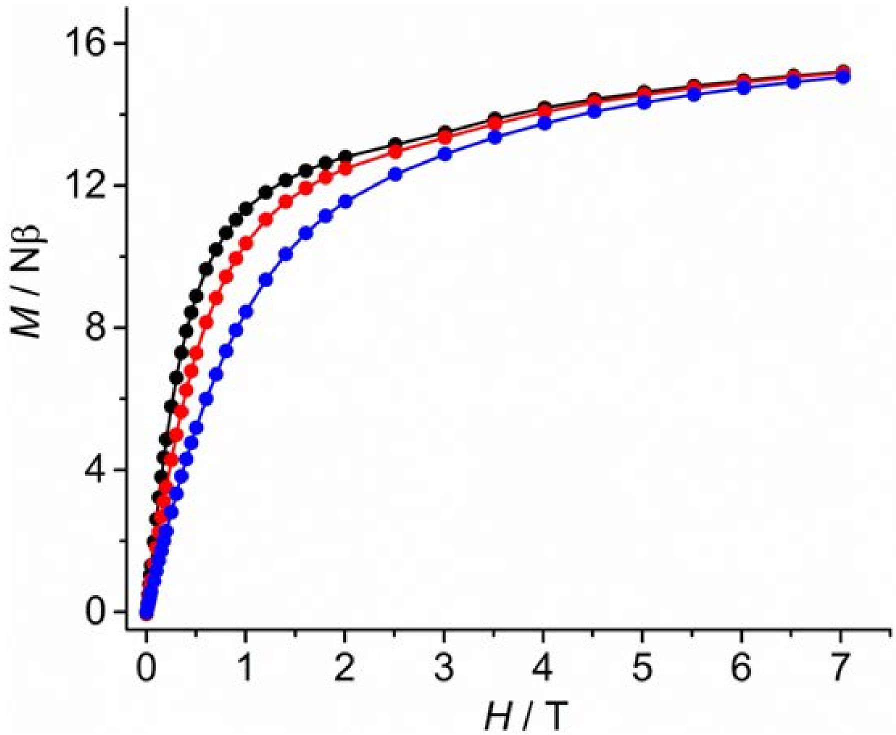

Figure S17. Magnetization versus field data for $\mathbf{1}_{\mathbf{Tb}}$ at 2, 3 and 5 K. The magnetization is 15.25 Nβ at 7 T and 2 K. The solid lines are a guide for the eye.

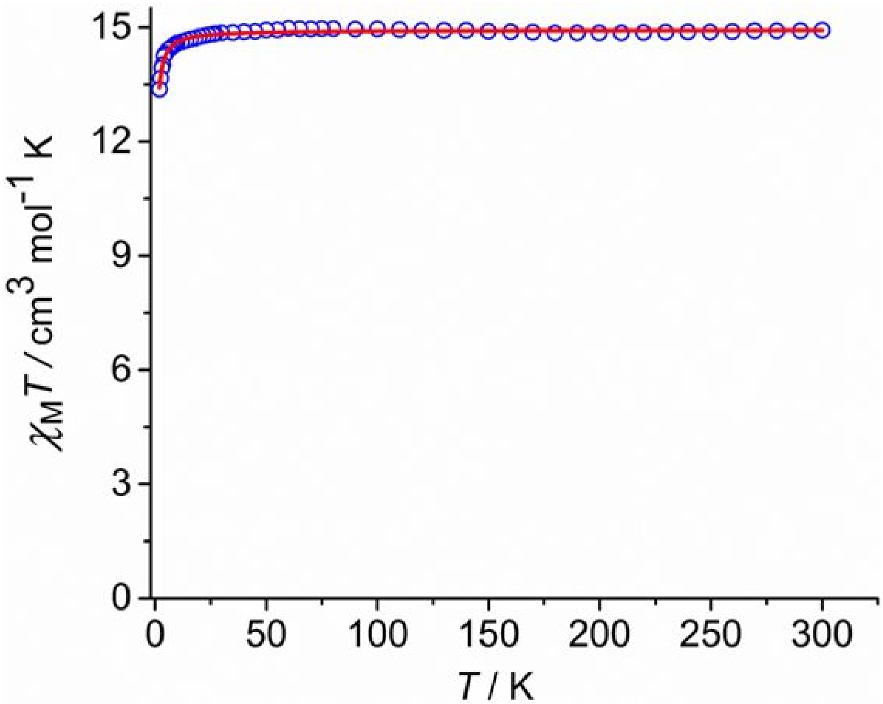

Figure S18. $\chi_M T(T)$ data for $\mathbf{2_{Gd}}$ in an applied field of 1000 Oe. $\chi_M T$ is 14.92 cm^3 K mol^{-1} at 300 K and 9.46 cm^3 K mol^{-1} at 2 K. The red line represents the best fit using the isotropic spin Hamiltonian shown in equation S2 with g = 1.97 and J = –0.001 cm^{-1}.

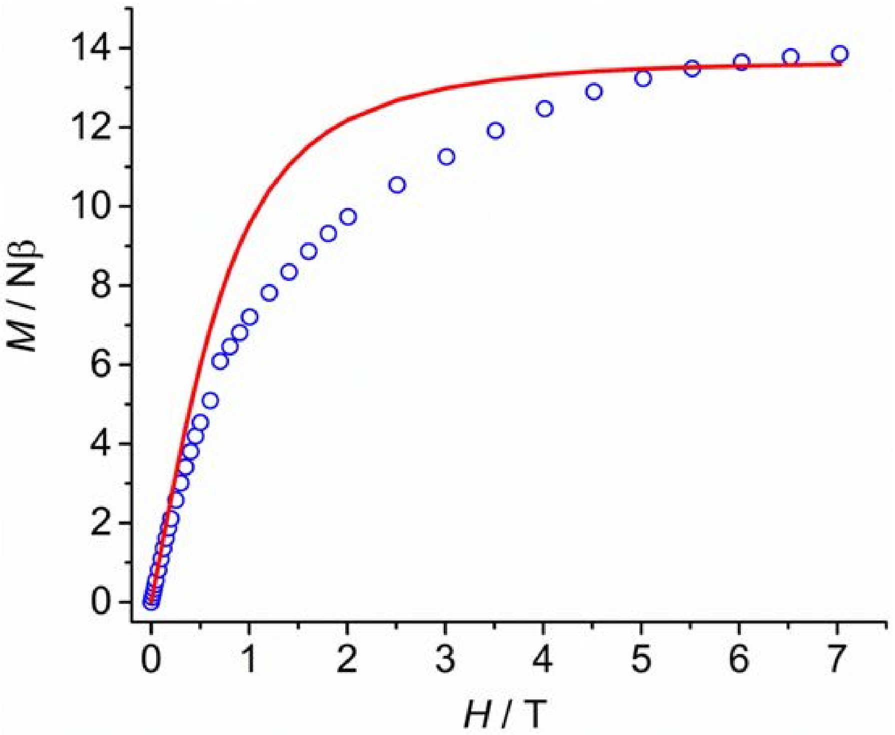

Figure S19. Magnetization versus field data for $\mathbf{2_{Gd}}$ at 2 K. The magnetization is 13.85 Nβ at 7 T and 2 K.

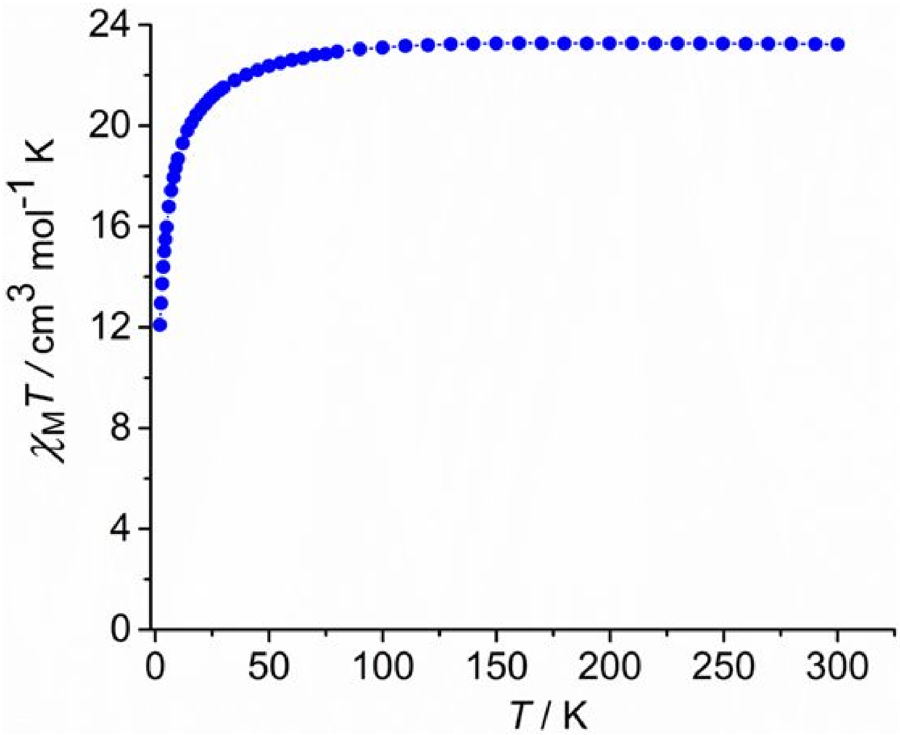

Figure S20. $\chi_M T(T)$ data for $\mathbf{2_{Tb}}$ in an applied field of 1000 Oe. $\chi_M T$ is 23.23 cm^3 K mol^{-1} at 300 K and 12.09 cm^3 K mol^{-1} at 2 K. The solid line is a guide for the eye.

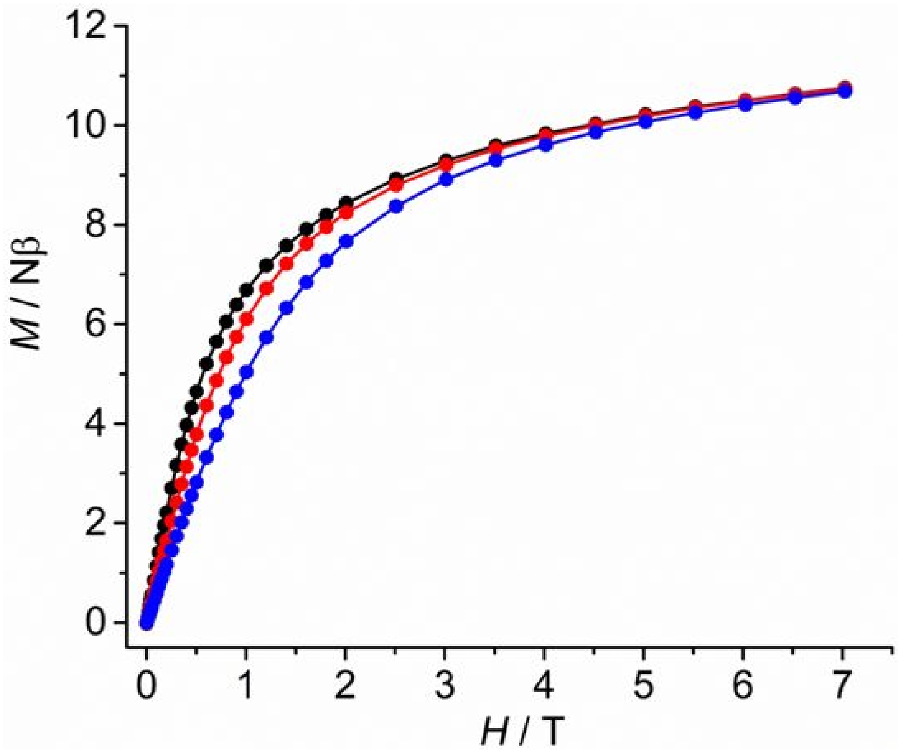

Figure S21. Magnetization versus field data for $\mathbf{2_{Tb}}$ at 2, 3 and 5 K. The magnetization is 10.75 Nβ at 7 T and 2 K. The solid lines are a guide for the eye.

DFT Calculations

Density functional theory (DFT) calculations of the frontier MOs and spin density were performed on the coordinates obtained from the x-ray structures using the ORCA 6.0.0 software package.[8] Hydrogen atom positions were optimized at the DFT level using the pure GGA PBE exchange-correlation functional, keeping constant the position of other atoms.[9, 10] We used the TPSSh functional[11, 12] and the relativistic effects were included with the Douglas-Kroll-Hess Hamiltonian (DKH), together with the scalar relativistic contracted version of the basis functions def2-QZVP for gadolinium, def2-TZVP for yttrium, and def-SVP for oxygen, carbon and hydrogen atoms.[13, 14] The SARC/J auxiliary basis set[15] and RIJCOSX approximation[16, 17] with TightSCF convergence threshold were also used throughout the calculations. The TD-DFT calculations were performed with 50 excited states for both complexes, with a CPCM implicit solvent model for toluene.[18, 19]

The optimization of reactants, transition states, IRC and products were carried out by employing DFT hybrid functional (B3PW91) along with small core pseudopotential for yttrium and molybdenum atoms[20, 21] and a Pople basis set (6-31G**) for the other atoms.[22, 23] Dispersion corrections were included in the calculations by employing D3 version of Grimme's dispersion with Becke-Johnson damping.[24] Solvent effects (toluene) were accounted for using single-point SMD calculations. Frequency calculations were performed to locate saddle points for transition state structures, minima for the other structures and for obtaining thermal corrections over the energies. All the calculations were performed using Gaussian 09 suite of programs.[25]

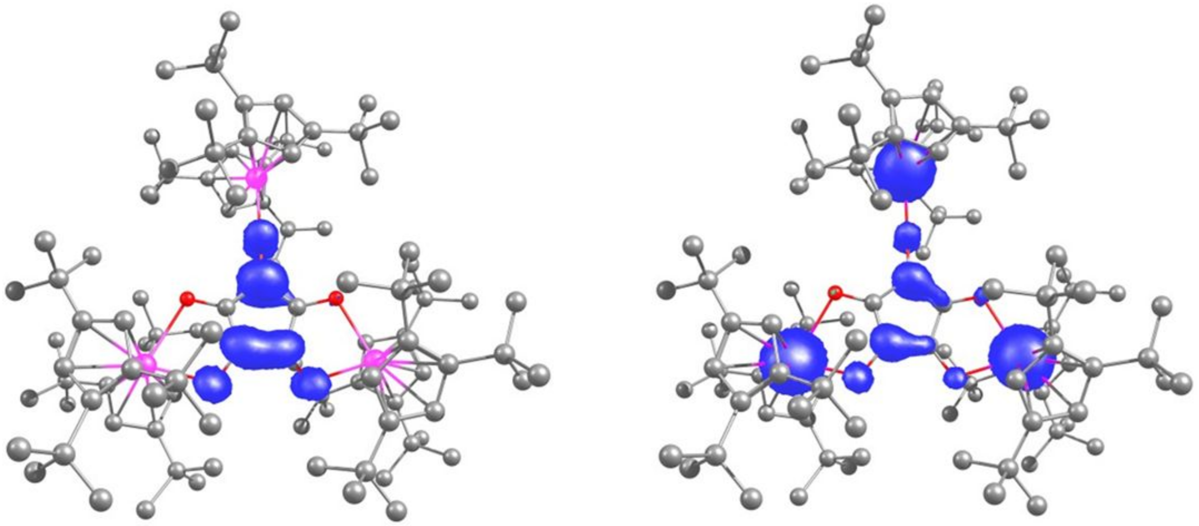

Figure S22. Calculated spin density plots for $\mathbf{1_{Y}}$ (left) and $\mathbf{1_{Gd}}$ (right) using the TPSSh/SARC2-DKH-QZVP/def-TZVP level of theory (isovalue = 0.002).

Table S5. Calculated spin densities of selected atoms for $\mathbf{1_Y}$ and $\mathbf{1_{Gd}}$.

**Atom**	$\mathbf{1_Y}$	**Atom**	$\mathbf{1_{Gd}}$
Y1	0.001793	Gd1	7.119718
Y1A	0.010290	Gd1A	7.102665
Y1AA	0.000120	Gd1AA	7.114026
O1	0.100054	O1	0.127993
O2	0.007642	O2	-0.021493
O3	0.135809	O3	0.111533
O4	-0.005593	O4	0.031983
O5	0.122313	O5	0.045945
C1	0.157122	C1	0.320404
C2	0.008795	C2	-0.097899
C3	0.256957	C3	0.310966
C4	-0.012242	C4	0.051015
C5	0.184361	C5	0.053085

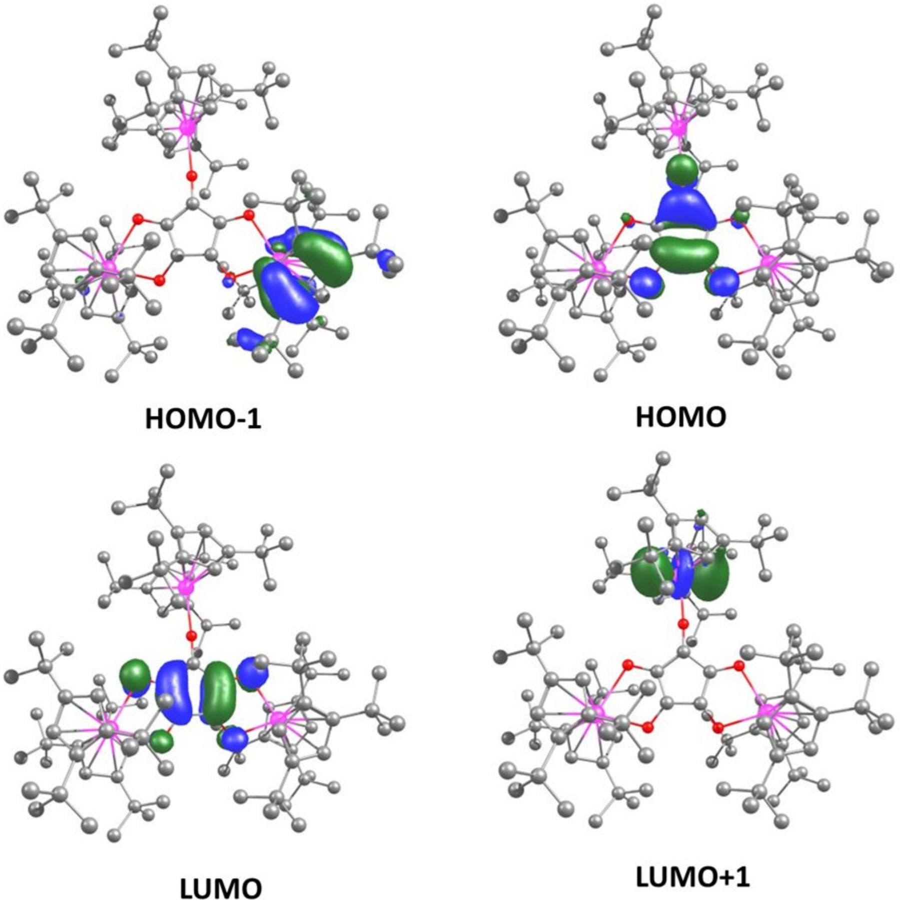

Figure S23. Frontier molecular orbital for $\mathbf{1_Y}$ calculated at the TPSSh/def-TZVP level of theory (isovalue = 0.04).

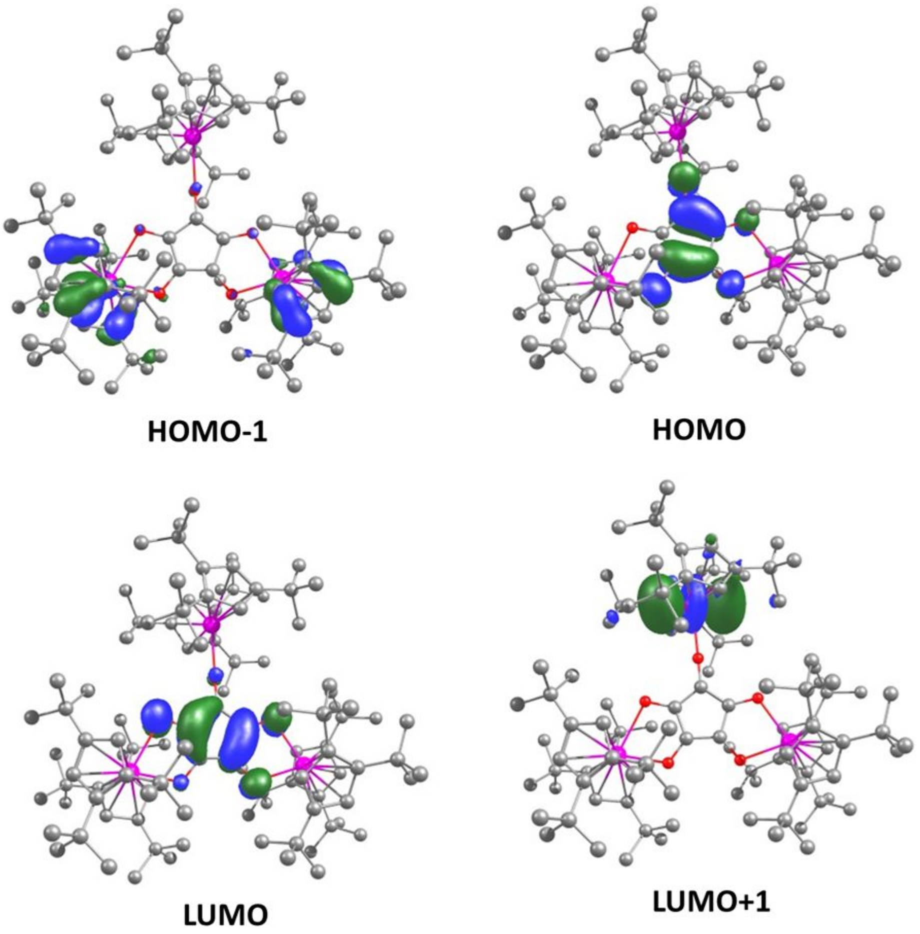

Figure S24. Frontier molecular orbital for $\mathbf{1}_{\mathbf{Gd}}$ calculated at the TPSSh/SARC2-DKH-QZVP level of theory (isovalue = 0.04).

Table S6. Computed major excitation wavelengths (λ) and oscillator strengths (f) in length representation for $\mathbf{1_Y}$.

λ / nm	f	Dominant contribution		
		**Occupied**	**Virtual**	**%Weight**
668	0.00347	485α	487α	74.7
643	0.00789	484α	487α	72.4
562	0.02684	485β	487β	83.4
558	0.044101	483β<br>483α<br>480α<br>482α	486β<br>487α<br>487α<br>487α	30.6<br>17.6<br>11.0<br>10.3
542	0.054097	484β<br>480α<br>482β	487β<br>487α<br>486β	47.0<br>19.1<br>12.0
519	0.023269	483β	487β	88.7
499	0.041620	482β	487β	95.0
417	0.070098	476β	487β	88.6

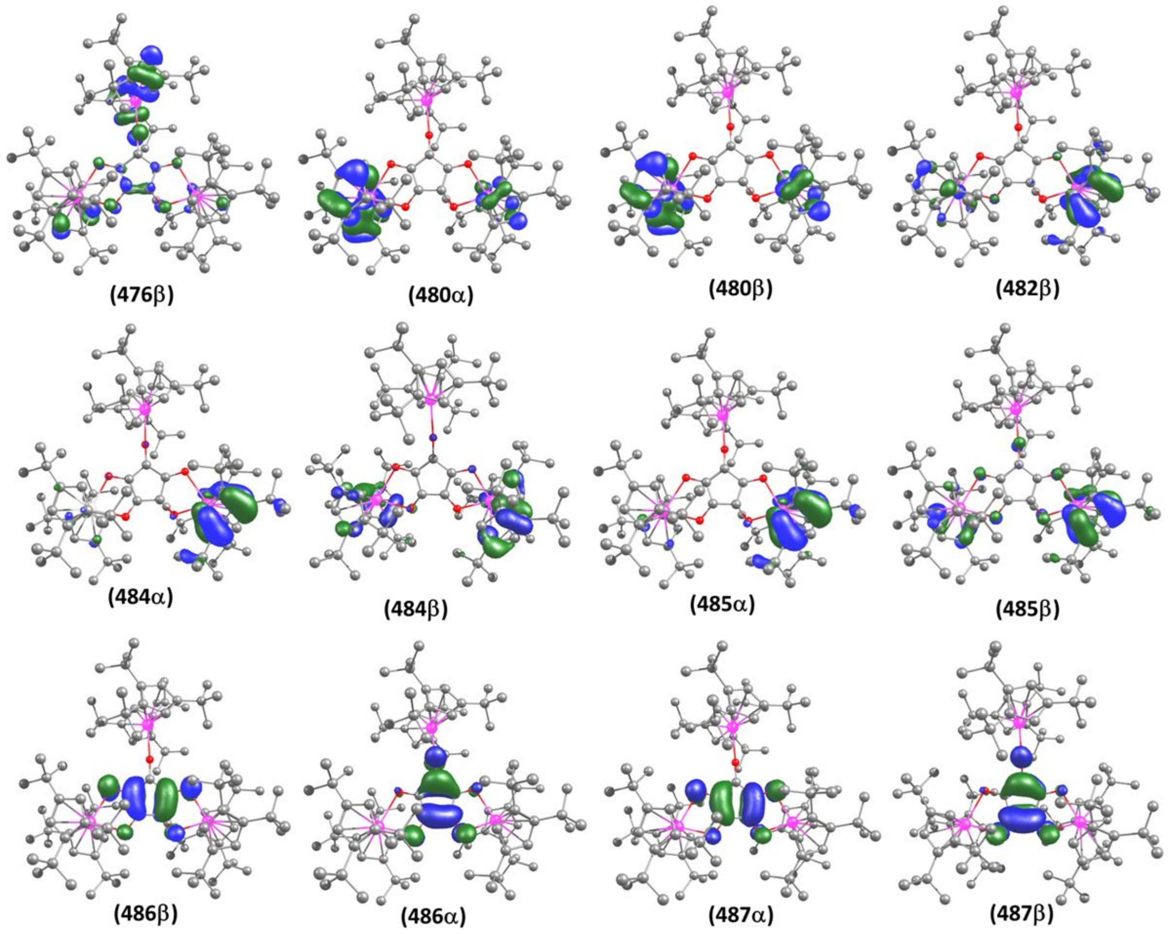

Figure S25. TD-DFT frontier molecular orbital calculated for $\mathbf{1_Y}$ at the TPSSh/def-TZVP level of theory (isovalue = 0.04).

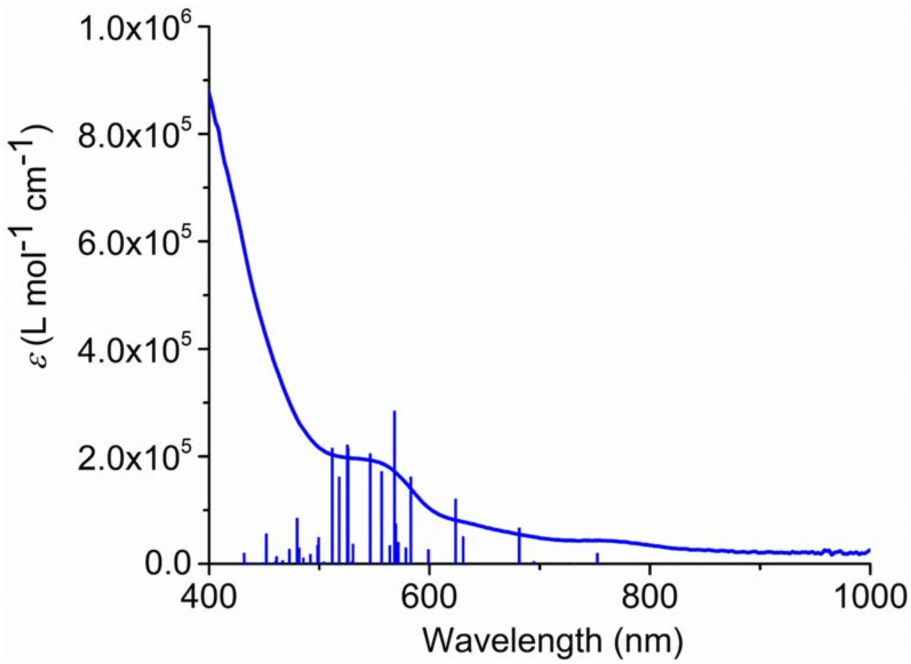

Figure S26. UV/vis spectrum and TD-DFT calculated transitions for $\mathbf{1_{Gd}}$ calculated at the TPSSh/SARC2-DKH-QZVP level of theory.

Table S7. Computed major excitation wavelengths (λ) and oscillator strengths (f) in length representation for $\mathbf{1_{Gd}}$.

λ / nm	f	Dominant contribution		
		**Occupied**	**Virtual**	**%Weight**
753	0.002696	533α<br>531α<br>512β	535α<br>535α<br>513β	43.7<br>14.6<br>16.6
682	0.009291	531α<br>532α<br>533α	535α<br>535α<br>535α	28.0<br>16.6<br>35.7
624	0.016833	511β<br>512β	513β<br>513β	51.1<br>23.6
584	0.022589	512β	514β	86.3
568	0.039729	510β<br>511β	513β<br>514β	31.7<br>33.6
557	0.024011	509β	513β	83.3
547	0.028624	508β	513β	79.4
527	0.0301259	525α<br>510β	535α<br>514β	39.7<br>46.8
526	0.0309323	525α<br>510β	535α<br>514β	38.5<br>40.0
519	0.022594	507β	513β	77.5
512	0.030093	509β	514β	95.7
481	0.0118353	534α<br>507β	538α<br>514β	73.8<br>19.3
453	0.0077455	524α<br>521α	535α<br>535α	70.7<br>12.4

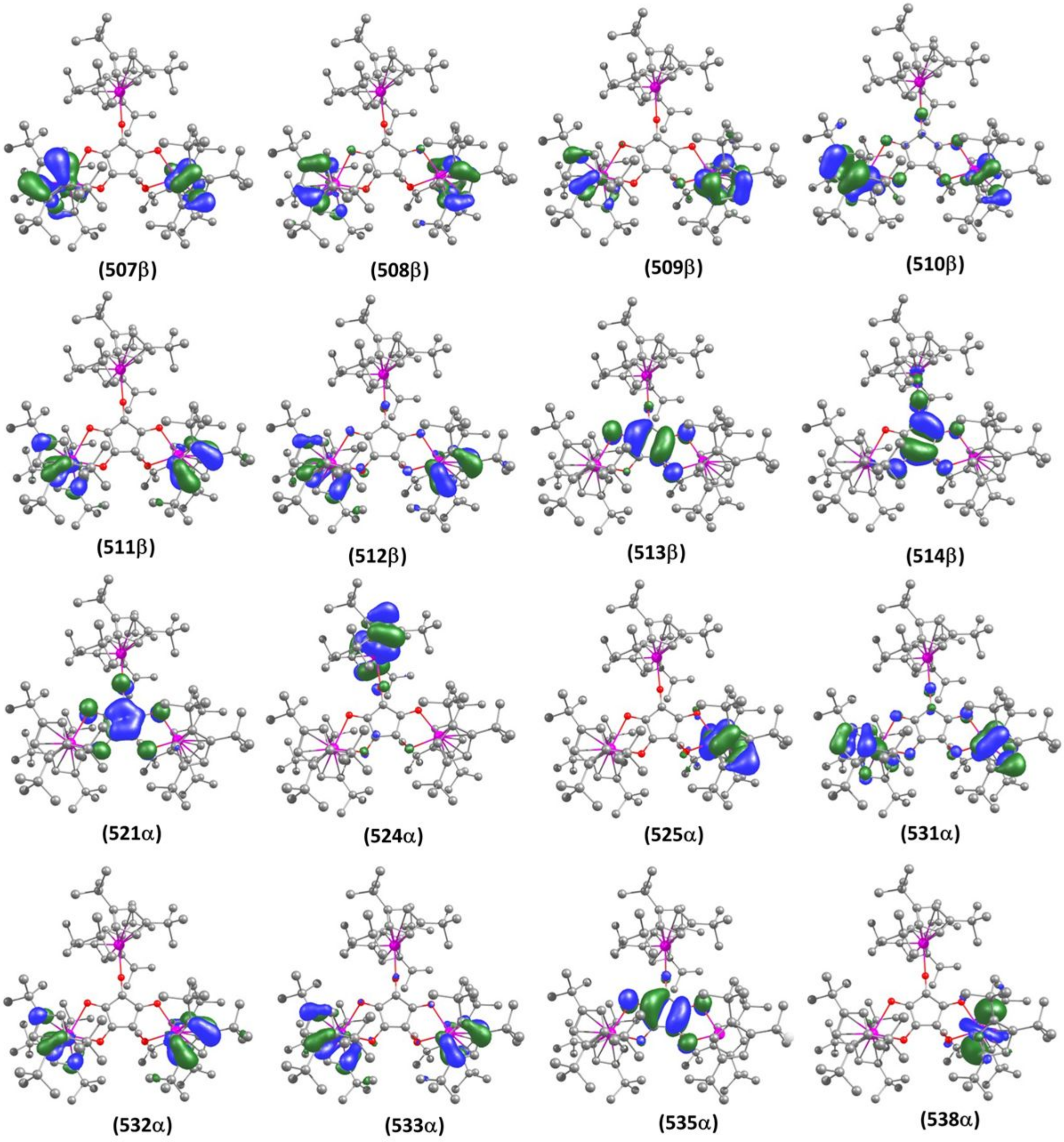

Figure S27. TD-DFT frontier molecular orbital calculated for $\mathbf{1}_{\mathbf{Gd}}$ at the TPSSh/def-TZVP level of theory (isovalue = 0.04).

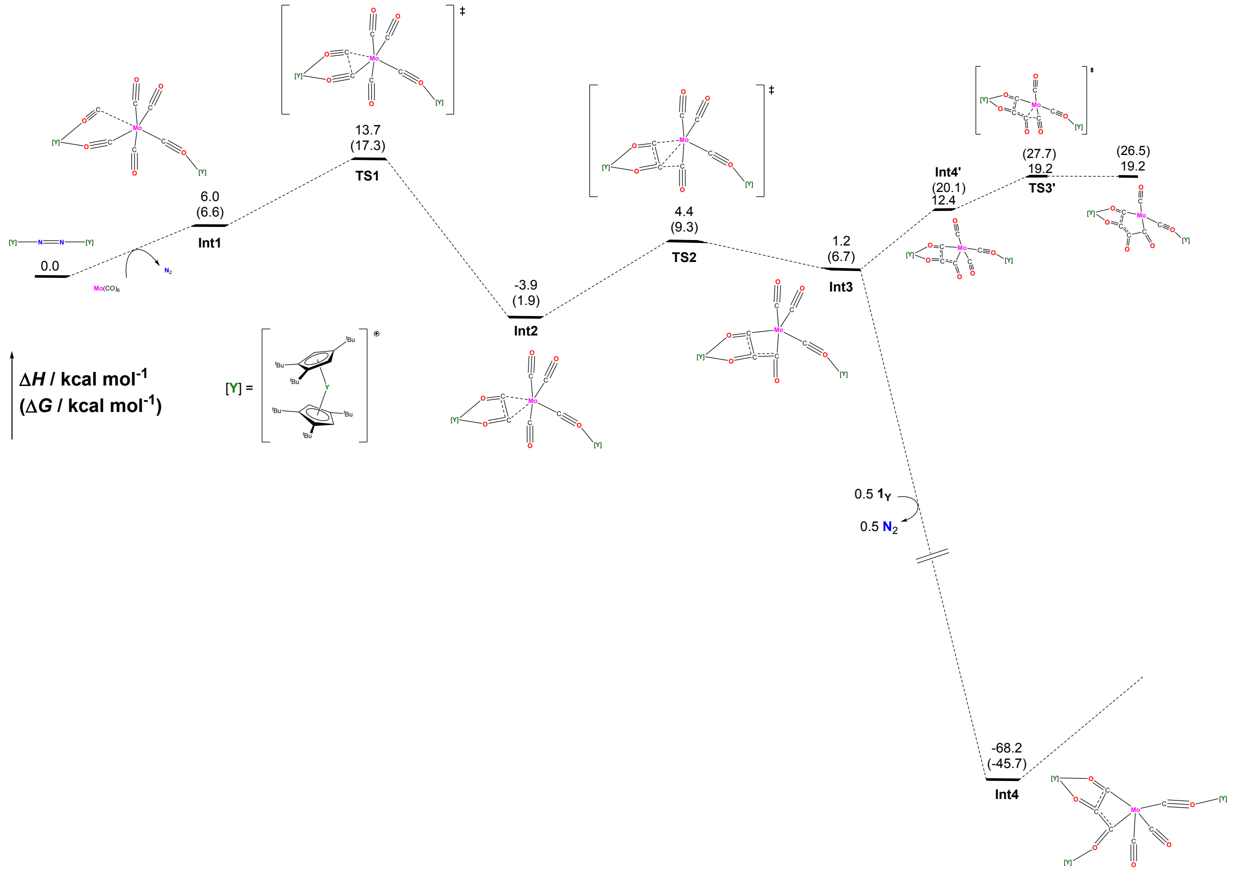

Figure S28. DFT-calculated mechanism for the reaction of [{(Cp^{ttt})$_2$M}$_2$(μ-1,2-N$_2$)] with Mo(CO)$_6$ showing an alternative, unproductive pathway in which the third C–C bond in croconate forms via **TS3'** without a concomitant reduction step. Numbers represent enthalpy changes (kcal mol^{-1}) associated with formation of intermediate complexes and transition states relative to the starting complex [{(Cp^{ttt})$_2$Y}$_2$(μ-1,2-N$_2$)].

Optimized geometries

[{(Cp^{ttt})$_2$Y}$_2$(μ-1,2-N$_2$)]

E= -2847.13943885 Ha

Sum of electronic and thermal Enthalpies= -2845.344499 Ha

Sum of electronic and thermal Free Energies= -2845.548385 Ha

Y	10.299851000	3.944198000	6.746838000
N	11.772209000	3.927034000	5.069969000
C	11.865289000	1.556306000	9.126570000
C	9.341945000	2.012061000	2.962846000
H	8.587668000	2.625583000	2.461305000
H	10.243908000	2.609655000	3.116373000
H	9.592413000	1.183042000	2.295618000
C	7.918751000	2.657631000	6.568429000
C	8.493884000	2.604480000	5.244497000
C	11.622333000	3.034405000	8.896156000
C	8.550520000	3.951261000	4.774635000
H	8.916183000	4.249437000	3.806219000
C	7.987677000	4.832178000	5.721975000
C	7.590254000	0.614190000	3.973035000
H	6.788606000	1.259875000	3.600391000
H	7.825288000	-0.121360000	3.195203000
H	7.206983000	0.067238000	4.834382000
C	13.269906000	1.372404000	9.721651000
H	14.040522000	1.763602000	9.053256000
H	13.355025000	1.900510000	10.676503000
H	13.476771000	0.309992000	9.894913000
C	12.039225000	5.261762000	8.336613000
C	7.911339000	0.233086000	7.514125000
H	7.928366000	-0.262807000	6.545059000
H	8.936267000	0.295113000	7.885623000
H	7.353875000	-0.415057000	8.199087000
C	10.572888000	3.830900000	9.388579000
H	9.764945000	3.473497000	10.008262000
C	7.732358000	6.310681000	5.509740000
C	9.974785000	0.547144000	4.808743000
H	10.190062000	-0.237042000	4.073531000
H	10.886685000	1.137801000	4.947979000
H	9.734983000	0.057754000	5.750412000
C	7.639339000	4.039068000	6.829695000
H	7.136042000	4.401954000	7.712910000
C	10.801606000	5.212347000	9.080274000
C	12.490933000	3.913330000	8.215467000
H	13.400106000	3.611216000	7.722896000
C	9.856123000	7.632775000	9.197122000
H	10.812932000	8.132017000	9.052379000

H	9.357905000	7.563268000	8.228086000
H	9.246005000	8.283359000	9.833031000
C	11.767961000	0.807770000	7.793374000
H	10.765337000	0.917352000	7.370545000
H	12.479213000	1.197260000	7.061967000
H	11.951985000	-0.266509000	7.914868000
C	12.287067000	7.306796000	6.800825000
H	12.044182000	6.709628000	5.914959000
H	11.377020000	7.791057000	7.149899000
H	12.984114000	8.095631000	6.493978000
C	8.841026000	1.447900000	4.298596000
C	6.951377000	6.496572000	4.199610000
H	7.502009000	6.094247000	3.346011000
H	5.987862000	5.979655000	4.249776000
H	6.762468000	7.560092000	4.012802000
C	7.246062000	1.611085000	7.467596000
C	14.204653000	5.852144000	7.220221000
H	14.788317000	5.238802000	7.913182000
H	13.952831000	5.253934000	6.341219000
H	14.838689000	6.682115000	6.896712000
C	13.402994000	7.258435000	9.068675000
H	12.589351000	7.802551000	9.547607000
H	13.870086000	6.618178000	9.823755000
H	14.144451000	7.996481000	8.741523000
C	12.935219000	6.416379000	7.870330000
C	10.841643000	0.981614000	10.108631000
H	10.900837000	1.486784000	11.077989000
H	9.819984000	1.085780000	9.734716000
H	11.029772000	-0.085202000	10.269104000
C	5.792789000	1.449220000	6.975824000
H	5.273714000	2.411910000	7.008989000
H	5.752967000	1.086053000	5.947348000
H	5.248332000	0.741468000	7.612288000
C	9.994648000	6.259882000	9.860460000
C	7.180618000	2.112705000	8.921319000
H	8.173848000	2.369765000	9.294111000
H	6.542467000	2.992973000	9.030651000
H	6.762962000	1.330521000	9.563757000
C	6.901933000	6.891393000	6.656966000
H	5.936027000	6.382821000	6.739303000
H	7.416648000	6.797870000	7.616361000
H	6.711254000	7.955891000	6.484295000
C	9.070707000	7.051576000	5.424769000
H	9.634102000	6.919453000	6.353313000
H	9.689061000	6.675254000	4.607502000
H	8.932134000	8.130053000	5.282468000

C	8.564896000	5.749622000	10.117841000
H	8.077181000	5.457041000	9.186112000
H	8.545642000	4.888027000	10.789845000
H	7.968202000	6.538170000	10.587864000
C	10.667345000	6.435356000	11.237688000
H	10.723741000	5.473431000	11.756121000
H	11.683414000	6.823668000	11.147022000
H	10.089914000	7.129083000	11.860429000
Y	14.035205000	3.944053000	2.500701000
N	12.562764000	3.927000000	4.177486000
C	12.469870000	1.555797000	0.121259000
C	14.993305000	2.012336000	6.284972000
H	15.747594000	2.625941000	6.786400000
H	14.091305000	2.609885000	6.131457000
H	14.742904000	1.183371000	6.952291000
C	16.416385000	2.657618000	2.679263000
C	15.841222000	2.604565000	4.003188000
C	12.712763000	3.033944000	0.351470000
C	15.784541000	3.951393000	4.472920000
H	15.418885000	4.249653000	5.441314000
C	16.347316000	4.832247000	3.525488000
C	16.744941000	0.614384000	5.274779000
H	17.546622000	1.260094000	5.647308000
H	16.509948000	-0.121090000	6.052694000
H	17.128145000	0.067347000	4.413458000
C	11.065340000	1.371764000	-0.473986000
H	10.294619000	1.763050000	0.194237000
H	10.980332000	1.899704000	-1.428939000
H	10.858534000	0.309316000	-0.647096000
C	12.295745000	5.261326000	0.910803000
C	16.423899000	0.233007000	1.733787000
H	16.406844000	-0.262799000	2.702897000
H	15.398986000	0.294990000	1.362246000
H	16.981399000	-0.415189000	1.048904000
C	13.762156000	3.830446000	-0.141041000
H	14.570124000	3.473028000	-0.760680000
C	16.602568000	6.310789000	3.737529000
C	14.360357000	0.547280000	4.439225000
H	14.145147000	-0.236886000	5.174479000
H	13.448443000	1.137932000	4.300052000
H	14.600074000	0.057860000	3.497546000
C	16.695694000	4.039047000	2.417843000
H	17.198961000	4.401863000	1.534581000
C	13.533351000	5.211912000	0.167129000
C	11.844108000	3.912882000	1.032072000
H	10.934967000	3.610766000	1.524693000

C	14.478705000	7.632383000	0.050033000
H	13.521874000	8.131580000	0.194776000
H	14.976974000	7.562995000	1.019052000
H	15.088751000	8.282946000	-0.585966000
C	12.567017000	0.807474000	1.454590000
H	13.569575000	0.917127000	1.877559000
H	11.855644000	1.197073000	2.185824000
H	12.383031000	-0.266828000	1.333243000
C	12.047803000	7.306451000	2.446427000
H	12.290645000	6.709344000	3.332343000
H	12.957870000	7.790681000	2.097364000
H	11.350739000	8.095307000	2.753178000
C	15.494152000	1.448072000	4.949232000
C	17.383596000	6.496875000	5.047607000
H	16.833069000	6.094548000	5.901270000
H	18.347167000	5.980069000	4.997418000
H	17.572393000	7.560431000	5.234328000
C	17.089152000	1.611012000	1.780241000
C	10.130250000	5.851740000	2.027054000
H	9.546486000	5.238561000	1.334037000
H	10.382068000	5.253373000	2.905949000
H	9.496303000	6.681719000	2.350727000
C	10.931993000	7.257922000	0.178525000
H	11.745661000	7.801999000	-0.300407000
H	10.464933000	6.617613000	-0.576532000
H	10.190521000	7.995990000	0.505591000
C	11.399708000	6.415953000	1.376955000
C	13.493650000	0.980982000	-0.860585000
H	13.434514000	1.485971000	-1.830042000
H	14.515267000	1.085287000	-0.486603000
H	13.305615000	-0.085876000	-1.020881000
C	18.542384000	1.449199000	2.272152000
H	19.061456000	2.411890000	2.238952000
H	18.582124000	1.086117000	3.300660000
H	19.086899000	0.741398000	1.635793000
C	14.340244000	6.259417000	-0.613171000
C	17.154735000	2.112524000	0.326490000
H	16.161542000	2.369527000	-0.046436000
H	17.792869000	2.992808000	0.217171000
H	17.572488000	1.330306000	-0.315843000
C	17.432921000	6.891414000	2.590207000
H	18.398841000	6.382871000	2.507869000
H	16.918161000	6.797797000	1.630844000
H	17.623566000	7.955933000	2.762783000
C	15.264182000	7.051626000	3.822446000
H	14.700760000	6.919345000	2.893941000

H	14.645872000	6.675394000	4.639783000
H	15.402713000	8.130129000	3.964588000
C	15.770028000	5.749230000	-0.870527000
H	16.257782000	5.456808000	0.061233000
H	15.789346000	4.887552000	-1.542421000
H	16.366655000	6.537764000	-1.340660000
C	13.667501000	6.434731000	-1.990397000
H	13.611146000	5.472756000	-2.508742000
H	12.651413000	6.822994000	-1.899734000
H	14.244874000	7.128436000	-2.613215000

$Mo(CO)_6$
E= -748.134014133 Ha
Sum of electronic and thermal Enthalpies= -748.068714 Ha
Sum of electronic and thermal Free Energies= -748.127598 Ha

Mo	12.875537000	5.668144000	12.262410000
O	12.393694000	8.510358000	13.655294000
O	12.357573000	6.987242000	9.391474000
O	9.753617000	5.009537000	12.525966000
O	13.394318000	4.349379000	15.133175000
O	15.997813000	6.325650000	12.000643000
O	13.357673000	2.826043000	10.869584000
C	12.566641000	7.490490000	13.155089000
C	13.208328000	4.822929000	14.102881000
C	12.543176000	6.513611000	10.421770000
C	10.874053000	5.246040000	12.431320000
C	14.877247000	6.089522000	12.094851000
C	13.184744000	3.845957000	11.369726000

N_2
E= -109.475104422 Ha
Sum of electronic and thermal Enthalpies= -109.466166 Ha
Sum of electronic and thermal Free Energies= -109.487918 Ha

N	11.796239000	3.904200000	5.032510000
N	12.538761000	3.904200000	4.215090000

Int1
E= -3485.79179619 Ha
Sum of electronic and thermal Enthalpies= -3483.945143 Ha
Sum of electronic and thermal Free Energies= -3484.177343 Ha

Mo	-2.009940000	0.710392000	0.351871000
O	-5.003306000	1.838110000	0.289413000
O	-1.469814000	2.586328000	-2.177361000
O	-2.766522000	-1.585741000	-2.465094000
O	-2.983047000	-1.421438000	2.528881000
O	-0.364202000	2.341720000	2.415432000

O	-0.497516000	-1.655289000	-1.057767000
C	-3.913199000	1.455216000	0.337060000
C	-2.632130000	-0.649164000	1.738687000
C	-1.627894000	1.922790000	-1.238754000
C	-3.307581000	-0.627602000	-2.040767000
C	-1.088883000	1.745455000	1.635496000
C	-0.983140000	-0.708738000	-0.476959000
Y	-1.182431000	-3.319311000	-2.459727000
C	-2.270956000	-2.672312000	-5.952488000
C	-4.911803000	-2.916682000	-0.076708000
H	-4.659606000	-3.224636000	0.941386000
H	-4.289271000	-2.054536000	-0.328327000
H	-5.952316000	-2.577769000	-0.070956000
C	-2.635144000	-5.577028000	-1.908440000
C	-3.298441000	-4.523985000	-1.176084000
C	-1.060441000	-2.802885000	-5.047882000
C	-2.360129000	-4.042591000	-0.215154000
H	-2.553030000	-3.242186000	0.482855000
C	-1.161312000	-4.779114000	-0.277388000
C	-5.588233000	-5.240847000	-0.493830000
H	-5.151402000	-5.570673000	0.454043000
H	-6.616007000	-4.913581000	-0.300356000
H	-5.634438000	-6.104044000	-1.158260000
C	-1.791261000	-2.893358000	-7.398604000
H	-1.027869000	-2.156881000	-7.667979000
H	-1.355140000	-3.889927000	-7.518831000
H	-2.626889000	-2.796746000	-8.101305000
C	0.886312000	-2.240405000	-3.891464000
C	-4.402833000	-6.547608000	-3.587233000
H	-5.279171000	-6.359236000	-2.969104000
H	-4.298715000	-5.731170000	-4.301958000
H	-4.604076000	-7.460319000	-4.158660000
C	-0.295897000	-3.971998000	-4.840982000
H	-0.527046000	-4.941575000	-5.259502000
C	-0.012516000	-4.737355000	0.710985000
C	-5.391336000	-3.596922000	-2.398270000
H	-6.448477000	-3.360310000	-2.234676000
H	-4.906850000	-2.686339000	-2.753528000
H	-5.340632000	-4.340647000	-3.188999000
C	-1.315660000	-5.680943000	-1.353828000
H	-0.603993000	-6.448647000	-1.622101000
C	0.923742000	-3.660193000	-4.154542000
C	-0.345878000	-1.760002000	-4.422454000
H	-0.678102000	-0.734177000	-4.371960000
C	3.225958000	-4.520869000	-3.243809000
H	3.784284000	-3.604549000	-3.431168000

H	2.906277000	-4.528504000	-2.200267000
H	3.924514000	-5.354095000	-3.377715000
C	-2.906173000	-1.283027000	-5.850702000
H	-3.272070000	-1.077154000	-4.842806000
H	-2.193293000	-0.498649000	-6.123192000
H	-3.755852000	-1.212447000	-6.537737000
C	2.549167000	-1.532518000	-2.030793000
H	1.794073000	-1.390292000	-1.256010000
H	2.947874000	-2.537773000	-1.934419000
H	3.371982000	-0.833366000	-1.841244000
C	-4.765983000	-4.076387000	-1.080204000
C	0.046875000	-6.100226000	1.422261000
H	-0.900169000	-6.310952000	1.928225000
H	0.237029000	-6.910219000	0.710965000
H	0.847542000	-6.108325000	2.170902000
C	-3.127175000	-6.754774000	-2.766930000
C	1.346947000	0.175828000	-3.306053000
H	1.011116000	0.557464000	-4.274194000
H	0.509341000	0.216408000	-2.605871000
H	2.118300000	0.860291000	-2.939843000
C	3.054536000	-1.161505000	-4.478010000
H	3.606478000	-2.096585000	-4.580149000
H	2.623006000	-0.921616000	-5.454899000
H	3.773290000	-0.375549000	-4.218564000
C	1.945666000	-1.238359000	-3.411469000
C	-3.314442000	-3.735017000	-5.604377000
H	-2.893545000	-4.740658000	-5.691555000
H	-3.679122000	-3.604091000	-4.580815000
H	-4.180044000	-3.673752000	-6.273318000
C	-3.368601000	-7.932387000	-1.798290000
H	-2.448463000	-8.194733000	-1.268329000
H	-4.122511000	-7.678328000	-1.049172000
H	-3.714498000	-8.815875000	-2.347661000
C	2.046695000	-4.709958000	-4.200712000
C	-2.043276000	-7.182069000	-3.769896000
H	-1.825708000	-6.373855000	-4.472384000
H	-1.108123000	-7.468156000	-3.285280000
H	-2.390736000	-8.047401000	-4.344158000
C	1.315221000	-4.498459000	-0.014941000
H	1.503487000	-5.290642000	-0.746210000
H	1.313102000	-3.536087000	-0.536780000
H	2.154655000	-4.487469000	0.689738000
C	-0.237230000	-3.644265000	1.757626000
H	-0.300285000	-2.654480000	1.302011000
H	-1.160800000	-3.817074000	2.317043000
H	0.592235000	-3.635923000	2.471758000

C	1.486339000	-6.110890000	-3.907822000
H	1.069141000	-6.156617000	-2.898614000
H	0.704792000	-6.408459000	-4.608764000
H	2.288037000	-6.853502000	-3.978350000
C	2.594747000	-4.706672000	-5.643586000
H	1.803456000	-4.948765000	-6.358915000
H	2.995985000	-3.726071000	-5.912493000
H	3.396364000	-5.446727000	-5.751216000
Y	1.650524000	3.292697000	2.540737000
C	2.470636000	4.681718000	-1.012518000
C	1.826320000	-0.841680000	1.931842000
H	2.026750000	-1.485324000	2.793260000
H	0.813086000	-0.440283000	2.011398000
H	1.840047000	-1.464913000	1.034272000
C	3.834552000	2.171941000	3.513871000
C	2.950573000	1.108462000	3.079643000
C	1.943198000	4.889196000	0.398325000
C	1.843538000	1.109836000	3.967704000
H	0.996702000	0.445140000	3.892903000
C	1.978573000	2.129785000	4.932447000
C	4.198583000	-0.468189000	1.444158000
H	4.499095000	-1.132186000	2.260377000
H	4.033967000	-1.085271000	0.555238000
H	5.029940000	0.200488000	1.227375000
C	2.330904000	6.052143000	-1.709319000
H	1.291330000	6.391622000	-1.687138000
H	2.945636000	6.807465000	-1.209563000
H	2.649203000	5.983299000	-2.755761000
C	0.353391000	5.549022000	1.977245000
C	5.517707000	2.799595000	1.687807000
H	5.183580000	2.008684000	1.017524000
H	4.978108000	3.712143000	1.421789000
H	6.581197000	2.970726000	1.488249000
C	2.606091000	5.504059000	1.484393000
H	3.663247000	5.729471000	1.511668000
C	1.134733000	2.276815000	6.183145000
C	2.450627000	1.183033000	0.657631000
H	2.513998000	0.696320000	-0.319245000
H	1.380563000	1.441584000	0.742109000
H	3.071378000	2.087411000	0.586016000
C	3.197041000	2.786816000	4.636066000
H	3.636881000	3.568967000	5.238570000
C	1.655604000	5.948504000	2.466576000
C	0.572159000	4.874783000	0.742699000
H	-0.209837000	4.449860000	0.130148000
C	1.289406000	7.063428000	4.796485000

H	0.252321000	7.345965000	4.628388000
H	1.309268000	6.115124000	5.336872000
H	1.728305000	7.823235000	5.451969000
C	1.628638000	3.661641000	-1.782788000
H	1.598759000	2.690756000	-1.283500000
H	0.597692000	3.997190000	-1.906540000
H	2.043411000	3.511384000	-2.784688000
C	-1.358992000	5.202323000	3.848873000
H	-1.300336000	4.111019000	3.799880000
H	-0.669839000	5.556214000	4.612977000
H	-2.373773000	5.456667000	4.174357000
C	2.895587000	0.254417000	1.803569000
C	1.828983000	1.470926000	7.296994000
H	1.933635000	0.421352000	7.005832000
H	2.829438000	1.867062000	7.498234000
H	1.247342000	1.515309000	8.224838000
C	5.295156000	2.476599000	3.167752000
C	-2.103545000	5.195327000	1.500102000
H	-2.037062000	5.640130000	0.502555000
H	-1.998010000	4.112187000	1.405125000
H	-3.110871000	5.391258000	1.880630000
C	-1.358170000	7.326858000	2.467881000
H	-0.747777000	7.879850000	3.181258000
H	-1.171899000	7.744196000	1.473256000
H	-2.408494000	7.507784000	2.720845000
C	-1.077659000	5.813663000	2.467642000
C	3.944168000	4.275097000	-1.062992000
H	4.576585000	4.960343000	-0.490194000
H	4.104717000	3.263724000	-0.684531000
H	4.295398000	4.294088000	-2.099893000
C	6.163072000	1.286415000	3.616957000
H	6.036578000	1.117334000	4.690771000
H	5.903414000	0.362206000	3.104046000
H	7.221598000	1.497819000	3.426216000
C	2.115641000	6.976781000	3.511139000
C	5.800725000	3.701588000	3.944260000
H	5.223529000	4.596230000	3.703601000
H	5.768999000	3.544712000	5.026183000
H	6.843402000	3.894482000	3.672031000
C	1.031447000	3.739880000	6.616105000
H	2.017803000	4.196656000	6.744352000
H	0.474706000	4.320587000	5.876799000
H	0.499113000	3.821867000	7.569439000
C	-0.276205000	1.717175000	5.965638000
H	-0.788491000	2.221552000	5.141417000
H	-0.253770000	0.647237000	5.739078000

H	-0.875624000	1.849531000	6.872173000
C	3.548985000	6.674250000	3.972760000
H	3.596920000	5.691894000	4.449935000
H	4.274260000	6.698920000	3.156293000
H	3.865797000	7.421912000	4.706985000
C	2.120337000	8.351964000	2.813313000
H	2.787581000	8.338154000	1.946255000
H	1.123834000	8.625644000	2.460561000
H	2.466347000	9.130345000	3.503166000

TS1

E= -3485.77905299 Ha

Sum of electronic and thermal Enthalpies= -3483.932784 Ha

Sum of electronic and thermal Free Energies= -3484.160240 Ha

Imag_freq=-437.0 cm-1

Mo	13.964374000	5.326428000	11.761918000
O	11.246483000	7.034114000	12.148523000
O	14.819410000	7.365637000	9.457647000
O	13.334752000	2.900119000	9.575658000
O	13.277254000	3.607406000	14.369174000
O	15.624073000	7.173872000	13.661842000
O	15.630208000	2.834708000	10.643309000
C	12.199197000	6.408300000	11.982430000
C	13.488049000	4.202862000	13.404992000
C	14.509122000	6.601891000	10.265757000
C	13.255708000	3.866382000	10.325681000
C	14.906595000	6.487203000	12.958316000
C	15.092385000	3.833565000	11.094193000
Y	14.846758000	1.246594000	9.165310000
C	13.431126000	2.386632000	5.963238000
C	11.396264000	1.356416000	11.989462000
H	11.779678000	0.946762000	12.928328000
H	11.955161000	2.262164000	11.744540000
H	10.355790000	1.651962000	12.159264000
C	13.466811000	-1.076086000	9.665008000
C	12.889542000	-0.112796000	10.572608000
C	14.741993000	2.126170000	6.679140000
C	13.929119000	0.280016000	11.464446000
H	13.817545000	1.004973000	12.256202000
C	15.115632000	-0.429813000	11.189325000
C	10.646623000	-0.894541000	11.349794000
H	11.139592000	-1.333971000	12.223156000
H	9.636682000	-0.588096000	11.645523000
H	10.548134000	-1.677381000	10.597410000
C	13.724701000	2.418604000	4.452664000
H	14.451829000	3.202648000	4.219087000

H	14.139351000	1.463930000	4.113680000
H	12.808530000	2.617003000	3.884232000
C	16.812124000	2.503727000	7.683912000
C	11.551658000	-1.815815000	8.035755000
H	10.732000000	-1.677531000	8.738554000
H	11.625927000	-0.925677000	7.410281000
H	11.278960000	-2.653742000	7.384837000
C	15.496441000	0.932559000	6.637121000
H	15.199944000	0.033197000	6.115450000
C	16.370977000	-0.475720000	12.036039000
C	10.732355000	0.982772000	9.654337000
H	9.698587000	1.221754000	9.928921000
H	11.233137000	1.914942000	9.385242000
H	10.698695000	0.342816000	8.776506000
C	14.837099000	-1.228400000	10.058972000
H	15.516291000	-1.954530000	9.633926000
C	16.787158000	1.135254000	7.227033000
C	15.542296000	3.065357000	7.360570000
H	15.237154000	4.073589000	7.595131000
C	19.129922000	0.119321000	7.811737000
H	19.696660000	1.043009000	7.704447000
H	18.894448000	-0.025328000	8.867184000
H	19.790941000	-0.699726000	7.507768000
C	12.819795000	3.726851000	6.382288000
H	12.603494000	3.748053000	7.452840000
H	13.492031000	4.558804000	6.148980000
H	11.883624000	3.895503000	5.839307000
C	18.634124000	2.948694000	9.472189000
H	17.921781000	2.963867000	10.299817000
H	19.052969000	1.948847000	9.399203000
H	19.458125000	3.631785000	9.713054000
C	11.442176000	0.324989000	10.846813000
C	16.446585000	-1.860101000	12.702778000
H	15.557775000	-2.039648000	13.315406000
H	16.503421000	-2.654663000	11.952173000
H	17.331046000	-1.932572000	13.346804000
C	12.882514000	-2.138642000	8.720172000
C	17.408629000	4.825294000	8.485962000
H	16.989725000	5.310348000	7.600111000
H	16.649067000	4.812641000	9.270665000
H	18.237761000	5.448484000	8.834661000
C	18.973086000	3.588146000	7.048702000
H	19.474886000	2.656331000	6.787021000
H	18.480313000	3.964681000	6.146758000
H	19.742947000	4.310424000	7.345231000
C	17.943866000	3.415246000	8.181597000

C	12.433513000	1.263970000	6.256786000
H	12.837869000	0.290277000	5.964205000
H	12.189657000	1.231532000	7.323340000
H	11.495781000	1.415538000	5.710327000
C	12.698192000	-3.425022000	9.552563000
H	13.651792000	-3.749574000	9.979305000
H	12.002893000	-3.264734000	10.380419000
H	12.305575000	-4.235499000	8.926889000
C	17.870423000	0.083164000	6.941794000
C	13.873986000	-2.444301000	7.585498000
H	14.080078000	-1.542733000	7.003101000
H	14.826011000	-2.834129000	7.949984000
H	13.450059000	-3.197341000	6.912607000
C	17.610205000	-0.275257000	11.159562000
H	17.667777000	-1.044103000	10.383049000
H	17.589690000	0.704876000	10.672372000
H	18.530364000	-0.328789000	11.753080000
C	16.333634000	0.600349000	13.123342000
H	16.223979000	1.595260000	12.687678000
H	15.500189000	0.435180000	13.813323000
H	17.260362000	0.579805000	13.706489000
C	17.291714000	-1.332099000	7.100968000
H	16.930749000	-1.487136000	8.121143000
H	16.462216000	-1.527192000	6.419245000
H	18.066992000	-2.077406000	6.893258000
C	18.294670000	0.263867000	5.469350000
H	17.436366000	0.138738000	4.802989000
H	18.705728000	1.261635000	5.295655000
H	19.056738000	-0.474938000	5.193985000
Y	17.636866000	8.049618000	14.017480000
C	18.878444000	9.328355000	10.533528000
C	17.730761000	3.884413000	13.502107000
H	17.800063000	3.252533000	14.392234000
H	16.734687000	4.330488000	13.455375000
H	17.819613000	3.242808000	12.622579000
C	19.656687000	6.868003000	15.236507000
C	18.790293000	5.826201000	14.724551000
C	18.212950000	9.579699000	11.877291000
C	17.590214000	5.886077000	15.481500000
H	16.735781000	5.245051000	15.329187000
C	17.653028000	6.922105000	16.436557000
C	20.158292000	4.155043000	13.291432000
H	20.333145000	3.502733000	14.152391000
H	20.076146000	3.520749000	12.402714000
H	21.035481000	4.786608000	13.160618000
C	18.830489000	10.681606000	9.792328000

H	17.798636000	11.031251000	9.694058000
H	19.396153000	11.443764000	10.337329000
H	19.259617000	10.583576000	8.788561000
C	16.478457000	10.322481000	13.255602000
C	21.531939000	7.399118000	13.579556000
H	21.268331000	6.584915000	12.905740000
H	21.026302000	8.301904000	13.227172000
H	22.611107000	7.563369000	13.487855000
C	18.769254000	10.210360000	13.012712000
H	19.822771000	10.414854000	13.147884000
C	16.669384000	7.139281000	17.568723000
C	18.578118000	5.831748000	12.253359000
H	18.701134000	5.289548000	11.311018000
H	17.523264000	6.154272000	12.230722000
H	19.249068000	6.699750000	12.204268000
C	18.920334000	7.530675000	16.267739000
H	19.318414000	8.310835000	16.900751000
C	17.729961000	10.704391000	13.874019000
C	16.813160000	9.608196000	12.070660000
H	16.091818000	9.193644000	11.382537000
C	17.132666000	11.918348000	16.107734000
H	16.137707000	12.236488000	15.803331000
H	17.043252000	10.993833000	16.681639000
H	17.522297000	12.685795000	16.785123000
C	18.105807000	8.297631000	9.705887000
H	18.031633000	7.335673000	10.216982000
H	17.090589000	8.632348000	9.483629000
H	18.610944000	8.129318000	8.749384000
C	14.548202000	10.116294000	14.932447000
H	14.572261000	9.022631000	14.939177000
H	15.156461000	10.484354000	15.755751000
H	13.512806000	10.418956000	15.123371000
C	18.851682000	4.934829000	13.474529000
C	17.185140000	6.345261000	18.783136000
H	17.280817000	5.283495000	18.536445000
H	18.168726000	6.708671000	19.097553000
H	16.495359000	6.442018000	19.629311000
C	21.152107000	7.129405000	15.037758000
C	14.079335000	10.009428000	12.520865000
H	14.273696000	10.391677000	11.514436000
H	14.159933000	8.920248000	12.504951000
H	13.042763000	10.255187000	12.771453000
C	14.786597000	12.167697000	13.463919000
H	15.336380000	12.734835000	14.214627000
H	15.098151000	12.528912000	12.478640000
H	13.722556000	12.396609000	13.588202000

C	15.010481000	10.648508000	13.567403000
C	20.342458000	8.900432000	10.644960000
H	20.921932000	9.588773000	11.267788000
H	20.445941000	7.895201000	11.058151000
H	20.800347000	8.893430000	9.650347000
C	21.939797000	5.937932000	15.612505000
H	21.699571000	5.809462000	16.672514000
H	21.710792000	5.001782000	15.106157000
H	23.017263000	6.119399000	15.525772000
C	18.104394000	11.748861000	14.937868000
C	21.599212000	8.371820000	15.822044000
H	21.063893000	9.264901000	15.493800000
H	21.454816000	8.253023000	16.899598000
H	22.667288000	8.540611000	15.651758000
C	16.573315000	8.619639000	17.941117000
H	17.554154000	9.045322000	18.175104000
H	16.134258000	9.193467000	17.121031000
H	15.932627000	8.754868000	18.818709000
C	15.274430000	6.627026000	17.189737000
H	14.899465000	7.116363000	16.285994000
H	15.276506000	5.548027000	17.009594000
H	14.567389000	6.823492000	18.002126000
C	19.455967000	11.404062000	15.581671000
H	19.400800000	10.441175000	16.096887000
H	20.274289000	11.364313000	14.859105000
H	19.717279000	12.167404000	16.321572000
C	18.255423000	13.096871000	14.204244000
H	19.021927000	13.024951000	13.426794000
H	17.322163000	13.397444000	13.722962000
H	18.549241000	13.885115000	14.906971000

Int2

E= -3485.80676162 Ha

Sum of electronic and thermal Enthalpies= -3483.958367 Ha

Sum of electronic and thermal Free Energies= -3484.186983 Ha

Mo	-2.205309000	0.547005000	0.422950000
O	-5.285127000	1.322699000	0.781496000
O	-2.056089000	2.877131000	-1.767796000
O	-2.597722000	-1.581546000	-2.145956000
O	-2.560585000	-1.509629000	2.853430000
O	-0.537614000	2.334978000	2.375653000
O	-0.145342000	-1.825941000	-0.694217000
C	-4.160648000	1.090182000	0.676773000
C	-2.449032000	-0.794841000	1.955174000
C	-2.079220000	2.020738000	-0.993270000
C	-2.290379000	-0.863964000	-1.148328000

C	-1.226158000	1.646149000	1.658981000
C	-1.004452000	-0.908395000	-0.482031000
Y	-1.021150000	-3.296936000	-2.338268000
C	-2.149232000	-2.142816000	-5.660654000
C	-4.715766000	-3.077422000	0.150186000
H	-4.433391000	-3.502021000	1.118060000
H	-4.096692000	-2.199425000	-0.044875000
H	-5.752815000	-2.735715000	0.231244000
C	-2.556448000	-5.579580000	-2.013459000
C	-3.172371000	-4.605421000	-1.144968000
C	-0.909813000	-2.473495000	-4.853550000
C	-2.200085000	-4.269184000	-0.158911000
H	-2.352129000	-3.554119000	0.634804000
C	-1.024667000	-5.024574000	-0.338733000
C	-5.501422000	-5.296648000	-0.549391000
H	-5.098274000	-5.752778000	0.360817000
H	-6.517766000	-4.947088000	-0.334583000
H	-5.572818000	-6.076618000	-1.307414000
C	-1.750902000	-2.152144000	-7.147372000
H	-0.961523000	-1.418346000	-7.338460000
H	-1.375592000	-3.135921000	-7.446814000
H	-2.611993000	-1.905288000	-7.779445000
C	1.109116000	-2.207541000	-3.719298000
C	-4.377100000	-6.258588000	-3.775179000
H	-5.240913000	-6.085154000	-3.135874000
H	-4.223405000	-5.375225000	-4.395681000
H	-4.632508000	-7.089467000	-4.442165000
C	-0.234502000	-3.712693000	-4.823031000
H	-0.558320000	-4.602430000	-5.343620000
C	0.147597000	-5.129912000	0.615056000
C	-5.203479000	-3.423929000	-2.228837000
H	-6.247284000	-3.148723000	-2.037601000
H	-4.646837000	-2.509032000	-2.444972000
H	-5.188685000	-4.057188000	-3.112159000
C	-1.231368000	-5.790972000	-1.505803000
H	-0.547361000	-6.540860000	-1.878717000
C	1.026222000	-3.580822000	-4.153040000
C	-0.097094000	-1.573527000	-4.137300000
H	-0.355632000	-0.543924000	-3.941572000
C	3.258141000	-4.731332000	-3.406342000
H	3.889823000	-3.846718000	-3.473752000
H	2.936982000	-4.851731000	-2.370475000
H	3.885812000	-5.592965000	-3.658834000
C	-2.702189000	-0.760663000	-5.299952000
H	-2.979968000	-0.708866000	-4.244419000
H	-1.967849000	0.024934000	-5.506417000

H	-3.591326000	-0.547635000	-5.903220000
C	2.829431000	-1.852951000	-1.819732000
H	2.061627000	-1.784274000	-1.045319000
H	3.187845000	-2.877660000	-1.855759000
H	3.676537000	-1.219309000	-1.529499000
C	-4.619263000	-4.111949000	-0.986185000
C	0.137768000	-6.540278000	1.229230000
H	-0.806348000	-6.724605000	1.751042000
H	0.250408000	-7.307348000	0.456570000
H	0.957917000	-6.654847000	1.947921000
C	-3.114520000	-6.627420000	-2.991655000
C	1.783144000	0.077817000	-2.898056000
H	1.461968000	0.578174000	-3.815986000
H	0.961324000	0.110148000	-2.177117000
H	2.616934000	0.658048000	-2.488944000
C	3.370113000	-1.258362000	-4.217737000
H	3.841512000	-2.218184000	-4.429313000
H	2.961247000	-0.874358000	-5.157819000
H	4.153236000	-0.570399000	-3.877401000
C	2.258765000	-1.361212000	-3.156914000
C	-3.231744000	-3.197853000	-5.425017000
H	-2.873455000	-4.196128000	-5.693719000
H	-3.538608000	-3.210850000	-4.375255000
H	-4.123624000	-2.988930000	-6.026465000
C	-3.413327000	-7.891122000	-2.156520000
H	-2.503510000	-8.252822000	-1.668618000
H	-4.148595000	-7.686000000	-1.374923000
H	-3.805156000	-8.691302000	-2.795750000
C	2.065717000	-4.692735000	-4.366073000
C	-2.061727000	-7.004804000	-4.045811000
H	-1.794415000	-6.139635000	-4.657074000
H	-1.145861000	-7.402140000	-3.605288000
H	-2.464666000	-7.777364000	-4.709536000
C	1.464858000	-4.923264000	-0.138135000
H	1.578144000	-5.668588000	-0.931232000
H	1.507435000	-3.926854000	-0.588603000
H	2.325708000	-5.017227000	0.534212000
C	0.026070000	-4.096112000	1.736319000
H	-0.028219000	-3.085243000	1.328462000
H	-0.870107000	-4.272654000	2.338952000
H	0.894839000	-4.155119000	2.400817000
C	1.399808000	-6.073576000	-4.261418000
H	0.958793000	-6.212954000	-3.271447000
H	0.612812000	-6.218789000	-5.002836000
H	2.145646000	-6.859912000	-4.419322000
C	2.604857000	-4.533875000	-5.803088000

H	1.789855000	-4.600598000	-6.529573000
H	3.091649000	-3.565040000	-5.939892000
H	3.335082000	-5.320069000	-6.029288000
Y	1.515160000	3.287338000	2.492844000
C	2.257365000	4.393918000	-1.167732000
C	1.500426000	-0.836658000	2.195199000
H	1.674162000	-1.470803000	3.069418000
H	0.525615000	-0.355027000	2.292502000
H	1.432383000	-1.471736000	1.309795000
C	3.670486000	2.193756000	3.518802000
C	2.743153000	1.127465000	3.199721000
C	1.787837000	4.721331000	0.240100000
C	1.659538000	1.242289000	4.108491000
H	0.798050000	0.592730000	4.118030000
C	1.852250000	2.336016000	4.980753000
C	3.899038000	-0.658820000	1.732498000
H	4.159705000	-1.243042000	2.620265000
H	3.697564000	-1.359355000	0.917252000
H	4.767680000	-0.066976000	1.448723000
C	2.167112000	5.722941000	-1.948282000
H	1.149365000	6.122606000	-1.914185000
H	2.840750000	6.472462000	-1.521012000
H	2.442448000	5.568719000	-2.997760000
C	0.273562000	5.535866000	1.820562000
C	5.336720000	2.566094000	1.601976000
H	4.996684000	1.705831000	1.027104000
H	4.798665000	3.446324000	1.239839000
H	6.398812000	2.710152000	1.376036000
C	2.505517000	5.386367000	1.260249000
H	3.570447000	5.572984000	1.241719000
C	1.064223000	2.588417000	6.251729000
C	2.263305000	1.020497000	0.770469000
H	2.333658000	0.447476000	-0.157150000
H	1.202180000	1.321985000	0.798615000
H	2.914914000	1.895568000	0.640093000
C	3.082401000	2.924115000	4.599161000
H	3.559827000	3.740706000	5.121291000
C	1.602069000	5.928360000	2.238115000
C	0.429660000	4.776314000	0.627087000
H	-0.384856000	4.346784000	0.063276000
C	1.360162000	7.203921000	4.501995000
H	0.315366000	7.476610000	4.368377000
H	1.408601000	6.297720000	5.109038000
H	1.831341000	8.008079000	5.077005000
C	1.333191000	3.375309000	-1.840052000
H	1.261663000	2.445386000	-1.270966000

H	0.322586000	3.769627000	-1.962384000
H	1.709637000	3.126679000	-2.836671000
C	-1.346876000	5.315599000	3.776812000
H	-1.311140000	4.221404000	3.775392000
H	-0.608152000	5.686971000	4.484002000
H	-2.337295000	5.602282000	4.147264000
C	2.640172000	0.173513000	2.001419000
C	1.754632000	1.784011000	7.370101000
H	1.784945000	0.719634000	7.118573000
H	2.784086000	2.126125000	7.515901000
H	1.215306000	1.899909000	8.317022000
C	5.131287000	2.415386000	3.112499000
C	-2.208673000	5.201888000	1.472692000
H	-2.175663000	5.576234000	0.445435000
H	-2.127298000	4.113245000	1.450383000
H	-3.194561000	5.445111000	1.880843000
C	-1.393445000	7.369680000	2.290662000
H	-0.743534000	7.949526000	2.944765000
H	-1.250647000	7.732723000	1.268040000
H	-2.428228000	7.577779000	2.583259000
C	-1.129543000	5.855635000	2.355042000
C	3.704609000	3.903865000	-1.236438000
H	4.390431000	4.585071000	-0.723326000
H	3.823007000	2.907938000	-0.805494000
H	4.022782000	3.844510000	-2.282238000
C	5.972185000	1.255379000	3.677789000
H	5.874877000	1.221233000	4.767423000
H	5.664388000	0.286664000	3.288039000
H	7.030614000	1.402027000	3.433729000
C	2.119245000	7.022220000	3.184940000
C	5.686543000	3.703630000	3.736776000
H	5.135928000	4.583233000	3.398221000
H	5.666327000	3.675440000	4.829767000
H	6.730764000	3.828375000	3.433483000
C	1.060946000	4.068639000	6.636376000
H	2.074238000	4.477928000	6.697581000
H	0.493107000	4.656496000	5.911780000
H	0.588724000	4.206985000	7.614457000
C	-0.383868000	2.103916000	6.112296000
H	-0.901933000	2.608064000	5.291233000
H	-0.433788000	1.026758000	5.929236000
H	-0.937390000	2.305525000	7.035182000
C	3.573672000	6.745529000	3.592045000
H	3.647372000	5.797197000	4.129810000
H	4.256689000	6.715908000	2.740024000
H	3.926754000	7.539100000	4.258157000

C	2.094316000	8.344699000	2.391710000
H	2.712396000	8.261439000	1.492659000
H	1.082547000	8.606114000	2.075750000
H	2.485560000	9.165062000	3.004233000

TS2

E= -3485.79414172 Ha

Sum of electronic and thermal Enthalpies= -3483.946172 Ha

Sum of electronic and thermal Free Energies= -3484.169549 Ha

Imag_freq=-59.1 cm-1

Mo	-1.797920000	-0.380765000	1.690982000
O	-4.775070000	-0.257747000	2.871509000
O	-3.355799000	1.053025000	-0.701728000
O	-1.749948000	-1.000558000	-1.699509000
O	-1.292963000	-2.481161000	4.057774000
O	-0.387128000	1.797161000	3.376158000
O	0.143846000	-2.218198000	-0.166550000
C	-3.695581000	-0.274920000	2.474789000
C	-1.475487000	-1.748146000	3.188384000
C	-2.651096000	0.473253000	0.061010000
C	-1.718236000	-0.808124000	-0.432566000
C	-0.992171000	0.941409000	2.765991000
C	-0.644624000	-1.377222000	0.368382000
Y	-0.727590000	-3.021340000	-2.200734000
C	-0.960953000	-1.075397000	-5.302688000
C	-4.737304000	-2.505242000	-0.112071000
H	-4.758358000	-3.224854000	0.712397000
H	-3.909663000	-1.817842000	0.055540000
H	-5.663317000	-1.923485000	-0.073835000
C	-2.784249000	-4.872366000	-2.587374000
C	-3.311469000	-3.984390000	-1.578242000
C	0.002995000	-1.899960000	-4.472685000
C	-2.457228000	-4.100152000	-0.444530000
H	-2.582096000	-3.552838000	0.478393000
C	-1.449789000	-5.059636000	-0.675598000
C	-5.822906000	-4.172516000	-1.523009000
H	-5.711567000	-4.941080000	-0.751055000
H	-6.751974000	-3.625381000	-1.326330000
H	-5.937922000	-4.677394000	-2.480488000
C	-0.344398000	-0.909561000	-6.703141000
H	0.627509000	-0.410037000	-6.638755000
H	-0.190775000	-1.882486000	-7.180719000
H	-0.999658000	-0.309634000	-7.345585000
C	1.830611000	-2.442142000	-3.131481000
C	-4.374822000	-4.681779000	-4.663601000
H	-5.263651000	-4.371573000	-4.119018000

H	-3.884770000	-3.789176000	-5.052552000
H	-4.713889000	-5.272933000	-5.521509000
C	0.300248000	-3.272534000	-4.632639000
H	-0.175962000	-3.925126000	-5.349812000
C	-0.528600000	-5.683713000	0.354813000
C	-4.771791000	-2.097340000	-2.537599000
H	-5.743988000	-1.602323000	-2.431584000
H	-3.994638000	-1.340349000	-2.403577000
H	-4.707054000	-2.484323000	-3.552377000
C	-1.631084000	-5.496074000	-2.006741000
H	-1.072623000	-6.295536000	-2.472190000
C	1.437053000	-3.635051000	-3.840834000
C	0.923283000	-1.416253000	-3.523412000
H	0.940717000	-0.403450000	-3.156167000
C	3.121583000	-5.501946000	-3.116900000
H	3.955397000	-4.842366000	-2.885086000
H	2.588639000	-5.722633000	-2.191245000
H	3.548951000	-6.441376000	-3.484309000
C	-1.187000000	0.309277000	-4.687382000
H	-1.591744000	0.237253000	-3.674475000
H	-0.252182000	0.878594000	-4.649621000
H	-1.892322000	0.878779000	-5.302107000
C	3.199791000	-2.916841000	-0.999509000
H	2.348983000	-2.724172000	-0.340711000
H	3.250938000	-3.986701000	-1.186306000
H	4.118130000	-2.631002000	-0.472490000
C	-4.628931000	-3.200266000	-1.478750000
C	-1.096987000	-7.072568000	0.698531000
H	-2.115341000	-6.983718000	1.089550000
H	-1.132795000	-7.712208000	-0.189055000
H	-0.477989000	-7.567909000	1.456061000
C	-3.411054000	-5.531606000	-3.828483000
C	2.977980000	-0.623982000	-1.846041000
H	3.030221000	0.061147000	-2.696437000
H	2.049468000	-0.431583000	-1.307281000
H	3.816432000	-0.393261000	-1.179925000
C	4.338408000	-2.192143000	-3.144644000
H	4.572649000	-3.211178000	-3.450079000
H	4.231667000	-1.588214000	-4.051511000
H	5.197902000	-1.811244000	-2.580353000
C	3.063431000	-2.091860000	-2.287593000
C	-2.304243000	-1.794792000	-5.429820000
H	-2.178218000	-2.785126000	-5.877114000
H	-2.777540000	-1.914910000	-4.450442000
H	-2.997225000	-1.227423000	-6.061094000
C	-4.157748000	-6.788438000	-3.331677000

H	-3.464845000	-7.470095000	-2.829306000
H	-4.944530000	-6.533628000	-2.619084000
H	-4.615713000	-7.320347000	-4.174394000
C	2.180605000	-4.935315000	-4.184237000
C	-2.328013000	-6.003768000	-4.809381000
H	-1.760323000	-5.156619000	-5.199481000
H	-1.626005000	-6.706677000	-4.358721000
H	-2.798668000	-6.514179000	-5.656479000
C	0.881173000	-5.849233000	-0.215293000
H	0.868321000	-6.459307000	-1.123531000
H	1.319092000	-4.875880000	-0.456449000
H	1.543162000	-6.339689000	0.507561000
C	-0.462804000	-4.843722000	1.632693000
H	-0.066623000	-3.847414000	1.425239000
H	-1.452383000	-4.738447000	2.087017000
H	0.186696000	-5.331220000	2.367960000
C	1.190486000	-6.064156000	-4.504603000
H	0.575587000	-6.297341000	-3.632621000
H	0.525666000	-5.819466000	-5.334216000
H	1.740677000	-6.968772000	-4.784470000
C	2.995660000	-4.653640000	-5.464421000
H	2.332240000	-4.351484000	-6.280220000
H	3.718507000	-3.849479000	-5.310177000
H	3.541569000	-5.551692000	-5.777445000
Y	1.384890000	3.048221000	2.610567000
C	0.983852000	3.148706000	-1.261650000
C	2.169186000	-0.929162000	3.417750000
H	2.667838000	-1.228433000	4.344621000
H	1.171506000	-0.558511000	3.660040000
H	2.032319000	-1.818500000	2.795841000
C	3.910087000	2.618498000	3.205588000
C	3.184121000	1.390642000	3.463674000
C	0.799130000	3.786513000	0.103566000
C	2.354912000	1.636231000	4.588692000
H	1.678499000	0.916351000	5.022652000
C	2.517356000	2.959513000	5.050823000
C	4.305375000	-0.602201000	2.255134000
H	4.877207000	-0.861352000	3.151498000
H	4.066522000	-1.529623000	1.725549000
H	4.946391000	-0.011378000	1.603318000
C	0.384512000	4.153007000	-2.269362000
H	-0.666598000	4.349877000	-2.039907000
H	0.924834000	5.104647000	-2.241734000
H	0.444294000	3.749520000	-3.286393000
C	-0.444701000	4.794911000	1.801820000
C	4.959744000	2.584197000	0.859814000

H	4.698274000	1.546216000	0.658129000
H	4.171614000	3.217064000	0.444577000
H	5.881577000	2.799015000	0.308731000
C	1.573087000	4.815464000	0.686100000
H	2.536411000	5.141534000	0.318979000
C	2.034779000	3.479638000	6.390911000
C	2.188590000	0.468729000	1.392218000
H	2.075916000	-0.387783000	0.724862000
H	1.139710000	0.704184000	1.639666000
H	2.639638000	1.275888000	0.803875000
C	3.458858000	3.561718000	4.181787000
H	3.862651000	4.556023000	4.308737000
C	0.825702000	5.482836000	1.716508000
C	-0.408403000	3.749134000	0.836342000
H	-1.216110000	3.056510000	0.648627000
C	0.849214000	7.313465000	3.572318000
H	-0.231199000	7.383277000	3.680251000
H	1.231739000	6.635509000	4.337089000
H	1.258594000	8.307700000	3.780006000
C	0.206156000	1.837713000	-1.376999000
H	0.522915000	1.101405000	-0.635308000
H	-0.866924000	1.994414000	-1.255457000
H	0.349210000	1.403006000	-2.368277000
C	-1.502339000	4.927158000	4.121731000
H	-1.224125000	3.900364000	4.380336000
H	-0.731716000	5.601654000	4.490804000
H	-2.427919000	5.152875000	4.662765000
C	3.002967000	0.105226000	2.643132000
C	3.153071000	3.189260000	7.410508000
H	3.376463000	2.118677000	7.445335000
H	4.072718000	3.717341000	7.139404000
H	2.852968000	3.512410000	8.413842000
C	5.148442000	2.897160000	2.347036000
C	-2.812207000	4.027186000	2.235236000
H	-3.096089000	4.098106000	1.181498000
H	-2.507250000	2.998233000	2.435735000
H	-3.707363000	4.226829000	2.832558000
C	-2.310813000	6.430439000	2.249304000
H	-1.668484000	7.260376000	2.540651000
H	-2.479390000	6.499073000	1.170094000
H	-3.275022000	6.565816000	2.751207000
C	-1.726058000	5.053292000	2.606722000
C	2.448775000	2.920251000	-1.631736000
H	3.043102000	3.833367000	-1.524642000
H	2.905377000	2.135648000	-1.026796000
H	2.520309000	2.603680000	-2.676941000

C	6.329386000	2.103419000	2.936087000
H	6.498718000	2.401701000	3.975421000
H	6.155082000	1.028421000	2.924555000
H	7.243933000	2.308455000	2.367911000
C	1.298407000	6.869328000	2.178389000
C	5.537005000	4.381861000	2.409533000
H	4.739118000	5.021352000	2.025780000
H	5.786188000	4.702809000	3.424801000
H	6.423432000	4.547141000	1.789155000
C	1.772287000	4.985141000	6.358053000
H	2.636351000	5.542953000	5.983795000
H	0.912115000	5.212267000	5.724645000
H	1.548346000	5.355716000	7.363727000
C	0.755513000	2.760365000	6.834481000
H	-0.047645000	2.881586000	6.101518000
H	0.923294000	1.688700000	6.975747000
H	0.407063000	3.166963000	7.789476000
C	2.832868000	6.939963000	2.203898000
H	3.242718000	6.203998000	2.899952000
H	3.282165000	6.775667000	1.221780000
H	3.148913000	7.933090000	2.538464000
C	0.798780000	7.883301000	1.129134000
H	1.185157000	7.627959000	0.137908000
H	-0.291008000	7.896113000	1.067024000
H	1.139807000	8.893610000	1.382480000

Int3

E= -3485.79655904 Ha

Sum of electronic and thermal Enthalpies= -3483.947948 Ha

Sum of electronic and thermal Free Energies= -3484.174057 Ha

Mo	-1.913203000	-0.195053000	1.520556000
O	-4.685242000	1.150452000	2.405401000
O	-3.577557000	0.986059000	-0.903938000
O	-1.855290000	-1.090371000	-1.911558000
O	-2.716359000	-2.433878000	3.617004000
O	-0.525594000	1.877704000	3.355670000
O	0.089452000	-1.995865000	-0.347452000
C	-3.680207000	0.665358000	2.119350000
C	-2.430743000	-1.607435000	2.865841000
C	-2.761582000	0.377933000	-0.268333000
C	-1.831925000	-0.715908000	-0.678490000
C	-1.105794000	1.069790000	2.664175000
C	-0.738185000	-1.208356000	0.205276000
Y	-0.717147000	-3.029716000	-2.280038000
C	-1.104626000	-1.175050000	-5.406946000
C	-4.711767000	-2.649718000	-0.052326000

H	-4.649673000	-3.338844000	0.795714000
H	-3.941685000	-1.888450000	0.069406000
H	-5.681095000	-2.144007000	-0.003736000
C	-2.678906000	-5.008207000	-2.476315000
C	-3.229373000	-4.091247000	-1.505953000
C	-0.072028000	-1.925247000	-4.589313000
C	-2.336047000	-4.088760000	-0.398204000
H	-2.464888000	-3.494870000	0.493932000
C	-1.282960000	-5.003885000	-0.601224000
C	-5.719472000	-4.435523000	-1.371225000
H	-5.532957000	-5.169728000	-0.580620000
H	-6.676420000	-3.944535000	-1.160657000
H	-5.830817000	-4.977344000	-2.309006000
C	-0.570900000	-1.061920000	-6.845846000
H	0.383477000	-0.525864000	-6.860020000
H	-0.406672000	-2.052153000	-7.282563000
H	-1.281247000	-0.519086000	-7.480445000
C	1.793578000	-2.350675000	-3.259955000
C	-4.299381000	-4.963444000	-4.525748000
H	-5.196779000	-4.683727000	-3.978658000
H	-3.854062000	-4.056753000	-4.936362000
H	-4.617804000	-5.587867000	-5.367956000
C	0.285924000	-3.286453000	-4.721738000
H	-0.168989000	-3.976440000	-5.418219000
C	-0.304368000	-5.506532000	0.442361000
C	-4.845536000	-2.335851000	-2.490650000
H	-5.840200000	-1.895247000	-2.357201000
H	-4.109956000	-1.531270000	-2.423124000
H	-4.803250000	-2.755533000	-3.493655000
C	-1.478866000	-5.536145000	-1.895627000
H	-0.893078000	-6.334748000	-2.328808000
C	1.448927000	-3.576742000	-3.937338000
C	0.835368000	-1.376584000	-3.664662000
H	0.800959000	-0.357998000	-3.312631000
C	3.217241000	-5.349609000	-3.181773000
H	4.025656000	-4.650062000	-2.977752000
H	2.702541000	-5.566137000	-2.244515000
H	3.679456000	-6.280804000	-3.527273000
C	-1.345817000	0.231470000	-4.849427000
H	-1.713715000	0.191441000	-3.821139000
H	-0.425771000	0.825543000	-4.871676000
H	-2.089102000	0.751800000	-5.463014000
C	3.205738000	-2.712706000	-1.134072000
H	2.350892000	-2.548913000	-0.471907000
H	3.312022000	-3.782307000	-1.296447000
H	4.110588000	-2.365714000	-0.621297000

C	-4.591772000	-3.385681000	-1.398600000
C	-0.803673000	-6.882225000	0.919962000
H	-1.810311000	-6.799712000	1.341202000
H	-0.844038000	-7.592435000	0.087957000
H	-0.138785000	-7.290288000	1.690594000
C	-3.289944000	-5.745083000	-3.679841000
C	2.873899000	-0.454132000	-2.027680000
H	2.873869000	0.212599000	-2.894012000
H	1.951640000	-0.291995000	-1.468520000
H	3.717384000	-0.170658000	-1.388670000
C	4.286672000	-1.992569000	-3.308287000
H	4.553652000	-3.007504000	-3.600320000
H	4.147475000	-1.408024000	-4.223386000
H	5.137991000	-1.571548000	-2.760274000
C	3.017826000	-1.925752000	-2.439292000
C	-2.426089000	-1.946454000	-5.421689000
H	-2.285769000	-2.949379000	-5.835768000
H	-2.836841000	-2.041174000	-4.411223000
H	-3.177406000	-1.433112000	-6.032069000
C	-3.968468000	-7.019453000	-3.134603000
H	-3.236433000	-7.649720000	-2.620654000
H	-4.758676000	-6.781233000	-2.420334000
H	-4.408817000	-7.600104000	-3.954171000
C	2.242137000	-4.853387000	-4.254178000
C	-2.194338000	-6.194456000	-4.656951000
H	-1.652403000	-5.333722000	-5.053506000
H	-1.470168000	-6.869173000	-4.197474000
H	-2.647168000	-6.730063000	-5.498167000
C	1.089862000	-5.667219000	-0.165400000
H	1.072750000	-6.360165000	-1.012041000
H	1.472097000	-4.704730000	-0.516260000
H	1.800050000	-6.057924000	0.572324000
C	-0.230310000	-4.560908000	1.645716000
H	0.087334000	-3.560381000	1.343592000
H	-1.201303000	-4.478929000	2.142914000
H	0.483777000	-4.949277000	2.380508000
C	1.294126000	-6.029655000	-4.526284000
H	0.684470000	-6.243795000	-3.645918000
H	0.623534000	-5.844582000	-5.366781000
H	1.876202000	-6.925859000	-4.765838000
C	3.030272000	-4.577407000	-5.552028000
H	2.345231000	-4.323573000	-6.366393000
H	3.723174000	-3.741863000	-5.430081000
H	3.606413000	-5.462327000	-5.847872000
Y	1.339086000	3.038691000	2.619346000
C	0.973519000	3.274219000	-1.250036000

C	1.886454000	-0.998499000	3.282523000
H	2.321784000	-1.346774000	4.224225000
H	0.895408000	-0.584916000	3.485350000
H	1.735793000	-1.863413000	2.630632000
C	3.827681000	2.451681000	3.232248000
C	3.033997000	1.256849000	3.439596000
C	0.816662000	3.882803000	0.132499000
C	2.198030000	1.509586000	4.557572000
H	1.478037000	0.811699000	4.956346000
C	2.422422000	2.807288000	5.066242000
C	4.090907000	-0.760443000	2.228430000
H	4.611344000	-1.061814000	3.142768000
H	3.829813000	-1.665444000	1.672357000
H	4.786095000	-0.190753000	1.614235000
C	0.405061000	4.325322000	-2.227437000
H	-0.641811000	4.542309000	-1.996641000
H	0.969808000	5.260997000	-2.165978000
H	0.460114000	3.953645000	-3.256712000
C	-0.369347000	4.922769000	1.853792000
C	4.942993000	2.420034000	0.909915000
H	4.652252000	1.396835000	0.675189000
H	4.192846000	3.091795000	0.483887000
H	5.889390000	2.612887000	0.393574000
C	1.651626000	4.842677000	0.748265000
H	2.634463000	5.119005000	0.392292000
C	1.944803000	3.311215000	6.414486000
C	2.079341000	0.455200000	1.303955000
H	1.921963000	-0.376914000	0.614228000
H	1.042804000	0.768188000	1.520167000
H	2.609871000	1.239729000	0.755457000
C	3.409437000	3.385559000	4.232403000
H	3.861760000	4.352928000	4.396983000
C	0.943309000	5.528662000	1.794214000
C	-0.395302000	3.903532000	0.859284000
H	-1.244164000	3.267214000	0.652310000
C	1.053034000	7.324775000	3.684300000
H	-0.021807000	7.464026000	3.779264000
H	1.380244000	6.611610000	4.443206000
H	1.523151000	8.286696000	3.914407000
C	0.156360000	1.989968000	-1.400247000
H	0.468391000	1.216950000	-0.695442000
H	-0.909521000	2.169731000	-1.244355000
H	0.267383000	1.588423000	-2.411081000
C	-1.427783000	5.082215000	4.171739000
H	-1.233334000	4.032904000	4.416455000
H	-0.606621000	5.686219000	4.552552000

H	-2.334161000	5.373059000	4.714064000
C	2.811904000	0.007729000	2.576731000
C	3.010676000	2.895609000	7.446904000
H	3.148011000	1.810091000	7.446057000
H	3.975668000	3.357949000	7.216605000
H	2.711936000	3.204995000	8.454888000
C	5.099356000	2.690685000	2.409980000
C	-2.777298000	4.290551000	2.270124000
H	-3.058539000	4.394181000	1.218418000
H	-2.521935000	3.246205000	2.454293000
H	-3.660274000	4.523286000	2.872716000
C	-2.136375000	6.660098000	2.317666000
H	-1.450961000	7.447560000	2.628046000
H	-2.290023000	6.754916000	1.238203000
H	-3.096355000	6.842454000	2.812707000
C	-1.635753000	5.246139000	2.658229000
C	2.431460000	3.013716000	-1.626213000
H	3.047547000	3.911148000	-1.509927000
H	2.869692000	2.212623000	-1.029719000
H	2.493655000	2.706585000	-2.674488000
C	6.226813000	1.831381000	3.011967000
H	6.373034000	2.091771000	4.064872000
H	6.009756000	0.765351000	2.961274000
H	7.167435000	2.015078000	2.480248000
C	1.493811000	6.875468000	2.288949000
C	5.550916000	4.154333000	2.519049000
H	4.784139000	4.835586000	2.145506000
H	5.800349000	4.436100000	3.545855000
H	6.451251000	4.299405000	1.914078000
C	1.800944000	4.833119000	6.435291000
H	2.718047000	5.335283000	6.111983000
H	0.983594000	5.152160000	5.785147000
H	1.571727000	5.181077000	7.447756000
C	0.603380000	2.676845000	6.800628000
H	-0.173454000	2.896286000	6.062447000
H	0.682472000	1.589689000	6.892203000
H	0.269049000	3.064192000	7.768592000
C	3.028890000	6.859186000	2.337801000
H	3.387627000	6.121878000	3.060179000
H	3.484772000	6.646733000	1.368212000
H	3.394077000	7.841502000	2.653919000
C	1.069128000	7.933185000	1.249411000
H	1.467445000	7.679310000	0.262462000
H	-0.016907000	7.998916000	1.161214000
H	1.451442000	8.920872000	1.531407000

Int4

E= -4854.69010005 Ha

Sum of electronic and thermal Enthalpies= -4851.947891 Ha

Sum of electronic and thermal Free Energies= -4852.263329 Ha

Mo	-0.420235000	0.631069000	0.926981000
O	0.975325000	3.057767000	-0.409225000
O	-2.354593000	0.964991000	-1.669000000
O	-1.189869000	-1.921937000	-2.062688000
O	-2.562132000	2.165321000	2.741408000
O	2.089402000	0.836536000	2.772301000
O	1.011587000	-1.979293000	-0.371761000
C	0.430025000	2.158672000	0.104824000
C	-1.769287000	1.653322000	2.060555000
C	-1.518159000	0.330499000	-0.941320000
C	-0.904945000	-0.955191000	-1.254669000
C	1.063445000	0.675884000	2.133155000
C	0.199227000	-0.990873000	-0.302621000
Y	0.270519000	-3.718805000	-1.599686000
C	-0.180834000	-4.027475000	-5.222231000
C	-3.686986000	-2.965033000	0.082993000
H	-3.573584000	-2.981501000	1.170929000
H	-3.039577000	-2.185613000	-0.324898000
H	-4.723926000	-2.686213000	-0.129800000
C	-1.170352000	-5.900735000	-0.703232000
C	-1.940277000	-4.747901000	-0.299462000
C	0.889356000	-3.898198000	-4.159168000
C	-1.148799000	-4.033055000	0.645035000
H	-1.438378000	-3.104664000	1.116025000
C	0.064891000	-4.705816000	0.882293000
C	-4.328256000	-5.340648000	0.152735000
H	-4.085266000	-5.398172000	1.218600000
H	-5.371876000	-5.018401000	0.056078000
H	-4.251449000	-6.347623000	-0.258036000
C	0.467161000	-4.609060000	-6.490357000
H	1.285565000	-3.967464000	-6.831422000
H	0.878683000	-5.605017000	-6.298283000
H	-0.268626000	-4.691393000	-7.299312000
C	2.623358000	-2.996610000	-2.887319000
C	-2.683541000	-7.326100000	-2.321848000
H	-3.639415000	-7.021259000	-1.898141000
H	-2.483158000	-6.717133000	-3.204211000
H	-2.799394000	-8.362001000	-2.659828000
C	1.597952000	-4.950787000	-3.543334000
H	1.425435000	-6.001437000	-3.727856000
C	1.060558000	-4.448924000	1.994722000
C	-3.740958000	-4.211151000	-2.034407000

H	-4.804059000	-3.974711000	-2.152925000
H	-3.163071000	-3.399729000	-2.482931000
H	-3.546501000	-5.122135000	-2.591475000
C	0.065237000	-5.820242000	0.017448000
H	0.822769000	-6.590932000	0.009395000
C	2.693710000	-4.433611000	-2.779418000
C	1.501422000	-2.707509000	-3.716436000
H	1.171866000	-1.714296000	-3.978222000
C	4.823231000	-4.973008000	-1.348094000
H	5.407609000	-4.111309000	-1.668907000
H	4.341140000	-4.736593000	-0.398509000
H	5.529498000	-5.790833000	-1.165797000
C	-0.767684000	-2.654853000	-5.543311000
H	-1.172796000	-2.186892000	-4.641913000
H	0.002200000	-1.993878000	-5.950367000
H	-1.567603000	-2.735861000	-6.285941000
C	3.983207000	-1.803645000	-1.019067000
H	3.111111000	-1.586967000	-0.401119000
H	4.433134000	-2.725551000	-0.663686000
H	4.717906000	-1.001512000	-0.881086000
C	-3.393756000	-4.337712000	-0.547879000
C	0.845578000	-5.539793000	3.060575000
H	-0.181345000	-5.512023000	3.438370000
H	1.024085000	-6.536208000	2.644149000
H	1.529403000	-5.392913000	3.905563000
C	-1.531603000	-7.268116000	-1.313733000
C	2.954919000	-0.495971000	-2.811929000
H	2.782213000	-0.358200000	-3.883039000
H	2.009396000	-0.359885000	-2.281275000
H	3.635321000	0.297358000	-2.485056000
C	4.845334000	-1.979932000	-3.381520000
H	5.418017000	-2.886163000	-3.175062000
H	4.568491000	-1.995097000	-4.440768000
H	5.506661000	-1.121757000	-3.211384000
C	3.587024000	-1.864238000	-2.500416000
C	-1.295650000	-4.964103000	-4.754666000
H	-0.900521000	-5.956928000	-4.522846000
H	-1.785427000	-4.574003000	-3.857124000
H	-2.068186000	-5.079205000	-5.523738000
C	-1.893049000	-8.189837000	-0.128854000
H	-1.052249000	-8.271623000	0.565818000
H	-2.746967000	-7.796787000	0.428970000
H	-2.147743000	-9.195211000	-0.485278000
C	3.811379000	-5.421381000	-2.404417000
C	-0.319474000	-7.880823000	-2.034863000
H	-0.023084000	-7.267424000	-2.889247000

H	0.548904000	-7.993019000	-1.383921000
H	-0.577932000	-8.877807000	-2.408055000
C	2.491153000	-4.548132000	1.463383000
H	2.658755000	-5.505640000	0.962513000
H	2.691803000	-3.742128000	0.752390000
H	3.217067000	-4.472928000	2.278559000
C	0.847527000	-3.078790000	2.644360000
H	1.007064000	-2.269155000	1.929668000
H	-0.164080000	-2.987966000	3.053873000
H	1.550167000	-2.955011000	3.476536000
C	3.214028000	-6.741955000	-1.892948000
H	2.614075000	-6.570716000	-0.996663000
H	2.579123000	-7.231091000	-2.633777000
H	4.017906000	-7.441535000	-1.639485000
C	4.586470000	-5.723348000	-3.705091000
H	3.920106000	-6.146900000	-4.462100000
H	5.026578000	-4.813581000	-4.121983000
H	5.394681000	-6.439926000	-3.514455000
Y	3.617929000	1.856784000	4.052360000
C	5.121360000	4.169528000	1.373721000
C	4.472654000	-1.981813000	2.510608000
H	4.394725000	-2.794405000	3.237951000
H	3.474153000	-1.578237000	2.328836000
H	4.821675000	-2.406744000	1.567334000
C	5.625637000	0.591163000	5.261599000
C	5.022019000	-0.353084000	4.345703000
C	4.302773000	4.003505000	2.644697000
C	3.723555000	-0.632993000	4.840998000
H	3.014289000	-1.293648000	4.367145000
C	3.476895000	0.083116000	6.032271000
C	6.838804000	-1.596124000	2.990329000
H	6.849719000	-2.421383000	3.708983000
H	7.030885000	-2.016686000	1.997569000
H	7.666232000	-0.930313000	3.227085000
C	4.943396000	5.644653000	0.955726000
H	3.889342000	5.868645000	0.768352000
H	5.298854000	6.316893000	1.743095000
H	5.508687000	5.853656000	0.040342000
C	2.394008000	4.238686000	3.966914000
C	7.768916000	1.550755000	4.200145000
H	7.758575000	0.877174000	3.345597000
H	7.271853000	2.478406000	3.904483000
H	8.816460000	1.788918000	4.414109000
C	4.700542000	4.250223000	3.981631000
H	5.720798000	4.406673000	4.303729000
C	2.319444000	-0.213482000	6.968780000

C	5.413753000	0.232316000	1.964869000
H	5.811478000	-0.067373000	0.989712000
H	4.368661000	0.517248000	1.772023000
H	5.982395000	1.107577000	2.290754000
C	4.643371000	0.850691000	6.273207000
H	4.807096000	1.466805000	7.146316000
C	3.548680000	4.419474000	4.820602000
C	2.889755000	3.954759000	2.664522000
H	2.273752000	3.781424000	1.794742000
C	2.726603000	4.509857000	7.265432000
H	1.677620000	4.533961000	6.980578000
H	2.990221000	3.473133000	7.487605000
H	2.828356000	5.081622000	8.194272000
C	4.603623000	3.280285000	0.236247000
H	4.719987000	2.218861000	0.464817000
H	3.547935000	3.461638000	0.021169000
H	5.166784000	3.484150000	-0.680736000
C	0.395900000	3.076837000	4.979029000
H	0.543053000	2.178657000	4.367825000
H	0.910232000	2.951054000	5.934560000
H	-0.678783000	3.132034000	5.183280000
C	5.464788000	-0.913345000	2.987968000
C	2.700578000	-1.485736000	7.750789000
H	2.874020000	-2.322549000	7.067693000
H	3.616178000	-1.326261000	8.329142000
H	1.898637000	-1.765843000	8.443192000
C	7.096830000	0.977738000	5.452654000
C	0.138139000	4.325358000	2.867062000
H	0.454054000	5.166426000	2.243382000
H	0.283953000	3.398821000	2.311211000
H	-0.935743000	4.419565000	3.045563000
C	0.427651000	5.606402000	4.917964000
H	0.796539000	5.717458000	5.935554000
H	0.741736000	6.483119000	4.343019000
H	-0.666294000	5.617242000	4.966298000
C	0.878442000	4.318180000	4.212313000
C	6.612982000	3.912503000	1.591454000
H	7.015449000	4.525495000	2.404315000
H	6.817155000	2.864720000	1.819818000
H	7.169782000	4.163482000	0.682854000
C	7.843140000	-0.267033000	5.972337000
H	7.410799000	-0.586152000	6.925916000
H	7.783698000	-1.109529000	5.285553000
H	8.901111000	-0.032909000	6.138246000
C	3.676599000	5.072114000	6.202772000
C	7.260957000	2.061124000	6.524367000

H	6.792550000	2.993479000	6.210049000
H	6.847827000	1.763293000	7.491851000
H	8.326954000	2.259674000	6.674390000
C	2.073813000	0.918303000	7.965659000
H	2.976254000	1.161295000	8.535733000
H	1.731775000	1.824250000	7.461280000
H	1.298315000	0.626932000	8.681306000
C	1.029146000	-0.483982000	6.182776000
H	0.729329000	0.382764000	5.587995000
H	1.136498000	-1.332313000	5.501612000
H	0.210331000	-0.716095000	6.871834000
C	5.079177000	4.886634000	6.793784000
H	5.283640000	3.833376000	6.986467000
H	5.868038000	5.290554000	6.153832000
H	5.138277000	5.414954000	7.750779000
C	3.482197000	6.590721000	6.016305000
H	4.249974000	6.980979000	5.340683000
H	2.512032000	6.841079000	5.591623000
H	3.579585000	7.106335000	6.978641000
Y	-3.764251000	2.034526000	-2.895719000
C	-6.365489000	1.485209000	-0.130059000
C	-4.517877000	-2.180746000	-5.010694000
H	-4.680510000	-2.115236000	-6.091374000
H	-3.450616000	-2.326245000	-4.839240000
H	-5.043865000	-3.065638000	-4.636250000
C	-4.594527000	1.540322000	-5.477540000
C	-4.249722000	0.303002000	-4.824867000
C	-5.563933000	2.475462000	-0.955147000
C	-2.842586000	0.327700000	-4.598824000
H	-2.287223000	-0.456267000	-4.101961000
C	-2.293262000	1.542708000	-5.054764000
C	-6.548377000	-0.863668000	-4.457863000
H	-6.870069000	-0.865982000	-5.499691000
H	-6.976328000	-1.753553000	-3.983682000
H	-6.973096000	0.011391000	-3.964004000
C	-7.045832000	2.298463000	0.989426000
H	-6.297227000	2.801145000	1.608695000
H	-7.704499000	3.063538000	0.565982000
H	-7.644156000	1.643067000	1.633231000
C	-4.023928000	4.175669000	-1.348769000
C	-7.169063000	1.969231000	-5.660471000
H	-7.723454000	1.034837000	-5.741876000
H	-7.065532000	2.201351000	-4.598942000
H	-7.790971000	2.747202000	-6.114253000
C	-6.001434000	3.240147000	-2.065099000
H	-6.945981000	3.106011000	-2.572603000

C	-0.812887000	1.821229000	-5.241573000
C	-4.743954000	-1.006748000	-2.804626000
H	-5.316316000	-1.812888000	-2.338219000
H	-3.688735000	-1.163294000	-2.575691000
H	-5.085664000	-0.092004000	-2.291256000
C	-3.383088000	2.298605000	-5.552604000
H	-3.287457000	3.252515000	-6.049870000
C	-5.086377000	4.308435000	-2.321209000
C	-4.345274000	3.052877000	-0.540675000
H	-3.741381000	2.679947000	0.271620000
C	-4.350314000	5.840994000	-4.242931000
H	-3.448611000	6.213845000	-3.758475000
H	-4.069077000	4.968506000	-4.840371000
H	-4.699396000	6.622611000	-4.926944000
C	-5.455762000	0.434352000	0.522515000
H	-4.903876000	-0.152326000	-0.214788000
H	-4.721767000	0.900291000	1.185088000
H	-6.052844000	-0.258150000	1.125662000
C	-1.755054000	4.414721000	-2.317412000
H	-1.547418000	3.337921000	-2.218110000
H	-2.186183000	4.622166000	-3.300898000
H	-0.769637000	4.891598000	-2.277984000
C	-5.029762000	-0.912686000	-4.311766000
C	-0.384257000	1.053441000	-6.507047000
H	-0.590149000	-0.012971000	-6.398371000
H	-0.934467000	1.414341000	-7.382129000
H	0.688114000	1.184640000	-6.693940000
C	-5.794395000	1.895340000	-6.367157000
C	-2.020677000	4.463464000	0.137908000
H	-2.649586000	4.720552000	0.995018000
H	-1.819743000	3.392023000	0.165652000
H	-1.059456000	4.971516000	0.248742000
C	-2.736916000	6.418180000	-1.147201000
H	-3.065508000	6.867980000	-2.082772000
H	-3.404666000	6.755862000	-0.348922000
H	-1.736875000	6.810981000	-0.933466000
C	-2.674876000	4.886518000	-1.181655000
C	-7.458658000	0.800155000	-0.955372000
H	-8.143603000	1.532716000	-1.394379000
H	-7.045868000	0.196957000	-1.766474000
H	-8.050651000	0.136405000	-0.316465000
C	-5.846933000	0.840216000	-7.490678000
H	-4.912438000	0.843582000	-8.060278000
H	-5.987753000	-0.167893000	-7.097282000
H	-6.671744000	1.058150000	-8.179294000
C	-5.458205000	5.469824000	-3.247993000

C	-5.552029000	3.242513000	-7.073871000
H	-5.434451000	4.070598000	-6.372273000
H	-4.663570000	3.204926000	-7.710240000
H	-6.408516000	3.470608000	-7.715919000
C	-0.522769000	3.306671000	-5.472472000
H	-1.073741000	3.693480000	-6.335770000
H	-0.777671000	3.914436000	-4.603229000
H	0.544541000	3.447977000	-5.672487000
C	0.007900000	1.312716000	-4.050544000
H	-0.233975000	1.846449000	-3.128421000
H	-0.168265000	0.249614000	-3.865581000
H	1.076719000	1.447425000	-4.243119000
C	-6.699294000	5.118531000	-4.078679000
H	-6.546085000	4.218076000	-4.669544000
H	-7.580265000	4.968867000	-3.447831000
H	-6.923944000	5.939478000	-4.767297000
C	-5.859937000	6.685358000	-2.390144000
H	-6.692711000	6.413982000	-1.733781000
H	-5.049657000	7.048023000	-1.761972000
H	-6.188190000	7.509280000	-3.034828000

TS3

E= -4854.66563175 Ha

Sum of electronic and thermal Enthalpies= -4851.923566 Ha

Sum of electronic and thermal Free Energies= -4852.232249 Ha

Imag_freq=-131.9 cm-1

Mo	-2.094761000	0.699212000	1.154376000
O	-1.965043000	3.510543000	-0.109470000
O	-3.750117000	2.007881000	-1.651768000
O	-2.443779000	-0.632737000	-2.455092000
O	-4.703363000	1.385489000	2.833564000
O	-0.112405000	1.926130000	3.187448000
O	-0.296364000	-1.086546000	-0.827284000
C	-2.115805000	2.358152000	0.203468000
C	-3.744882000	1.103215000	2.239929000
C	-2.823125000	1.360697000	-1.067706000
C	-2.144988000	0.187174000	-1.467299000
C	-0.911679000	1.367190000	2.458304000
C	-1.091560000	-0.141149000	-0.517116000
Y	-1.244760000	-2.583921000	-2.240204000
C	-1.591833000	-2.674791000	-5.896641000
C	-5.181005000	-1.651639000	-0.559559000
H	-5.108616000	-1.739693000	0.528542000
H	-4.455835000	-0.909362000	-0.900720000
H	-6.182246000	-1.272763000	-0.786173000
C	-2.863750000	-4.704149000	-1.490624000

C	-3.567430000	-3.535112000	-1.021398000
C	-0.551844000	-2.699987000	-4.795981000
C	-2.756514000	-2.944944000	-0.013530000
H	-3.020297000	-2.048830000	0.531442000
C	-1.594437000	-3.707780000	0.202345000
C	-6.011331000	-3.967938000	-0.657641000
H	-5.807084000	-4.110137000	0.408464000
H	-7.021510000	-3.553991000	-0.759542000
H	-6.006466000	-4.950996000	-1.129349000
C	-1.032422000	-3.429892000	-7.113927000
H	-0.096833000	-2.974204000	-7.452597000
H	-0.826796000	-4.476217000	-6.867590000
H	-1.748563000	-3.408647000	-7.943974000
C	1.217728000	-2.055963000	-3.423673000
C	-4.448599000	-5.909127000	-3.212441000
H	-5.385666000	-5.547762000	-2.792021000
H	-4.176425000	-5.269880000	-4.053700000
H	-4.641643000	-6.910667000	-3.612749000
C	0.019516000	-3.852388000	-4.215372000
H	-0.251981000	-4.867737000	-4.468049000
C	-0.636830000	-3.597658000	1.372379000
C	-5.266226000	-2.748995000	-2.749867000
H	-6.303542000	-2.424221000	-2.885185000
H	-4.613274000	-1.951172000	-3.109995000
H	-5.112679000	-3.625609000	-3.371123000
C	-1.641298000	-4.755116000	-0.741396000
H	-0.935039000	-5.570388000	-0.784922000
C	1.137480000	-3.495742000	-3.396272000
C	0.159433000	-1.605148000	-4.267171000
H	-0.060164000	-0.570703000	-4.481361000
C	3.183664000	-4.317971000	-1.977590000
H	3.866591000	-3.516205000	-2.257698000
H	2.713940000	-4.066713000	-1.024972000
H	3.791056000	-5.216360000	-1.820612000
C	-1.898396000	-1.235345000	-6.307291000
H	-2.240437000	-0.641868000	-5.455422000
H	-1.006795000	-0.753427000	-6.719421000
H	-2.680118000	-1.210375000	-7.072705000
C	2.688376000	-1.125400000	-1.486385000
H	1.834782000	-0.854150000	-0.864423000
H	3.042652000	-2.101543000	-1.171084000
H	3.499594000	-0.409058000	-1.308713000
C	-4.979797000	-3.002868000	-1.268537000
C	-0.920676000	-4.788871000	2.306904000
H	-1.962827000	-4.776435000	2.641509000
H	-0.744675000	-5.738789000	1.792632000

H	-0.272289000	-4.751535000	3.190623000
C	-3.322263000	-6.003922000	-2.178686000
C	1.795488000	0.383822000	-3.175074000
H	1.620558000	0.618617000	-4.228552000
H	0.872890000	0.561923000	-2.618740000
H	2.553697000	1.083525000	-2.805915000
C	3.531433000	-1.241221000	-3.860424000
H	4.003943000	-2.214912000	-3.718786000
H	3.254908000	-1.156161000	-4.916249000
H	4.279223000	-0.470732000	-3.636811000
C	2.291149000	-1.057639000	-2.966881000
C	-2.869730000	-3.370573000	-5.425680000
H	-2.657387000	-4.407466000	-5.151884000
H	-3.295961000	-2.868286000	-4.551571000
H	-3.640543000	-3.375428000	-6.204938000
C	-3.794354000	-6.944833000	-1.048516000
H	-2.979869000	-7.141835000	-0.345525000
H	-4.617810000	-6.499312000	-0.484809000
H	-4.136253000	-7.902448000	-1.459629000
C	2.141386000	-4.611038000	-3.058744000
C	-2.152552000	-6.690318000	-2.901013000
H	-1.792384000	-6.078860000	-3.732268000
H	-1.309609000	-6.893466000	-2.239235000
H	-2.485966000	-7.650550000	-3.309406000
C	0.818844000	-3.665889000	0.903208000
H	1.005398000	-4.598085000	0.361689000
H	1.044694000	-2.826380000	0.242430000
H	1.507729000	-3.631456000	1.754825000
C	-0.866271000	-2.304826000	2.161397000
H	-0.632504000	-1.428282000	1.545292000
H	-1.898876000	-2.238766000	2.523239000
H	-0.211005000	-2.269591000	3.037772000
C	1.417665000	-5.888201000	-2.606492000
H	0.900018000	-5.725010000	-1.658219000
H	0.686703000	-6.240366000	-3.335471000
H	2.146725000	-6.692497000	-2.458944000
C	2.893624000	-4.932117000	-4.368635000
H	2.196354000	-5.264401000	-5.143074000
H	3.415863000	-4.050288000	-4.749219000
H	3.632727000	-5.725472000	-4.203859000
Y	1.648958000	3.286993000	3.487908000
C	1.754499000	5.327908000	0.166455000
C	2.363502000	-0.575188000	2.247834000
H	2.697616000	-1.302319000	2.994174000
H	1.331613000	-0.293694000	2.466683000
H	2.353891000	-1.068572000	1.274789000

C	4.101136000	2.486865000	4.039710000
C	3.357588000	1.339806000	3.558850000
C	1.402047000	5.216603000	1.642811000
C	2.402603000	1.023728000	4.559394000
H	1.685992000	0.220124000	4.491641000
C	2.502297000	1.918345000	5.647030000
C	4.653710000	0.118776000	1.695907000
H	5.087694000	-0.562852000	2.433899000
H	4.507688000	-0.441418000	0.767751000
H	5.379551000	0.904709000	1.492780000
C	1.156932000	6.677084000	-0.287284000
H	0.075775000	6.689752000	-0.122805000
H	1.595915000	7.508190000	0.274202000
H	1.347545000	6.839733000	-1.354445000
C	-0.009113000	5.329139000	3.497357000
C	5.466610000	3.541536000	2.131143000
H	5.247718000	2.748868000	1.416777000
H	4.736642000	4.341920000	1.985306000
H	6.454140000	3.942711000	1.878880000
C	2.120486000	5.761896000	2.732112000
H	3.126034000	6.153912000	2.669844000
C	1.885562000	1.690766000	7.014474000
C	2.681878000	1.627565000	1.184196000
H	2.716281000	1.241198000	0.161412000
H	1.603973000	1.766918000	1.371258000
H	3.191224000	2.597916000	1.174789000
C	3.532186000	2.828484000	5.307217000
H	3.907625000	3.602748000	5.960468000
C	1.274514000	5.875919000	3.885883000
C	0.110443000	4.915966000	2.139492000
H	-0.684797000	4.477386000	1.550508000
C	1.156646000	6.491076000	6.418599000
H	0.070650000	6.501475000	6.482311000
H	1.514375000	5.513999000	6.750776000
H	1.528301000	7.235146000	7.131318000
C	1.122410000	4.219353000	-0.678931000
H	1.507712000	3.230981000	-0.419150000
H	0.038362000	4.190651000	-0.569540000
H	1.347796000	4.391227000	-1.736705000
C	-1.175330000	4.244798000	5.471125000
H	-0.936748000	3.227780000	5.139118000
H	-0.402986000	4.580052000	6.160597000
H	-2.115477000	4.175999000	6.029838000
C	3.300909000	0.641593000	2.193352000
C	2.855618000	0.771753000	7.783591000
H	2.999232000	-0.172923000	7.250577000

H	3.834642000	1.248405000	7.894921000
H	2.464368000	0.548768000	8.782829000
C	5.442572000	3.077550000	3.590137000
C	-2.396218000	4.517720000	3.355339000
H	-2.625399000	5.143175000	2.493231000
H	-2.096825000	3.534750000	2.990764000
H	-3.319335000	4.385939000	3.928217000
C	-1.907948000	6.535583000	4.661649000
H	-1.286281000	7.073080000	5.376481000
H	-2.028486000	7.169538000	3.777596000
H	-2.895118000	6.400472000	5.116709000
C	-1.334618000	5.168743000	4.253968000
C	3.262109000	5.358170000	-0.089267000
H	3.769500000	6.117523000	0.514273000
H	3.724852000	4.390005000	0.114103000
H	3.451214000	5.594770000	-1.141438000
C	6.537631000	2.027597000	3.856505000
H	6.553926000	1.767131000	4.919384000
H	6.378927000	1.109313000	3.293250000
H	7.521097000	2.429766000	3.587117000
C	1.695600000	6.819508000	5.023760000
C	5.806179000	4.315054000	4.422176000
H	5.063952000	5.105949000	4.302687000
H	5.906550000	4.085427000	5.486646000
H	6.769664000	4.707259000	4.081590000
C	1.720393000	2.993531000	7.796720000
H	2.660794000	3.548778000	7.869688000
H	0.974454000	3.637231000	7.327027000
H	1.380876000	2.783230000	8.816079000
C	0.524465000	0.993133000	6.906223000
H	-0.191515000	1.590291000	6.335106000
H	0.607985000	0.015540000	6.422726000
H	0.105843000	0.830281000	7.904757000
C	3.222685000	6.844387000	5.182253000
H	3.599034000	5.855960000	5.455326000
H	3.739258000	7.178346000	4.279328000
H	3.495376000	7.539435000	5.982805000
C	1.248028000	8.236937000	4.612842000
H	1.714218000	8.519580000	3.664055000
H	0.166298000	8.298581000	4.482774000
H	1.545451000	8.967420000	5.374118000
Y	-4.661171000	3.489867000	-2.995099000
C	-7.582960000	2.979494000	-0.656882000
C	-5.370237000	-0.522933000	-5.550849000
H	-5.309835000	-0.377960000	-6.634128000
H	-4.372160000	-0.768834000	-5.189954000

H	-6.021759000	-1.380976000	-5.353861000
C	-5.099146000	3.198989000	-5.745091000
C	-4.959169000	1.910405000	-5.119470000
C	-6.674927000	3.987756000	-1.338697000
C	-3.616317000	1.812962000	-4.644855000
H	-3.211038000	0.957183000	-4.116628000
C	-2.919675000	3.011663000	-4.917901000
C	-7.368120000	0.922434000	-5.281150000
H	-7.489536000	1.016264000	-6.359974000
H	-7.934238000	0.038979000	-4.967037000
H	-7.821465000	1.791903000	-4.801855000
C	-8.316127000	3.739604000	0.466898000
H	-7.603164000	4.133864000	1.196790000
H	-8.884330000	4.581594000	0.058329000
H	-9.010876000	3.072953000	0.990592000
C	-5.109547000	5.705663000	-1.503315000
C	-7.556333000	3.743433000	-6.521899000
H	-8.124136000	2.892574000	-6.899820000
H	-7.725310000	3.798420000	-5.446313000
H	-7.993127000	4.639940000	-6.971117000
C	-6.941049000	4.740288000	-2.508132000
H	-7.791892000	4.586332000	-3.156394000
C	-1.422014000	3.223645000	-4.789594000
C	-5.930875000	0.525913000	-3.343531000
H	-6.652411000	-0.241883000	-3.051849000
H	-4.956676000	0.257868000	-2.935871000
H	-6.276414000	1.444583000	-2.843337000
C	-3.852790000	3.872492000	-5.545949000
H	-3.607530000	4.842158000	-5.952813000
C	-6.007381000	5.819456000	-2.626162000
C	-5.529197000	4.571261000	-0.752233000
H	-5.051593000	4.214111000	0.148779000
C	-4.997609000	7.380231000	-4.399583000
H	-4.191935000	7.760823000	-3.774433000
H	-4.606276000	6.522429000	-4.953940000
H	-5.249559000	8.165110000	-5.121337000
C	-6.781581000	1.836140000	-0.021568000
H	-6.224770000	1.256541000	-0.759559000
H	-6.058241000	2.215101000	0.703266000
H	-7.453390000	1.154449000	0.511190000
C	-2.712676000	6.048280000	-2.030722000
H	-2.487234000	4.984883000	-1.869534000
H	-2.965056000	6.222782000	-3.080322000
H	-1.777342000	6.579906000	-1.820784000
C	-5.911638000	0.736710000	-4.862371000
C	-0.750299000	2.400932000	-5.903610000

H	-0.977984000	1.339544000	-5.787521000
H	-1.106011000	2.715542000	-6.890116000
H	0.338307000	2.527340000	-5.870467000
C	-6.049363000	3.635615000	-6.872628000
C	-3.463631000	6.155918000	0.341342000
H	-4.260517000	6.440497000	1.035917000
H	-3.237980000	5.099890000	0.464280000
H	-2.559017000	6.710828000	0.613772000
C	-3.992995000	8.021614000	-1.127750000
H	-4.172252000	8.440221000	-2.115392000
H	-4.801926000	8.343573000	-0.465095000
H	-3.063024000	8.464349000	-0.754321000
C	-3.853937000	6.493665000	-1.103579000
C	-8.638870000	2.425031000	-1.618491000
H	-9.280479000	3.223398000	-2.005476000
H	-8.195009000	1.906291000	-2.471296000
H	-9.280776000	1.709765000	-1.093975000
C	-5.861277000	2.605979000	-8.007724000
H	-4.812238000	2.567800000	-8.317038000
H	-6.150000000	1.600524000	-7.696308000
H	-6.468743000	2.883403000	-8.877191000
C	-6.247486000	6.969852000	-3.607574000
C	-5.600364000	4.985734000	-7.464098000
H	-5.628582000	5.795975000	-6.733611000
H	-4.587966000	4.926778000	-7.873174000
H	-6.271698000	5.257494000	-8.284774000
C	-1.034550000	4.692652000	-4.977427000
H	-1.335632000	5.066041000	-5.961568000
H	-1.484215000	5.333225000	-4.216176000
H	0.052632000	4.800092000	-4.902028000
C	-0.908055000	2.721257000	-3.438965000
H	-1.335700000	3.274334000	-2.597817000
H	-1.147795000	1.665322000	-3.297654000
H	0.179298000	2.826828000	-3.381467000
C	-7.298400000	6.566259000	-4.645900000
H	-6.994915000	5.666803000	-5.173740000
H	-8.276516000	6.386088000	-4.190736000
H	-7.421091000	7.368166000	-5.381087000
C	-6.850194000	8.168009000	-2.847963000
H	-7.782028000	7.862363000	-2.361926000
H	-6.191973000	8.565528000	-2.079452000
H	-7.082236000	8.977406000	-3.550379000

Int5

E= -4854.68590772 Ha
Sum of electronic and thermal Enthalpies= -4851.941907 Ha
Sum of electronic and thermal Free Energies= -4852.250629 Ha

Mo	-0.526465000	-1.041667000	1.941714000
O	-0.815002000	1.826746000	0.818618000
O	-2.135804000	0.691062000	-1.370455000
O	-0.902599000	-1.995340000	-1.967208000
O	-2.923270000	0.081777000	3.721619000
O	1.308859000	0.448836000	3.877747000
O	1.170800000	-2.429005000	-0.291679000
C	-0.830515000	0.605111000	0.666383000
C	-2.049806000	-0.383216000	3.115913000
C	-1.314876000	0.035577000	-0.638073000
C	-0.714195000	-1.216387000	-0.934502000
C	0.559208000	-0.175274000	3.128222000
C	0.310739000	-1.538753000	0.046779000
Y	0.287005000	-3.954340000	-1.701585000
C	0.025103000	-4.087660000	-5.364267000
C	-3.751114000	-3.049198000	-0.253851000
H	-3.745271000	-3.139533000	0.836829000
H	-3.014799000	-2.298561000	-0.548406000
H	-4.739971000	-2.680405000	-0.541027000
C	-1.352119000	-6.086244000	-0.998766000
C	-2.095282000	-4.918167000	-0.595153000
C	1.025628000	-4.105191000	-4.228408000
C	-1.349952000	-4.296992000	0.444003000
H	-1.663358000	-3.396703000	0.955849000
C	-0.186233000	-5.032228000	0.732324000
C	-4.561312000	-5.365708000	-0.410343000
H	-4.435409000	-5.503518000	0.668458000
H	-5.562179000	-4.954613000	-0.586771000
H	-4.520722000	-6.350313000	-0.876357000
C	0.739360000	-4.576719000	-6.636603000
H	1.602488000	-3.942169000	-6.860281000
H	1.099272000	-5.602796000	-6.511657000
H	0.059600000	-4.552123000	-7.496709000
C	2.760149000	-3.437296000	-2.821093000
C	-2.811517000	-7.339172000	-2.794225000
H	-3.771901000	-6.952824000	-2.457989000
H	-2.469507000	-6.736589000	-3.636462000
H	-2.987915000	-8.353521000	-3.169033000
C	1.593820000	-5.248629000	-3.628451000
H	1.333307000	-6.267565000	-3.876269000
C	0.711647000	-4.890260000	1.943417000
C	-3.667726000	-4.155144000	-2.449007000
H	-4.687421000	-3.817405000	-2.660925000

H	-2.980287000	-3.371861000	-2.780202000
H	-3.490295000	-5.046511000	-3.043321000
C	-0.169848000	-6.102230000	-0.187368000
H	0.551271000	-6.906296000	-0.178736000
C	2.687603000	-4.878258000	-2.783849000
C	1.722544000	-3.000899000	-3.696444000
H	1.499099000	-1.969565000	-3.922133000
C	4.682385000	-5.678999000	-1.284810000
H	5.369023000	-4.872949000	-1.542081000
H	4.172485000	-5.427780000	-0.352415000
H	5.290679000	-6.571316000	-1.099402000
C	-0.490055000	-2.668454000	-5.597640000
H	-0.911057000	-2.242845000	-4.681706000
H	0.321761000	-2.015616000	-5.928902000
H	-1.261014000	-2.658953000	-6.374081000
C	4.161894000	-2.488425000	-0.837215000
H	3.290001000	-2.228561000	-0.236052000
H	4.523509000	-3.458802000	-0.513043000
H	4.958862000	-1.761842000	-0.639440000
C	-3.490972000	-4.398311000	-0.946770000
C	0.317600000	-6.002751000	2.933712000
H	-0.735169000	-5.909084000	3.218433000
H	0.455243000	-6.988612000	2.479779000
H	0.930121000	-5.952143000	3.841838000
C	-1.762483000	-7.404812000	-1.680201000
C	3.297845000	-0.990300000	-2.556240000
H	3.166562000	-0.759182000	-3.617034000
H	2.349094000	-0.825090000	-2.039978000
H	4.029599000	-0.280352000	-2.155482000
C	5.076323000	-2.596430000	-3.181760000
H	5.555781000	-3.563850000	-3.019738000
H	4.833961000	-2.519031000	-4.246493000
H	5.806861000	-1.816148000	-2.936771000
C	3.805687000	-2.425534000	-2.329215000
C	-1.148126000	-5.019827000	-5.063286000
H	-0.804512000	-6.047564000	-4.914946000
H	-1.677078000	-4.705258000	-4.159115000
H	-1.872751000	-5.021759000	-5.885305000
C	-2.310947000	-8.313631000	-0.558143000
H	-1.547857000	-8.483586000	0.207166000
H	-3.175491000	-7.859163000	-0.067875000
H	-2.616648000	-9.285767000	-0.963422000
C	3.687554000	-5.984015000	-2.405951000
C	-0.546540000	-8.109215000	-2.300240000
H	-0.124587000	-7.512685000	-3.112517000
H	0.244390000	-8.305763000	-1.575144000

H	-0.854802000	-9.074809000	-2.715320000
C	2.186060000	-5.051422000	1.560034000
H	2.357605000	-6.020930000	1.083196000
H	2.486960000	-4.262443000	0.865830000
H	2.829621000	-4.996358000	2.444965000
C	0.526534000	-3.543401000	2.652997000
H	0.967319000	-2.742914000	2.034155000
H	-0.536198000	-3.355905000	2.885003000
H	1.061740000	-3.529539000	3.605106000
C	2.959190000	-7.267650000	-1.981009000
H	2.393780000	-7.106003000	-1.060003000
H	2.269842000	-7.634999000	-2.742175000
H	3.691015000	-8.060775000	-1.792812000
C	4.493097000	-6.298847000	-3.685168000
H	3.829425000	-6.639742000	-4.485096000
H	5.020434000	-5.412278000	-4.047016000
H	5.233203000	-7.084385000	-3.490608000
Y	3.001401000	1.881367000	4.103907000
C	2.965949000	3.670813000	0.642873000
C	3.809822000	-2.012880000	3.036567000
H	4.200229000	-2.713011000	3.781887000
H	2.790201000	-1.737776000	3.312386000
H	3.754412000	-2.531410000	2.076643000
C	5.508332000	1.189622000	4.605810000
C	4.790340000	-0.008435000	4.219090000
C	2.666215000	3.666415000	2.134339000
C	3.883560000	-0.298084000	5.270168000
H	3.191274000	-1.125590000	5.273930000
C	3.987833000	0.662624000	6.299971000
C	6.067392000	-1.300224000	2.389603000
H	6.537582000	-1.928992000	3.152034000
H	5.910810000	-1.914296000	1.497723000
H	6.768483000	-0.510889000	2.122960000
C	2.416645000	5.017970000	0.128414000
H	1.353456000	5.109453000	0.362554000
H	2.939864000	5.857449000	0.597794000
H	2.544576000	5.094625000	-0.957933000
C	1.317135000	3.887780000	4.020789000
C	6.783737000	2.161568000	2.594244000
H	6.584451000	1.317022000	1.935925000
H	6.011646000	2.915233000	2.421244000
H	7.744262000	2.591441000	2.290127000
C	3.410414000	4.303774000	3.154471000
H	4.404340000	4.709754000	3.027596000
C	3.426161000	0.492984000	7.699304000
C	4.044768000	0.135897000	1.854353000

H	4.090086000	-0.281369000	0.844986000
H	2.965676000	0.231906000	2.052197000
H	4.508457000	1.126744000	1.807382000
C	4.972772000	1.585096000	5.870988000
H	5.344675000	2.409720000	6.462271000
C	2.601543000	4.485796000	4.324702000
C	1.396746000	3.381271000	2.693250000
H	0.596229000	2.867860000	2.172486000
C	2.594507000	5.226757000	6.822565000
H	1.516008000	5.165676000	6.952267000
H	3.040475000	4.294458000	7.177977000
H	2.953108000	6.030055000	7.475395000
C	2.245367000	2.538642000	-0.093739000
H	2.578090000	1.553392000	0.241624000
H	1.164930000	2.581950000	0.050956000
H	2.447842000	2.607794000	-1.167101000
C	0.293397000	2.845289000	6.082435000
H	0.527246000	1.820891000	5.768157000
H	1.103767000	3.219834000	6.706045000
H	-0.603992000	2.776329000	6.707736000
C	4.716301000	-0.780309000	2.894830000
C	4.439162000	-0.372392000	8.475418000
H	4.581880000	-1.338248000	7.981555000
H	5.412321000	0.125808000	8.529188000
H	4.087334000	-0.554942000	9.497366000
C	6.817273000	1.789836000	4.078920000
C	-1.046376000	3.005573000	4.028016000
H	-1.353695000	3.594778000	3.163887000
H	-0.716446000	2.032742000	3.664956000
H	-1.927369000	2.844593000	4.656993000
C	-0.565624000	5.080843000	5.245456000
H	0.063659000	5.657408000	5.922467000
H	-0.744027000	5.686714000	4.351602000
H	-1.528779000	4.923799000	5.743147000
C	0.035063000	3.723588000	4.848265000
C	4.459622000	3.620173000	0.316524000
H	5.023824000	4.391333000	0.850707000
H	4.896124000	2.648027000	0.552311000
H	4.605743000	3.790151000	-0.755393000
C	7.951460000	0.791079000	4.376778000
H	8.011355000	0.605558000	5.453804000
H	7.800441000	-0.168434000	3.884421000
H	8.913230000	1.199479000	4.045276000
C	3.028821000	5.515575000	5.382357000
C	7.168987000	3.088472000	4.817740000
H	6.409321000	3.855005000	4.656600000

H	7.293636000	2.935673000	5.893285000
H	8.117996000	3.475424000	4.432791000
C	3.268323000	1.828230000	8.427008000
H	4.203445000	2.396630000	8.442408000
H	2.498453000	2.443456000	7.957680000
H	2.967524000	1.658616000	9.466082000
C	2.074578000	-0.230826000	7.674945000
H	1.329831000	0.325897000	7.100221000
H	2.159154000	-1.229212000	7.236149000
H	1.693667000	-0.351189000	8.694535000
C	4.557041000	5.645018000	5.448729000
H	5.012295000	4.706669000	5.772390000
H	5.001652000	5.940807000	4.495631000
H	4.824663000	6.414312000	6.180235000
C	2.472188000	6.879535000	4.925907000
H	2.869431000	7.135649000	3.938871000
H	1.383973000	6.871366000	4.852588000
H	2.764340000	7.667493000	5.629922000
Y	-3.118867000	2.232483000	-2.531691000
C	-5.846442000	1.627388000	0.005685000
C	-4.110064000	-1.696733000	-5.243768000
H	-4.156354000	-1.465891000	-6.312745000
H	-3.086462000	-1.988074000	-5.007455000
H	-4.760676000	-2.556920000	-5.050142000
C	-3.754060000	1.997569000	-5.243685000
C	-3.603340000	0.687446000	-4.661720000
C	-4.994043000	2.664362000	-0.703666000
C	-2.233629000	0.547647000	-4.293444000
H	-1.811782000	-0.325953000	-3.813082000
C	-1.527091000	1.733866000	-4.580041000
C	-6.035971000	-0.243143000	-4.653050000
H	-6.264892000	-0.086169000	-5.706681000
H	-6.597359000	-1.125622000	-4.327664000
H	-6.405822000	0.612561000	-4.084727000
C	-6.510005000	2.344258000	1.198841000
H	-5.752396000	2.728668000	1.887775000
H	-7.115624000	3.188714000	0.854080000
H	-7.158875000	1.653968000	1.750339000
C	-3.422725000	4.370839000	-0.907386000
C	-6.253663000	2.629283000	-5.769587000
H	-6.877273000	1.800571000	-6.105530000
H	-6.317855000	2.666620000	-4.681702000
H	-6.708430000	3.544504000	-6.159987000
C	-5.341422000	3.475019000	-1.813367000
H	-6.243764000	3.369366000	-2.400099000
C	-0.017285000	1.891849000	-4.543355000

C	-4.425162000	-0.844232000	-2.914093000
H	-5.112111000	-1.650937000	-2.645596000
H	-3.415397000	-1.135215000	-2.626630000
H	-4.716571000	0.012631000	-2.291065000
C	-2.478057000	2.632944000	-5.125898000
H	-2.236397000	3.607437000	-5.523985000
C	-4.399167000	4.545560000	-1.953643000
C	-3.799872000	3.207441000	-0.181119000
H	-3.246286000	2.793653000	0.649173000
C	-3.467022000	6.042010000	-3.801582000
H	-2.542950000	6.248810000	-3.265466000
H	-3.273898000	5.186088000	-4.451896000
H	-3.684756000	6.908986000	-4.435621000
C	-4.989579000	0.476095000	0.552281000
H	-4.469152000	-0.067535000	-0.238425000
H	-4.230846000	0.847060000	1.246025000
H	-5.617425000	-0.235700000	1.098930000
C	-1.050686000	4.421962000	-1.516423000
H	-0.932033000	3.358464000	-1.257458000
H	-1.345924000	4.537213000	-2.567629000
H	-0.053782000	4.863489000	-1.418805000
C	-4.550172000	-0.489901000	-4.401183000
C	0.549125000	1.082847000	-5.723989000
H	0.281079000	0.029287000	-5.627231000
H	0.145855000	1.447459000	-6.674237000
H	1.642378000	1.161635000	-5.757645000
C	-4.790405000	2.495751000	-6.264775000
C	-1.695427000	4.785957000	0.870890000
H	-2.476930000	5.140489000	1.550874000
H	-1.516770000	3.728886000	1.045317000
H	-0.769832000	5.317304000	1.116607000
C	-2.041366000	6.596353000	-0.710822000
H	-2.262997000	6.987318000	-1.700529000
H	-2.728981000	7.058759000	0.003469000
H	-1.030449000	6.929619000	-0.451672000
C	-2.090101000	5.068278000	-0.587156000
C	-6.956444000	1.087836000	-0.900785000
H	-7.626664000	1.890960000	-1.224240000
H	-6.561516000	0.598988000	-1.793877000
H	-7.559012000	0.351754000	-0.358411000
C	-4.736687000	1.506689000	-7.448242000
H	-3.723747000	1.461315000	-7.860084000
H	-5.016166000	0.495923000	-7.146438000
H	-5.419473000	1.827500000	-8.243836000
C	-4.656373000	5.734965000	-2.879940000
C	-4.369458000	3.857986000	-6.845872000

H	-4.345698000	4.644912000	-6.090410000
H	-3.387770000	3.806519000	-7.324633000
H	-5.093470000	4.157547000	-7.610221000
C	0.411623000	3.351074000	-4.717785000
H	0.058804000	3.760584000	-5.669705000
H	0.036849000	3.987164000	-3.913363000
H	1.504596000	3.420092000	-4.712896000
C	0.569913000	1.330025000	-3.243581000
H	0.248044000	1.899527000	-2.367325000
H	0.273222000	0.289482000	-3.089028000
H	1.663213000	1.366138000	-3.276042000
C	-5.850098000	5.453197000	-3.798189000
H	-5.682960000	4.562930000	-4.399214000
H	-6.776584000	5.319460000	-3.231915000
H	-5.997989000	6.297480000	-4.479758000
C	-5.057831000	6.960410000	-2.037310000
H	-5.952774000	6.725486000	-1.452422000
H	-4.283518000	7.274227000	-1.342778000
H	-5.292450000	7.806769000	-2.693691000

Int6
E= -4854.67534019 Ha
Sum of electronic and thermal Enthalpies= -4851.931808 Ha
Sum of electronic and thermal Free Energies= -4852.248273 Ha

Mo	-0.330903000	-1.036224000	2.271772000
O	-1.767588000	1.490137000	1.716784000
O	-2.637703000	0.757896000	-0.815088000
O	-1.269080000	-1.769083000	-1.765253000
O	-1.595505000	-1.044570000	5.253762000
O	2.165594000	0.689692000	2.491106000
O	0.838366000	-2.323086000	-0.242691000
C	-1.343235000	0.455533000	1.203081000
C	-1.110478000	-1.010349000	4.203544000
C	-1.753040000	0.061329000	-0.187754000
C	-1.088121000	-1.093803000	-0.657209000
C	1.166028000	-0.033217000	2.502738000
C	-0.001674000	-1.485489000	0.238678000
Y	0.039707000	-3.699317000	-1.798188000
C	-0.240464000	-3.460486000	-5.403429000
C	-3.942129000	-3.335767000	0.014928000
H	-3.862572000	-3.543497000	1.086509000
H	-3.293901000	-2.490355000	-0.228108000
H	-4.975810000	-3.034309000	-0.179748000
C	-1.336119000	-6.023758000	-1.217600000
C	-2.146385000	-4.978918000	-0.639816000
C	0.809577000	-3.486694000	-4.312430000

C	-1.385092000	-4.401350000	0.413366000
H	-1.724840000	-3.577686000	1.024792000
C	-0.146610000	-5.053807000	0.547874000
C	-4.530922000	-5.698946000	-0.343000000
H	-4.312098000	-5.930994000	0.704314000
H	-5.579148000	-5.384997000	-0.411797000
H	-4.421390000	-6.620274000	-0.915076000
C	0.350242000	-4.085431000	-6.677350000
H	1.241968000	-3.536587000	-6.995221000
H	0.638576000	-5.127663000	-6.509009000
H	-0.381036000	-4.061151000	-7.493892000
C	2.464786000	-2.820021000	-2.813176000
C	-2.794277000	-7.192696000	-3.068496000
H	-3.761444000	-6.967798000	-2.621943000
H	-2.588280000	-6.448050000	-3.839108000
H	-2.890177000	-8.162341000	-3.569558000
C	1.482845000	-4.630661000	-3.836116000
H	1.329215000	-5.633749000	-4.208076000
C	0.823296000	-4.966604000	1.709436000
C	-3.922835000	-4.187709000	-2.285091000
H	-4.989449000	-3.961958000	-2.394152000
H	-3.362945000	-3.288198000	-2.550454000
H	-3.677267000	-4.968075000	-2.998951000
C	-0.105947000	-6.018313000	-0.481569000
H	0.678827000	-6.751671000	-0.602777000
C	2.539345000	-4.253183000	-2.946627000
C	1.389262000	-2.388351000	-3.646141000
H	1.063835000	-1.365025000	-3.752342000
C	4.671291000	-5.018110000	-1.612941000
H	5.248788000	-4.108450000	-1.776719000
H	4.189058000	-4.960243000	-0.635980000
H	5.383749000	-5.850038000	-1.580590000
C	-0.659863000	-2.023081000	-5.706337000
H	-1.042683000	-1.521554000	-4.813840000
H	0.189939000	-1.444655000	-6.081061000
H	-1.446152000	-2.002252000	-6.467205000
C	3.783572000	-1.995691000	-0.714409000
H	2.915436000	-1.869760000	-0.066835000
H	4.214439000	-2.972816000	-0.522166000
H	4.533169000	-1.247031000	-0.440214000
C	-3.606835000	-4.572196000	-0.837329000
C	0.579057000	-6.208089000	2.589753000
H	-0.453224000	-6.226505000	2.953293000
H	0.747539000	-7.123917000	2.015623000
H	1.253212000	-6.213724000	3.454709000
C	-1.661159000	-7.282844000	-2.041458000

C	2.724670000	-0.389396000	-2.216171000
H	2.566744000	-0.023982000	-3.234733000
H	1.765824000	-0.405720000	-1.695431000
H	3.369386000	0.331650000	-1.706011000
C	4.663314000	-1.677443000	-3.053705000
H	5.252912000	-2.596275000	-3.030142000
H	4.394753000	-1.480648000	-4.096659000
H	5.303377000	-0.859046000	-2.702812000
C	3.395171000	-1.773518000	-2.184565000
C	-1.454791000	-4.282295000	-4.963981000
H	-1.163233000	-5.319896000	-4.780803000
H	-1.895999000	-3.886130000	-4.042028000
H	-2.247634000	-4.279275000	-5.720707000
C	-2.027105000	-8.387222000	-1.026235000
H	-1.197405000	-8.565132000	-0.335932000
H	-2.898457000	-8.104372000	-0.430005000
H	-2.254770000	-9.327647000	-1.542517000
C	3.659903000	-5.284283000	-2.730219000
C	-0.425258000	-7.753976000	-2.823903000
H	-0.110144000	-6.996048000	-3.545382000
H	0.424527000	-7.974416000	-2.176173000
H	-0.662682000	-8.670870000	-3.374051000
C	2.272190000	-4.972321000	1.219371000
H	2.476067000	-5.868516000	0.626773000
H	2.465624000	-4.092761000	0.601414000
H	2.970141000	-4.968156000	2.063785000
C	0.595071000	-3.727741000	2.588539000
H	0.951207000	-2.831065000	2.042243000
H	-0.457253000	-3.652110000	2.891020000
H	1.191613000	-3.792058000	3.502093000
C	3.076931000	-6.677661000	-2.448984000
H	2.523690000	-6.680565000	-1.507068000
H	2.404222000	-7.020825000	-3.236373000
H	3.888310000	-7.409431000	-2.369807000
C	4.442034000	-5.353534000	-4.060134000
H	3.786763000	-5.658275000	-4.880967000
H	4.862608000	-4.377671000	-4.317348000
H	5.264329000	-6.075388000	-3.986197000
Y	3.887154000	1.767827000	3.349882000
C	6.236147000	2.631377000	0.293928000
C	3.922195000	-2.309934000	3.500242000
H	3.635767000	-2.819555000	4.425423000
H	3.045205000	-1.792646000	3.103415000
H	4.206815000	-3.070030000	2.767353000
C	5.493071000	0.868811000	5.245994000
C	4.738197000	-0.254922000	4.725693000

C	5.174195000	3.011221000	1.314373000
C	3.390801000	-0.071975000	5.136470000
H	2.569613000	-0.727022000	4.883449000
C	3.259933000	1.120205000	5.881794000
C	6.283063000	-2.235495000	4.185109000
H	6.056506000	-2.691256000	5.153850000
H	6.444131000	-3.042024000	3.461491000
H	7.220343000	-1.690091000	4.281738000
C	6.443925000	3.892208000	-0.571876000
H	5.500290000	4.209649000	-1.025251000
H	6.822252000	4.721031000	0.034523000
H	7.164148000	3.693241000	-1.373858000
C	3.104382000	3.868128000	1.973326000
C	7.852495000	0.946558000	4.204042000
H	7.766787000	-0.021147000	3.711113000
H	7.571642000	1.720342000	3.487238000
H	8.909544000	1.091918000	4.451876000
C	5.353446000	3.799222000	2.472790000
H	6.314671000	4.044842000	2.902286000
C	2.063525000	1.514047000	6.723928000
C	5.403567000	-0.699975000	2.368696000
H	5.795914000	-1.418897000	1.641918000
H	4.472102000	-0.332644000	1.905841000
H	6.131421000	0.113656000	2.441876000
C	4.548872000	1.699265000	5.922485000
H	4.803621000	2.584245000	6.486806000
C	4.105634000	4.373137000	2.890507000
C	3.784237000	3.025544000	1.051425000
H	3.302860000	2.495094000	0.243224000
C	2.887203000	5.902237000	4.630378000
H	2.011004000	6.086334000	4.012397000
H	2.658710000	5.093347000	5.325273000
H	3.057851000	6.805711000	5.225596000
C	5.765383000	1.492802000	-0.612292000
H	5.544908000	0.589098000	-0.039326000
H	4.866160000	1.769145000	-1.167923000
H	6.540335000	1.246011000	-1.345201000
C	0.825105000	3.712159000	3.104666000
H	0.900130000	2.634156000	3.277314000
H	1.174717000	4.240883000	3.989061000
H	-0.241522000	3.917782000	2.983765000
C	5.109380000	-1.360354000	3.729064000
C	2.305320000	0.944949000	8.135163000
H	2.439005000	-0.140446000	8.095293000
H	3.204338000	1.380290000	8.583407000
H	1.453052000	1.163434000	8.788474000

C	6.995928000	1.076719000	5.465045000
C	1.014756000	3.258789000	0.692857000
H	1.420051000	3.557010000	-0.278866000
H	1.189591000	2.191720000	0.839848000
H	-0.067118000	3.398548000	0.666377000
C	1.332089000	5.575224000	1.455607000
H	1.640640000	6.283205000	2.224372000
H	1.868574000	5.826898000	0.534791000
H	0.262844000	5.725823000	1.276496000
C	1.596464000	4.108553000	1.835545000
C	7.579771000	2.252082000	0.915996000
H	7.926036000	3.004317000	1.631323000
H	7.530177000	1.287707000	1.425232000
H	8.340763000	2.169122000	0.133054000
C	7.455660000	0.077315000	6.543257000
H	6.881055000	0.226960000	7.462512000
H	7.317165000	-0.957947000	6.231225000
H	8.516940000	0.227363000	6.773126000
C	4.150107000	5.595619000	3.822248000
C	7.278494000	2.487003000	6.005975000
H	6.916330000	3.256254000	5.319832000
H	6.826651000	2.655954000	6.987238000
H	8.359069000	2.619779000	6.120093000
C	1.911188000	3.031870000	6.819132000
H	2.830018000	3.517199000	7.163373000
H	1.640039000	3.449713000	5.847403000
H	1.113596000	3.291448000	7.522913000
C	0.776292000	0.914260000	6.157901000
H	0.594270000	1.231579000	5.126599000
H	0.810450000	-0.177298000	6.167572000
H	-0.085355000	1.218798000	6.759918000
C	5.275112000	5.452477000	4.859133000
H	5.113624000	4.573917000	5.489198000
H	6.265276000	5.374922000	4.404171000
H	5.289507000	6.334484000	5.507420000
C	4.478234000	6.808932000	2.927526000
H	5.427020000	6.653536000	2.404982000
H	3.705246000	6.968184000	2.172073000
H	4.561529000	7.719866000	3.531633000
Y	-3.829805000	2.124238000	-1.961553000
C	-6.472114000	1.040075000	0.505237000
C	-4.181433000	-1.690125000	-4.848126000
H	-4.171957000	-1.443253000	-5.914634000
H	-3.151431000	-1.856864000	-4.534552000
H	-4.732259000	-2.627632000	-4.715889000
C	-4.300638000	2.049381000	-4.698513000

C	-4.010883000	0.724612000	-4.214569000
C	-5.751426000	2.213424000	-0.135284000
C	-2.651099000	0.718387000	-3.786784000
H	-2.149135000	-0.133756000	-3.342975000
C	-2.085363000	2.001241000	-3.949040000
C	-6.309868000	-0.501451000	-4.402399000
H	-6.475382000	-0.319639000	-5.464243000
H	-6.764197000	-1.469474000	-4.164615000
H	-6.840689000	0.260209000	-3.828400000
C	-7.183699000	1.592535000	1.756881000
H	-6.456834000	1.995454000	2.468052000
H	-7.873873000	2.397743000	1.485009000
H	-7.754235000	0.801102000	2.257191000
C	-4.403722000	4.107523000	-0.227904000
C	-6.833920000	2.403487000	-5.311869000
H	-7.318226000	1.521012000	-5.730130000
H	-6.953372000	2.362092000	-4.228662000
H	-7.393212000	3.271345000	-5.673770000
C	-6.200909000	3.042848000	-1.193922000
H	-7.081038000	2.859059000	-1.794898000
C	-0.610842000	2.340091000	-3.816768000
C	-4.773132000	-0.898364000	-2.541253000
H	-5.390531000	-1.767819000	-2.300906000
H	-3.759502000	-1.079581000	-2.182413000
H	-5.208727000	-0.073312000	-1.954271000
C	-3.117380000	2.822133000	-4.467889000
H	-2.985496000	3.849554000	-4.775059000
C	-5.401588000	4.228400000	-1.264577000
C	-4.634815000	2.868026000	0.428805000
H	-4.021715000	2.465757000	1.222771000
C	-4.722189000	6.109114000	-2.882974000
H	-3.941726000	6.536641000	-2.256395000
H	-4.248184000	5.384124000	-3.551384000
H	-5.126107000	6.921211000	-3.497706000
C	-5.488685000	-0.051106000	0.952829000
H	-4.979553000	-0.519051000	0.109074000
H	-4.715631000	0.357985000	1.609385000
H	-6.020841000	-0.834455000	1.503710000
C	-2.122870000	4.822601000	-0.899920000
H	-1.777729000	3.778283000	-0.928777000
H	-2.480779000	5.104970000	-1.893187000
H	-1.230378000	5.420083000	-0.681199000
C	-4.828925000	-0.560151000	-4.037056000
C	0.122392000	1.647714000	-4.979533000
H	-0.001958000	0.564653000	-4.921184000
H	-0.274730000	1.980501000	-5.943920000

H	1.194773000	1.874239000	-4.948088000
C	-5.344875000	2.504363000	-5.730441000
C	-2.636126000	4.520800000	1.511938000
H	-3.394851000	4.572140000	2.299434000
H	-2.253256000	3.500854000	1.473309000
H	-1.815099000	5.185036000	1.800395000
C	-3.537553000	6.477157000	0.370988000
H	-3.872108000	6.990684000	-0.527514000
H	-4.306509000	6.596896000	1.140175000
H	-2.636181000	6.992650000	0.720870000
C	-3.213099000	4.990324000	0.168770000
C	-7.538997000	0.452321000	-0.423743000
H	-8.301632000	1.199200000	-0.667207000
H	-7.117694000	0.084272000	-1.362268000
H	-8.042485000	-0.387493000	0.066668000
C	-5.108238000	1.642124000	-6.988101000
H	-4.080535000	1.763144000	-7.344369000
H	-5.263691000	0.580639000	-6.785786000
H	-5.791017000	1.942383000	-7.791772000
C	-5.850869000	5.441646000	-2.084071000
C	-5.075428000	3.957274000	-6.163794000
H	-5.164960000	4.663873000	-5.336452000
H	-4.080676000	4.066197000	-6.604345000
H	-5.806635000	4.245385000	-6.925638000
C	-0.364099000	3.846897000	-3.922963000
H	-0.692192000	4.237507000	-4.891764000
H	-0.885094000	4.400339000	-3.139323000
H	0.706231000	4.057452000	-3.825903000
C	-0.032913000	1.807616000	-2.501668000
H	-0.484798000	2.283067000	-1.627122000
H	-0.195509000	0.731617000	-2.409316000
H	1.045204000	1.990841000	-2.457116000
C	-6.913466000	5.032869000	-3.109174000
H	-6.532372000	4.270792000	-3.783370000
H	-7.819579000	4.649850000	-2.631288000
H	-7.201918000	5.902087000	-3.709395000
C	-6.539501000	6.450323000	-1.143755000
H	-7.396166000	5.972344000	-0.658244000
H	-5.882660000	6.821309000	-0.360182000
H	-6.908299000	7.309132000	-1.717294000

TS4
E= -4854.63380052 Ha
Sum of electronic and thermal Enthalpies= -4851.890609 Ha
Sum of electronic and thermal Free Energies= -4852.201165 Ha
Imag_freq=-332.5 cm-1

Mo	-0.700841000	-1.138049000	1.668576000
O	-1.871278000	1.704599000	1.416494000
O	-2.255653000	0.735058000	-1.191928000
O	-0.672224000	-1.560409000	-1.929504000
O	-2.309600000	-0.963382000	4.409468000
O	2.079279000	0.248037000	1.694825000
O	1.524962000	-2.039857000	-0.376825000
C	-1.329922000	0.681462000	1.018455000
C	-1.734372000	-1.075112000	3.402278000
C	-1.292189000	0.295117000	-0.450697000
C	-0.457866000	-0.810279000	-0.855316000
C	0.973144000	-0.307337000	1.622624000
C	0.610658000	-1.231549000	0.007783000
Y	0.522481000	-3.499665000	-1.788279000
C	-0.157247000	-3.590379000	-5.431457000
C	-3.248832000	-2.783556000	0.362364000
H	-3.054746000	-2.931384000	1.430306000
H	-2.587893000	-2.003481000	-0.035445000
H	-4.269463000	-2.402683000	0.283700000
C	-1.037603000	-5.704346000	-1.078033000
C	-1.693024000	-4.617391000	-0.394074000
C	0.955863000	-3.601769000	-4.405132000
C	-0.789537000	-4.154086000	0.601202000
H	-1.058379000	-3.453629000	1.390451000
C	0.399983000	-4.904557000	0.582778000
C	-4.085034000	-5.091238000	0.191225000
H	-3.778292000	-5.304414000	1.220358000
H	-5.101497000	-4.681405000	0.215169000
H	-4.120024000	-6.039004000	-0.346459000
C	0.348880000	-4.295064000	-6.701631000
H	1.240573000	-3.793412000	-7.090134000
H	0.611518000	-5.337218000	-6.495247000
H	-0.421628000	-4.284069000	-7.481712000
C	2.818807000	-2.922207000	-3.185271000
C	-2.819453000	-6.820000000	-2.686119000
H	-3.699722000	-6.504413000	-2.127487000
H	-2.670738000	-6.131578000	-3.518989000
H	-3.049854000	-7.801690000	-3.114731000
C	1.610281000	-4.742560000	-3.894892000
H	1.342964000	-5.761409000	-4.133087000
C	1.466617000	-4.953557000	1.652566000
C	-3.538710000	-3.726081000	-1.876171000
H	-4.589181000	-3.421363000	-1.894064000
H	-2.931772000	-2.890239000	-2.232751000
H	-3.421965000	-4.550971000	-2.570391000
C	0.260297000	-5.816402000	-0.481441000

H	0.966463000	-6.601317000	-0.708471000
C	2.788647000	-4.365259000	-3.179194000
C	1.681087000	-2.492423000	-3.928027000
H	1.410112000	-1.462665000	-4.100273000
C	4.994474000	-5.169825000	-2.001287000
H	5.611256000	-4.317590000	-2.286800000
H	4.608854000	-5.003935000	-0.993872000
H	5.654057000	-6.043940000	-1.962689000
C	-0.532515000	-2.152715000	-5.785329000
H	-0.832327000	-1.588529000	-4.899504000
H	0.317245000	-1.637251000	-6.242798000
H	-1.362360000	-2.131913000	-6.497765000
C	4.404903000	-2.004228000	-1.349226000
H	3.623661000	-1.724704000	-0.641671000
H	4.751822000	-2.999633000	-1.090846000
H	5.250185000	-1.318273000	-1.233471000
C	-3.117476000	-4.081237000	-0.450705000
C	1.242223000	-6.238181000	2.472101000
H	0.239110000	-6.245809000	2.910006000
H	1.340395000	-7.126532000	1.840509000
H	1.974795000	-6.311416000	3.284838000
C	-1.567949000	-6.955305000	-1.811354000
C	3.310278000	-0.471728000	-2.904385000
H	3.051833000	-0.209776000	-3.934188000
H	2.422104000	-0.354060000	-2.278699000
H	4.063640000	0.243904000	-2.565263000
C	5.054121000	-1.993785000	-3.780774000
H	5.589967000	-2.941024000	-3.693833000
H	4.690008000	-1.907433000	-4.809547000
H	5.773717000	-1.185728000	-3.601159000
C	3.882355000	-1.894657000	-2.786366000
C	-1.377223000	-4.345661000	-4.902952000
H	-1.124110000	-5.392689000	-4.720110000
H	-1.736293000	-3.916685000	-3.961880000
H	-2.208410000	-4.319252000	-5.616485000
C	-1.887431000	-7.983098000	-0.702005000
H	-0.990488000	-8.215396000	-0.120862000
H	-2.638955000	-7.596453000	-0.008801000
H	-2.268409000	-8.914186000	-1.138334000
C	3.868960000	-5.446110000	-3.001611000
C	-0.488088000	-7.571946000	-2.714835000
H	-0.230031000	-6.900087000	-3.535683000
H	0.428465000	-7.812198000	-2.174175000
H	-0.863700000	-8.504831000	-3.148636000
C	2.852506000	-5.006787000	1.010654000
H	2.925266000	-5.857889000	0.328893000

H	3.048142000	-4.092148000	0.444618000
H	3.633736000	-5.123524000	1.767806000
C	1.360375000	-3.754213000	2.594872000
H	1.503149000	-2.815345000	2.051756000
H	0.384904000	-3.724105000	3.094275000
H	2.120489000	-3.814704000	3.380178000
C	3.247110000	-6.787481000	-2.581369000
H	2.785248000	-6.710707000	-1.595199000
H	2.490402000	-7.141048000	-3.283558000
H	4.027091000	-7.554732000	-2.530145000
C	4.510037000	-5.644519000	-4.392922000
H	3.756792000	-5.954069000	-5.123066000
H	4.961704000	-4.717686000	-4.755533000
H	5.289924000	-6.414532000	-4.351418000
Y	3.800087000	1.322725000	2.723024000
C	6.159934000	2.884236000	-0.072150000
C	4.693215000	-2.614475000	2.281458000
H	4.480360000	-3.304252000	3.102340000
H	3.746921000	-2.217443000	1.907390000
H	5.143903000	-3.186575000	1.466668000
C	5.573668000	0.474118000	4.550132000
C	5.049590000	-0.673209000	3.844961000
C	5.069127000	2.985005000	0.983995000
C	3.679724000	-0.799746000	4.207941000
H	3.005669000	-1.558371000	3.837743000
C	3.317738000	0.233796000	5.103077000
C	6.970818000	-2.222388000	3.082195000
H	6.814937000	-2.881459000	3.941711000
H	7.292245000	-2.842268000	2.238193000
H	7.788456000	-1.546140000	3.321349000
C	6.181913000	4.267879000	-0.758318000
H	5.202164000	4.504525000	-1.183218000
H	6.436020000	5.052498000	-0.038956000
H	6.924027000	4.284088000	-1.564822000
C	2.936175000	3.581301000	1.715902000
C	7.927152000	1.097436000	3.677143000
H	8.019979000	0.236033000	3.017796000
H	7.546851000	1.932817000	3.086646000
H	8.936847000	1.357906000	4.012898000
C	5.170291000	3.569946000	2.265453000
H	6.102435000	3.800791000	2.761849000
C	2.050130000	0.305572000	5.928916000
C	5.840789000	-0.582829000	1.509556000
H	6.403083000	-1.060502000	0.701226000
H	4.862721000	-0.352498000	1.049605000
H	6.356491000	0.346889000	1.762264000

C	4.477798000	1.023090000	5.280237000
H	4.549852000	1.853305000	5.966942000
C	3.879446000	3.988398000	2.733479000
C	3.685859000	2.949493000	0.686464000
H	3.258868000	2.548304000	-0.220806000
C	2.517136000	5.142762000	4.652770000
H	1.634361000	5.367551000	4.058665000
H	2.341758000	4.212622000	5.197418000
H	2.617508000	5.942204000	5.394765000
C	5.831633000	1.829343000	-1.131378000
H	5.762184000	0.828344000	-0.700773000
H	4.883264000	2.047304000	-1.628656000
H	6.609291000	1.811093000	-1.901956000
C	0.641364000	3.161359000	2.734433000
H	0.732164000	2.070678000	2.726888000
H	0.967786000	3.535752000	3.701366000
H	-0.427567000	3.358561000	2.630256000
C	5.660001000	-1.512452000	2.720358000
C	2.316617000	-0.475424000	7.229310000
H	2.577742000	-1.514868000	7.007838000
H	3.144824000	-0.030197000	7.790222000
H	1.425520000	-0.473115000	7.867005000
C	7.018891000	0.862830000	4.887782000
C	0.924826000	3.099693000	0.285600000
H	1.321376000	3.585059000	-0.611688000
H	1.178462000	2.038920000	0.250387000
H	-0.163072000	3.163994000	0.261875000
C	1.123489000	5.276363000	1.405148000
H	1.312948000	5.857042000	2.307290000
H	1.732275000	5.697420000	0.598088000
H	0.072790000	5.410269000	1.142446000
C	1.428602000	3.778387000	1.568255000
C	7.554914000	2.621281000	0.495886000
H	7.811750000	3.335193000	1.284328000
H	7.653474000	1.612100000	0.899064000
H	8.300754000	2.728912000	-0.298516000
C	7.588900000	-0.236612000	5.805881000
H	6.977085000	-0.319218000	6.709545000
H	7.605104000	-1.215309000	5.328756000
H	8.612219000	0.015750000	6.107253000
C	3.807455000	5.065617000	3.831858000
C	7.071173000	2.168588000	5.691402000
H	6.670361000	3.003653000	5.117422000
H	6.526361000	2.098903000	6.636955000
H	8.113519000	2.398479000	5.934095000
C	1.689446000	1.751253000	6.276505000

H	2.500693000	2.263688000	6.803471000
H	1.450812000	2.316044000	5.371840000
H	0.805331000	1.777073000	6.921375000
C	0.874804000	-0.330739000	5.190311000
H	0.652829000	0.191374000	4.255938000
H	1.066624000	-1.379228000	4.943605000
H	-0.028136000	-0.298662000	5.806043000
C	4.927357000	4.888731000	4.864779000
H	4.812021000	3.948943000	5.408467000
H	5.924392000	4.917945000	4.419634000
H	4.880340000	5.704103000	5.593686000
C	4.043943000	6.416691000	3.123868000
H	5.018415000	6.418765000	2.625713000
H	3.283515000	6.615369000	2.366925000
H	4.027523000	7.236218000	3.851704000
Y	-3.548664000	2.079805000	-2.343145000
C	-6.343586000	1.249665000	0.097561000
C	-3.942026000	-1.875517000	-4.807287000
H	-3.865523000	-1.813732000	-5.897333000
H	-2.936561000	-1.996578000	-4.408241000
H	-4.517026000	-2.772122000	-4.551993000
C	-4.034676000	1.906109000	-5.117284000
C	-3.756439000	0.611322000	-4.553130000
C	-5.562048000	2.337491000	-0.618033000
C	-2.398164000	0.618621000	-4.117878000
H	-1.903562000	-0.198645000	-3.606119000
C	-1.825441000	1.890244000	-4.347034000
C	-6.065572000	-0.608353000	-4.732994000
H	-6.149083000	-0.566910000	-5.819518000
H	-6.546739000	-1.536788000	-4.406335000
H	-6.629685000	0.221368000	-4.305537000
C	-7.013839000	1.921536000	1.313482000
H	-6.260974000	2.319133000	1.999629000
H	-7.653276000	2.750429000	0.993001000
H	-7.630898000	1.198776000	1.859944000
C	-4.183157000	4.202739000	-0.781632000
C	-6.540768000	2.216182000	-5.888655000
H	-7.007239000	1.315904000	-6.290405000
H	-6.722772000	2.230924000	-4.814144000
H	-7.071697000	3.068891000	-6.322248000
C	-5.940084000	3.085380000	-1.760151000
H	-6.791902000	2.867302000	-2.389225000
C	-0.348444000	2.238495000	-4.277036000
C	-4.688640000	-0.697108000	-2.718088000
H	-5.327723000	-1.513960000	-2.371734000
H	-3.707724000	-0.810905000	-2.251326000

H	-5.183723000	0.209669000	-2.326751000
C	-2.851876000	2.683104000	-4.915957000
H	-2.708103000	3.686357000	-5.288417000
C	-5.120833000	4.254479000	-1.876108000
C	-4.458735000	3.012057000	-0.053205000
H	-3.905814000	2.669993000	0.809764000
C	-4.328337000	6.001908000	-3.584147000
H	-3.580890000	6.463807000	-2.941999000
H	-3.827468000	5.224865000	-4.167703000
H	-4.682419000	6.772826000	-4.277709000
C	-5.414849000	0.141911000	0.611466000
H	-4.940592000	-0.399827000	-0.207575000
H	-4.613699000	0.547891000	1.235322000
H	-5.978817000	-0.579872000	1.212776000
C	-1.850940000	4.896144000	-1.292458000
H	-1.524073000	3.846883000	-1.253427000
H	-2.111451000	5.145120000	-2.323811000
H	-0.975754000	5.493103000	-1.012587000
C	-4.618173000	-0.620546000	-4.248176000
C	0.329515000	1.576948000	-5.490700000
H	0.210185000	0.492325000	-5.451353000
H	-0.114113000	1.931352000	-6.426757000
H	1.401565000	1.807028000	-5.505141000
C	-5.029212000	2.285143000	-6.228832000
C	-2.611774000	4.763820000	1.085663000
H	-3.466453000	4.822333000	1.766847000
H	-2.188818000	3.761759000	1.154626000
H	-1.866905000	5.483095000	1.436266000
C	-3.382653000	6.620081000	-0.290585000
H	-3.633856000	7.060102000	-1.253119000
H	-4.216634000	6.793695000	0.396033000
H	-2.516175000	7.166996000	0.097309000
C	-3.038262000	5.124989000	-0.345770000
C	-7.452499000	0.665098000	-0.783275000
H	-8.168442000	1.440530000	-1.074656000
H	-7.068676000	0.203766000	-1.696192000
H	-8.002986000	-0.103498000	-0.230470000
C	-4.729594000	1.325490000	-7.400948000
H	-3.683157000	1.415617000	-7.708354000
H	-4.902282000	0.283439000	-7.125008000
H	-5.365746000	1.563826000	-8.261710000
C	-5.510565000	5.402481000	-2.811449000
C	-4.725479000	3.697076000	-6.766178000
H	-4.829079000	4.470041000	-6.002703000
H	-3.715572000	3.757547000	-7.180787000
H	-5.427332000	3.931172000	-7.572653000

C	-0.117607000	3.749774000	-4.358956000
H	-0.498376000	4.166596000	-5.296879000
H	-0.597318000	4.277161000	-3.531541000
H	0.955325000	3.965039000	-4.314040000
C	0.298230000	1.690578000	-3.003051000
H	-0.114569000	2.157882000	-2.105343000
H	0.146935000	0.612264000	-2.914733000
H	1.376296000	1.883127000	-3.011951000
C	-6.503791000	4.906832000	-3.865233000
H	-6.083764000	4.073951000	-4.421330000
H	-7.449284000	4.584465000	-3.420035000
H	-6.731515000	5.711405000	-4.572055000
C	-6.256395000	6.481682000	-2.001772000
H	-7.136397000	6.039360000	-1.524109000
H	-5.646539000	6.927919000	-1.220111000
H	-6.598120000	7.282273000	-2.668742000

Int7

E= -4854.66670638 Ha

Sum of electronic and thermal Enthalpies= -4851.917323 Ha

Sum of electronic and thermal Free Energies= -4852.224894 Ha

Mo	-1.004693000	-1.068687000	1.952142000
O	-2.184130000	1.804822000	1.709555000
O	-2.628770000	0.612392000	-0.871830000
O	-0.731019000	-1.346635000	-1.593092000
O	-1.505115000	0.215986000	4.766548000
O	1.838850000	0.225855000	2.139035000
O	1.495946000	-1.654991000	-0.207429000
C	-1.663363000	0.767908000	1.313767000
C	-1.284585000	-0.337400000	3.767402000
C	-1.616446000	0.329398000	-0.112695000
C	-0.596880000	-0.604078000	-0.495330000
C	0.764877000	-0.261537000	1.629941000
C	0.630436000	-0.776065000	0.259734000
Y	0.547986000	-3.111246000	-1.554380000
C	-0.145232000	-3.377603000	-5.336780000
C	-3.702700000	-2.581931000	2.059788000
H	-3.304454000	-3.018674000	2.978447000
H	-3.214163000	-1.609346000	1.848060000
H	-4.741524000	-2.304655000	2.254981000
C	-1.668307000	-4.956233000	-0.324825000
C	-2.216755000	-4.050965000	0.628359000
C	0.879157000	-3.422808000	-4.221329000
C	-1.149928000	-3.670332000	1.515568000
H	-1.330435000	-3.336815000	2.544048000
C	0.037149000	-4.362885000	1.170098000

C	-4.622023000	-4.667191000	1.169354000
H	-4.283680000	-5.236248000	2.041382000
H	-5.612561000	-4.255176000	1.393004000
H	-4.738939000	-5.363106000	0.340301000
C	0.531797000	-3.940727000	-6.601711000
H	1.428164000	-3.363373000	-6.848279000
H	0.831100000	-4.983118000	-6.454619000
H	-0.153568000	-3.896264000	-7.456527000
C	2.795822000	-2.841709000	-3.025852000
C	-3.488669000	-5.541083000	-2.098001000
H	-4.356273000	-5.263790000	-1.500740000
H	-3.218173000	-4.685361000	-2.720727000
H	-3.802614000	-6.351031000	-2.765735000
C	1.436436000	-4.587142000	-3.651786000
H	1.088665000	-5.591022000	-3.848555000
C	1.240387000	-4.590840000	2.053480000
C	-4.114864000	-2.717491000	-0.357960000
H	-5.144750000	-2.374737000	-0.209349000
H	-3.473384000	-1.842783000	-0.499690000
H	-4.079214000	-3.299135000	-1.272802000
C	-0.264950000	-5.098960000	0.009242000
H	0.376412000	-5.874307000	-0.394222000
C	2.637500000	-4.276182000	-2.945614000
C	1.694865000	-2.355979000	-3.792664000
H	1.527293000	-1.318543000	-4.038076000
C	4.696511000	-5.202589000	-1.616892000
H	5.402532000	-4.438065000	-1.940682000
H	4.294774000	-4.917086000	-0.643640000
H	5.264997000	-6.129302000	-1.481638000
C	-0.562074000	-1.933213000	-5.619285000
H	-0.907304000	-1.424512000	-4.717876000
H	0.279133000	-1.361586000	-6.022608000
H	-1.368209000	-1.898395000	-6.359581000
C	4.413886000	-1.928132000	-1.225647000
H	3.634047000	-1.533923000	-0.571538000
H	4.670091000	-2.925762000	-0.882057000
H	5.307326000	-1.303721000	-1.125949000
C	-3.636781000	-3.528885000	0.850628000
C	1.033158000	-5.934988000	2.780607000
H	0.112817000	-5.913726000	3.372815000
H	0.952466000	-6.757222000	2.063197000
H	1.872706000	-6.143750000	3.454464000
C	-2.299255000	-6.008886000	-1.250950000
C	3.529055000	-0.434760000	-2.947481000
H	3.314002000	-0.247802000	-4.003568000
H	2.649762000	-0.164939000	-2.355286000

H	4.348817000	0.228688000	-2.659066000
C	5.127348000	-2.195001000	-3.628089000
H	5.562938000	-3.178933000	-3.444442000
H	4.800232000	-2.163451000	-4.672410000
H	5.919957000	-1.449580000	-3.493182000
C	3.949031000	-1.892037000	-2.684900000
C	-1.362553000	-4.246304000	-5.015074000
H	-1.082886000	-5.300428000	-4.934840000
H	-1.825433000	-3.950885000	-4.070975000
H	-2.121024000	-4.163466000	-5.801277000
C	-2.729582000	-7.208659000	-0.379362000
H	-1.869560000	-7.595775000	0.175862000
H	-3.494978000	-6.934262000	0.347166000
H	-3.126040000	-8.015939000	-1.007139000
C	3.588226000	-5.446152000	-2.644551000
C	-1.252449000	-6.548278000	-2.240357000
H	-0.793472000	-5.742436000	-2.814807000
H	-0.455046000	-7.103175000	-1.740685000
H	-1.729481000	-7.233619000	-2.948530000
C	2.510507000	-4.679288000	1.208894000
H	2.424674000	-5.477575000	0.468653000
H	2.702361000	-3.734135000	0.691656000
H	3.381709000	-4.908501000	1.829846000
C	1.382458000	-3.493922000	3.103832000
H	1.510015000	-2.510135000	2.642601000
H	0.504774000	-3.449490000	3.755779000
H	2.246414000	-3.696427000	3.744687000
C	2.801381000	-6.678284000	-2.162105000
H	2.255543000	-6.468105000	-1.239290000
H	2.082360000	-7.035709000	-2.902333000
H	3.493953000	-7.501916000	-1.959768000
C	4.257987000	-5.819951000	-3.984758000
H	3.504916000	-6.094045000	-4.729146000
H	4.831359000	-4.980909000	-4.386279000
H	4.938401000	-6.669368000	-3.851016000
Y	3.548334000	1.241706000	3.071237000
C	5.720389000	3.069933000	0.259433000
C	4.796046000	-2.594209000	2.332989000
H	4.644948000	-3.330457000	3.127305000
H	3.819909000	-2.223851000	2.012194000
H	5.244738000	-3.106393000	1.477182000
C	5.556321000	0.425430000	4.732640000
C	5.094617000	-0.712108000	3.971628000
C	4.686657000	3.056465000	1.377090000
C	3.770006000	-0.993684000	4.403743000
H	3.145701000	-1.781628000	4.016774000

C	3.372965000	-0.068168000	5.390625000
C	7.076544000	-2.110684000	3.084155000
H	6.987097000	-2.810347000	3.920995000
H	7.404583000	-2.678426000	2.206355000
H	7.863375000	-1.398104000	3.324203000
C	5.599999000	4.471944000	-0.379187000
H	4.586401000	4.641542000	-0.753796000
H	5.822117000	5.251401000	0.356204000
H	6.301366000	4.573986000	-1.215621000
C	2.565901000	3.550823000	2.212528000
C	7.841551000	1.242819000	3.830329000
H	7.961857000	0.421113000	3.126582000
H	7.399910000	2.084241000	3.293811000
H	8.844448000	1.541810000	4.154402000
C	4.819458000	3.577121000	2.683922000
H	5.762316000	3.807011000	3.160629000
C	2.140866000	-0.152015000	6.267562000
C	5.812397000	-0.471943000	1.623444000
H	6.348027000	-0.892927000	0.766935000
H	4.804538000	-0.240643000	1.236590000
H	6.305151000	0.457978000	1.905641000
C	4.461048000	0.820549000	5.557204000
H	4.499569000	1.602905000	6.301646000
C	3.533924000	3.923147000	3.220074000
C	3.295569000	2.993340000	1.129666000
H	2.851063000	2.630861000	0.215134000
C	2.183271000	4.856365000	5.249069000
H	1.283710000	5.128022000	4.701758000
H	2.036955000	3.861184000	5.675067000
H	2.280866000	5.562630000	6.080523000
C	5.412122000	2.028871000	-0.820686000
H	5.465774000	1.010236000	-0.433803000
H	4.413583000	2.173019000	-1.241569000
H	6.130861000	2.115261000	-1.642745000
C	0.270059000	3.035876000	3.200962000
H	0.414221000	1.955531000	3.116535000
H	0.564678000	3.350138000	4.199169000
H	-0.803020000	3.203895000	3.086741000
C	5.717565000	-1.460647000	2.791955000
C	2.599492000	-0.469602000	7.701788000
H	3.181291000	-1.396478000	7.725358000
H	3.225869000	0.332917000	8.104341000
H	1.732796000	-0.589117000	8.361893000
C	6.983335000	0.892086000	5.048500000
C	0.558473000	3.192201000	0.757183000
H	0.988580000	3.735899000	-0.089214000

H	0.789209000	2.130594000	0.645106000
H	-0.526478000	3.288147000	0.714089000
C	0.732098000	5.256922000	2.062686000
H	0.923344000	5.761845000	3.008546000
H	1.323791000	5.755055000	1.287217000
H	-0.324022000	5.394368000	1.825233000
C	1.055693000	3.754638000	2.096307000
C	7.161960000	2.903927000	0.740396000
H	7.403535000	3.613904000	1.537398000
H	7.364942000	1.895770000	1.103793000
H	7.850552000	3.096309000	-0.089178000
C	7.647214000	-0.217807000	5.889498000
H	7.073180000	-0.381428000	6.806912000
H	7.701375000	-1.168538000	5.361164000
H	8.664954000	0.077920000	6.170577000
C	3.455320000	4.909208000	4.397267000
C	6.981320000	2.151361000	5.924104000
H	6.515792000	2.989947000	5.408727000
H	6.466278000	1.995634000	6.875687000
H	8.014399000	2.429899000	6.155674000
C	1.382339000	1.177324000	6.269797000
H	2.035505000	1.992011000	6.598247000
H	0.994795000	1.415276000	5.275652000
H	0.523023000	1.139532000	6.946792000
C	1.225068000	-1.274528000	5.780675000
H	0.944878000	-1.128438000	4.734561000
H	1.719947000	-2.246591000	5.868396000
H	0.307216000	-1.303252000	6.374685000
C	4.610906000	4.695940000	5.381914000
H	4.561893000	3.703683000	5.833721000
H	5.589669000	4.819800000	4.913288000
H	4.546225000	5.436125000	6.185898000
C	3.621442000	6.324399000	3.804801000
H	4.582094000	6.406228000	3.286731000
H	2.835832000	6.559493000	3.085619000
H	3.595135000	7.077140000	4.601523000
Y	-3.769540000	2.032565000	-2.091210000
C	-6.450557000	1.259922000	0.524399000
C	-3.558155000	-1.856237000	-3.614721000
H	-2.855439000	-2.092467000	-4.410587000
H	-2.992291000	-1.631262000	-2.709449000
H	-4.166718000	-2.746130000	-3.428694000
C	-3.982764000	1.840646000	-4.868235000
C	-3.636820000	0.557514000	-4.293265000
C	-5.733380000	2.313087000	-0.297139000
C	-2.320271000	0.675181000	-3.770587000

H	-1.786854000	-0.097000000	-3.233399000
C	-1.835625000	1.988711000	-3.962994000
C	-5.236919000	-1.240752000	-5.274364000
H	-4.543657000	-1.517742000	-6.073739000
H	-5.779496000	-2.147660000	-4.983678000
H	-5.964529000	-0.537859000	-5.672227000
C	-7.165406000	2.009177000	1.666841000
H	-6.442956000	2.546255000	2.287715000
H	-7.877477000	2.738573000	1.267173000
H	-7.712565000	1.304583000	2.304346000
C	-4.442123000	4.222279000	-0.610688000
C	-6.437848000	1.819219000	-5.791425000
H	-6.814108000	1.112240000	-6.537061000
H	-6.623682000	1.391375000	-4.805452000
H	-7.059905000	2.713883000	-5.872898000
C	-6.175773000	2.972610000	-1.467521000
H	-7.034021000	2.678110000	-2.054737000
C	-0.387007000	2.417071000	-3.808269000
C	-5.455572000	-0.433625000	-2.916885000
H	-6.088209000	-1.305316000	-2.714098000
H	-4.926954000	-0.257677000	-1.964287000
H	-6.117439000	0.411293000	-3.130263000
C	-2.876085000	2.702006000	-4.603749000
H	-2.784954000	3.712647000	-4.974750000
C	-5.413669000	4.167148000	-1.675374000
C	-4.642751000	3.061559000	0.187312000
H	-4.053915000	2.792537000	1.050578000
C	-4.725315000	5.852912000	-3.472738000
H	-4.073352000	6.459404000	-2.846219000
H	-4.107820000	5.081230000	-3.937919000
H	-5.116801000	6.504046000	-4.262295000
C	-5.452962000	0.273694000	1.134686000
H	-4.888304000	-0.249903000	0.361474000
H	-4.725170000	0.777237000	1.776210000
H	-5.982148000	-0.469481000	1.740604000
C	-2.157319000	5.127725000	-1.109000000
H	-1.744399000	4.111784000	-1.055050000
H	-2.408578000	5.346579000	-2.149097000
H	-1.351090000	5.804367000	-0.802567000
C	-4.468748000	-0.710520000	-4.059015000
C	0.413085000	1.735265000	-4.933127000
H	0.341526000	0.648817000	-4.847629000
H	0.027245000	2.022201000	-5.916674000
H	1.471096000	2.017937000	-4.878799000
C	-4.939773000	2.151686000	-6.030629000
C	-2.941150000	4.976468000	1.248771000

H	-3.797834000	4.998429000	1.929202000
H	-2.445983000	4.011028000	1.360416000
H	-2.248678000	5.761293000	1.565126000
C	-3.884970000	6.704361000	-0.187444000
H	-4.146857000	7.088718000	-1.170938000
H	-4.758373000	6.802567000	0.464168000
H	-3.096494000	7.351150000	0.212669000
C	-3.384078000	5.253309000	-0.197194000
C	-7.510430000	0.505900000	-0.281811000
H	-8.243079000	1.192766000	-0.718599000
H	-7.071406000	-0.082091000	-1.088608000
H	-8.054247000	-0.183383000	0.372846000
C	-4.409314000	1.349567000	-7.241337000
H	-3.361979000	1.605900000	-7.428443000
H	-4.465230000	0.274543000	-7.082416000
H	-4.989839000	1.594789000	-8.138810000
C	-5.877500000	5.211810000	-2.689341000
C	-4.839166000	3.624608000	-6.465915000
H	-5.085242000	4.319773000	-5.665152000
H	-3.838273000	3.865045000	-6.835228000
H	-5.540832000	3.799951000	-7.287883000
C	-0.223990000	3.931360000	-3.951981000
H	-0.553787000	4.276676000	-4.937198000
H	-0.791825000	4.472956000	-3.194132000
H	0.830415000	4.205920000	-3.841345000
C	0.179975000	1.951015000	-2.466013000
H	-0.374596000	2.374221000	-1.624202000
H	0.134577000	0.863347000	-2.379859000
H	1.227188000	2.255293000	-2.360268000
C	-6.815408000	4.550988000	-3.707537000
H	-6.362235000	3.656589000	-4.129952000
H	-7.769199000	4.265900000	-3.254003000
H	-7.036450000	5.246814000	-4.523038000
C	-6.719566000	6.288406000	-1.976942000
H	-7.558831000	5.817488000	-1.455584000
H	-6.151058000	6.857456000	-1.244374000
H	-7.127226000	6.992085000	-2.712544000

TS5

E= -4854.63511273 Ha

Sum of electronic and thermal Enthalpies= -4851.889432Ha

Sum of electronic and thermal Free Energies= -4852.201398 Ha

Imag_freq=-405.2 cm-1

Mo	-0.902615000	-1.562559000	1.199307000
O	-1.205414000	1.702507000	1.412197000
O	-2.533001000	0.475226000	-1.004823000

O	-0.500257000	-1.573132000	-2.004346000
O	-1.804074000	-0.573892000	3.993492000
O	1.526947000	0.512504000	1.817013000
O	1.830908000	-1.581797000	-0.532702000
C	-0.944037000	0.653129000	0.830830000
C	-1.443515000	-1.028289000	2.976153000
C	-1.417136000	0.103228000	-0.424923000
C	-0.395513000	-0.773303000	-0.954315000
C	0.643480000	-0.149705000	1.130137000
C	0.809693000	-0.824659000	-0.142925000
Y	0.950642000	-3.209011000	-1.701526000
C	0.370630000	-3.100358000	-5.375639000
C	-4.152315000	-3.189111000	2.082760000
H	-3.952082000	-3.908426000	2.883845000
H	-3.514009000	-2.315631000	2.236792000
H	-5.189964000	-2.857292000	2.181366000
C	-1.832883000	-4.965112000	-0.501185000
C	-2.490818000	-4.247432000	0.521236000
C	1.385112000	-3.425609000	-4.298801000
C	-1.491321000	-3.871201000	1.510252000
H	-1.743153000	-3.732003000	2.554919000
C	-0.226463000	-4.378815000	1.098997000
C	-4.961313000	-4.965657000	0.615511000
H	-4.728171000	-5.741978000	1.351270000
H	-5.961062000	-4.579025000	0.841767000
H	-5.012329000	-5.432280000	-0.366617000
C	0.990972000	-3.496049000	-6.727991000
H	1.924164000	-2.950341000	-6.898691000
H	1.216086000	-4.567073000	-6.755439000
H	0.302088000	-3.266765000	-7.549363000
C	3.310788000	-3.282682000	-2.992194000
C	-3.407609000	-5.300046000	-2.533244000
H	-4.328274000	-5.023198000	-2.023129000
H	-3.015740000	-4.408168000	-3.024607000
H	-3.669930000	-6.025271000	-3.311497000
C	1.685945000	-4.703304000	-3.778780000
H	1.161438000	-5.610213000	-4.042122000
C	0.965317000	-4.642862000	1.985497000
C	-4.272515000	-2.747010000	-0.356861000
H	-5.291008000	-2.373672000	-0.217697000
H	-3.583373000	-1.899052000	-0.278273000
H	-4.180045000	-3.136629000	-1.368586000
C	-0.427584000	-4.975388000	-0.164791000
H	0.284611000	-5.671737000	-0.603539000
C	2.873212000	-4.660023000	-2.984597000
C	2.372519000	-2.559340000	-3.783795000

H	2.411233000	-1.495446000	-3.963242000
C	4.570594000	-6.000977000	-1.497314000
H	5.427440000	-5.358096000	-1.692514000
H	4.123526000	-5.702626000	-0.546179000
H	4.953292000	-7.019741000	-1.371692000
C	0.056102000	-1.602992000	-5.384322000
H	-0.307635000	-1.274889000	-4.407615000
H	0.945384000	-1.016854000	-5.635086000
H	-0.708531000	-1.372222000	-6.133155000
C	4.796621000	-2.745750000	-0.979193000
H	4.036830000	-2.168606000	-0.449096000
H	4.747265000	-3.774719000	-0.634294000
H	5.782304000	-2.353031000	-0.705933000
C	-3.944573000	-3.815062000	0.695928000
C	0.803992000	-6.057441000	2.576868000
H	-0.128993000	-6.134925000	3.143045000
H	0.780801000	-6.811369000	1.783825000
H	1.636815000	-6.289028000	3.250899000
C	-2.350837000	-5.901563000	-1.600703000
C	4.523305000	-1.088001000	-2.756171000
H	4.474955000	-0.859547000	-3.825323000
H	3.664769000	-0.634941000	-2.252547000
H	5.430126000	-0.620369000	-2.360900000
C	5.789586000	-3.142959000	-3.279151000
H	5.970647000	-4.203135000	-3.098397000
H	5.628549000	-3.012072000	-4.354092000
H	6.698122000	-2.597122000	-2.999777000
C	4.584909000	-2.601420000	-2.490213000
C	-0.918642000	-3.897150000	-5.169144000
H	-0.736897000	-4.975252000	-5.205267000
H	-1.375173000	-3.659118000	-4.204641000
H	-1.649837000	-3.657398000	-5.948161000
C	-2.885266000	-7.187186000	-0.934694000
H	-2.101186000	-7.644288000	-0.322545000
H	-3.740578000	-6.996646000	-0.288976000
H	-3.187830000	-7.911162000	-1.700865000
C	3.544217000	-5.999101000	-2.634566000
C	-1.198054000	-6.352352000	-2.508403000
H	-0.675806000	-5.500582000	-2.948097000
H	-0.468210000	-6.967091000	-1.977328000
H	-1.590980000	-6.953911000	-3.334435000
C	2.241249000	-4.605338000	1.143757000
H	2.178403000	-5.325994000	0.321414000
H	2.419350000	-3.602169000	0.741564000
H	3.121045000	-4.879888000	1.731509000
C	1.024297000	-3.634926000	3.133409000

H	1.080115000	-2.606157000	2.753822000
H	0.129521000	-3.702032000	3.758438000
H	1.890637000	-3.819119000	3.774292000
C	2.488704000	-7.048120000	-2.242975000
H	1.951906000	-6.750025000	-1.336497000
H	1.752973000	-7.228051000	-3.028961000
H	2.979114000	-8.004023000	-2.032359000
C	4.243245000	-6.490590000	-3.919654000
H	3.520780000	-6.598843000	-4.733890000
H	5.009860000	-5.784476000	-4.248332000
H	4.721328000	-7.462335000	-3.749547000
Y	3.075872000	1.578079000	2.957901000
C	5.582554000	3.018317000	0.233072000
C	4.120160000	-2.435884000	2.474782000
H	3.895309000	-3.096944000	3.315195000
H	3.185000000	-2.034755000	2.079479000
H	4.580702000	-3.033302000	1.686239000
C	4.986905000	0.619264000	4.763837000
C	4.454993000	-0.483294000	4.003053000
C	4.531778000	3.184300000	1.324978000
C	3.079281000	-0.609238000	4.350729000
H	2.396109000	-1.336555000	3.941388000
C	2.726918000	0.389658000	5.284652000
C	6.393720000	-2.047051000	3.238176000
H	6.243393000	-2.727787000	4.081849000
H	6.701606000	-2.650169000	2.376728000
H	7.220742000	-1.384532000	3.480892000
C	5.646387000	4.394132000	-0.468363000
H	4.676695000	4.654108000	-0.902241000
H	5.916194000	5.178358000	0.245812000
H	6.394305000	4.382273000	-1.269914000
C	2.508850000	4.081141000	2.064876000
C	7.379991000	1.193273000	3.979367000
H	7.406225000	0.397942000	3.237360000
H	7.073838000	2.108031000	3.468447000
H	8.403945000	1.338524000	4.341522000
C	4.707378000	3.698120000	2.631806000
H	5.651922000	3.744546000	3.155327000
C	1.357198000	0.622002000	5.891594000
C	5.257488000	-0.408343000	1.677500000
H	5.838619000	-0.876897000	0.877969000
H	4.274048000	-0.187532000	1.230892000
H	5.750197000	0.527500000	1.942400000
C	3.893972000	1.154593000	5.506145000
H	3.974122000	1.949941000	6.231079000
C	3.488609000	4.279922000	3.109919000

C	3.163100000	3.371776000	1.023364000
H	2.701680000	3.088768000	0.090091000
C	2.230452000	5.420615000	5.083850000
H	1.409796000	5.836928000	4.504052000
H	1.908638000	4.457454000	5.493936000
H	2.404838000	6.095639000	5.928844000
C	5.179674000	1.972124000	-0.811575000
H	5.143169000	0.966535000	-0.392887000
H	4.197155000	2.188352000	-1.240523000
H	5.902269000	1.966228000	-1.634847000
C	0.078609000	4.147097000	2.873981000
H	-0.153776000	3.094201000	2.706378000
H	0.421975000	4.279562000	3.896103000
H	-0.857652000	4.701107000	2.773023000
C	5.081461000	-1.327769000	2.893136000
C	1.484457000	1.342096000	7.238027000
H	2.103954000	0.769261000	7.935352000
H	1.931870000	2.335320000	7.122200000
H	0.494964000	1.477811000	7.686980000
C	6.439994000	0.915485000	5.157693000
C	0.607268000	4.203459000	0.457943000
H	1.255234000	4.584396000	-0.337271000
H	0.535426000	3.117524000	0.385006000
H	-0.388084000	4.608608000	0.286304000
C	1.131599000	6.179351000	1.806383000
H	1.316167000	6.627748000	2.782043000
H	1.900315000	6.536440000	1.113125000
H	0.162566000	6.551926000	1.457158000
C	1.101047000	4.641257000	1.842274000
C	6.983903000	2.713336000	0.764133000
H	7.294706000	3.449538000	1.511902000
H	7.056987000	1.721412000	1.210446000
H	7.705955000	2.756003000	-0.058244000
C	6.935060000	-0.269706000	6.012927000
H	6.312313000	-0.358635000	6.908756000
H	6.891792000	-1.221112000	5.488727000
H	7.969987000	-0.098826000	6.332427000
C	3.529151000	5.269974000	4.285217000
C	6.552424000	2.146665000	6.064921000
H	6.255419000	3.054193000	5.544970000
H	5.953201000	2.049386000	6.974398000
H	7.596038000	2.266426000	6.374135000
C	0.551342000	1.505104000	4.931436000
H	1.063193000	2.468941000	4.792876000
H	0.402383000	1.026509000	3.958255000
H	-0.446308000	1.743504000	5.314326000

C	0.647648000	-0.719781000	6.097370000
H	0.517966000	-1.250539000	5.152085000
H	1.223616000	-1.357404000	6.775946000
H	-0.349571000	-0.565244000	6.519649000
C	4.591336000	4.869632000	5.311420000
H	4.368807000	3.892722000	5.740054000
H	5.593845000	4.844501000	4.878650000
H	4.612990000	5.601775000	6.125204000
C	3.965512000	6.639431000	3.721973000
H	4.957566000	6.554818000	3.267471000
H	3.283573000	7.013615000	2.959480000
H	4.019975000	7.380754000	4.528054000
Y	-3.782002000	1.947542000	-1.977118000
C	-5.800402000	0.957129000	1.101603000
C	-3.380513000	-1.729443000	-3.763619000
H	-2.627788000	-1.822641000	-4.547176000
H	-2.868323000	-1.511587000	-2.824665000
H	-3.884312000	-2.694052000	-3.663409000
C	-4.242959000	1.969778000	-4.751327000
C	-3.726721000	0.686932000	-4.323189000
C	-5.387101000	2.063870000	0.156727000
C	-2.387757000	0.894230000	-3.900589000
H	-1.738394000	0.130075000	-3.499775000
C	-2.053256000	2.263971000	-3.998137000
C	-5.126227000	-1.221107000	-5.391679000
H	-4.405507000	-1.375212000	-6.200533000
H	-5.561284000	-2.197884000	-5.151024000
H	-5.928265000	-0.588557000	-5.763079000
C	-6.584095000	1.629540000	2.247054000
H	-5.950578000	2.347915000	2.775420000
H	-7.457099000	2.165286000	1.859984000
H	-6.928704000	0.878789000	2.967941000
C	-4.470077000	4.119130000	-0.433241000
C	-6.704663000	1.684566000	-5.641596000
H	-7.053910000	1.152733000	-6.532645000
H	-6.772435000	0.997846000	-4.799220000
H	-7.420864000	2.487381000	-5.455751000
C	-6.129070000	2.601949000	-0.919559000
H	-7.029345000	2.163070000	-1.325934000
C	-0.656074000	2.830786000	-3.826439000
C	-5.401976000	-0.558266000	-2.972093000
H	-5.961942000	-1.488060000	-2.820635000
H	-4.849953000	-0.417106000	-2.028302000
H	-6.129320000	0.250786000	-3.093406000
C	-3.209024000	2.918639000	-4.488654000
H	-3.250872000	3.963016000	-4.763482000

C	-5.605057000	3.880880000	-1.286921000
C	-4.341823000	2.972487000	0.406245000
H	-3.578001000	2.826488000	1.154932000
C	-5.555375000	5.700110000	-3.100507000
H	-5.030883000	6.470007000	-2.537458000
H	-4.808587000	5.116698000	-3.643121000
H	-6.189323000	6.211713000	-3.833075000
C	-4.575876000	0.259875000	1.694130000
H	-3.972634000	-0.213419000	0.918271000
H	-3.926199000	0.963539000	2.219702000
H	-4.887196000	-0.499771000	2.415031000
C	-2.598350000	5.582830000	-1.324761000
H	-1.922204000	4.726192000	-1.402521000
H	-3.091138000	5.712552000	-2.288707000
H	-1.977192000	6.466274000	-1.135999000
C	-4.416135000	-0.670424000	-4.147838000
C	0.270834000	2.121144000	-4.828401000
H	0.318770000	1.049972000	-4.620667000
H	-0.090837000	2.251154000	-5.853252000
H	1.286890000	2.528871000	-4.765004000
C	-5.275887000	2.245360000	-5.859737000
C	-2.786375000	5.137961000	1.099984000
H	-3.442521000	5.046110000	1.971005000
H	-2.168941000	4.241233000	1.050367000
H	-2.128004000	5.997331000	1.263011000
C	-4.423473000	6.623474000	0.071543000
H	-4.951212000	6.986567000	-0.808383000
H	-5.158359000	6.446261000	0.862530000
H	-3.757013000	7.427013000	0.404438000
C	-3.596642000	5.353741000	-0.184436000
C	-6.727261000	-0.053976000	0.422098000
H	-7.656435000	0.419256000	0.086561000
H	-6.254556000	-0.518102000	-0.444854000
H	-6.996972000	-0.850382000	1.123725000
C	-4.660186000	1.632815000	-7.140153000
H	-3.666578000	2.057250000	-7.314368000
H	-4.551531000	0.551757000	-7.070226000
H	-5.290072000	1.861173000	-8.008351000
C	-6.411157000	4.790538000	-2.211848000
C	-5.414292000	3.745897000	-6.162942000
H	-5.773856000	4.310373000	-5.304623000
H	-4.469056000	4.183770000	-6.495991000
H	-6.137769000	3.877131000	-6.974638000
C	-0.624674000	4.332254000	-4.122099000
H	-0.904448000	4.531462000	-5.161755000
H	-1.304467000	4.890789000	-3.476283000

H	0.385620000	4.724050000	-3.964840000
C	-0.131499000	2.554846000	-2.413598000
H	-0.763440000	3.008103000	-1.642296000
H	-0.088979000	1.482218000	-2.213968000
H	0.876747000	2.961990000	-2.282695000
C	-7.273534000	3.924549000	-3.139514000
H	-6.664054000	3.166630000	-3.630863000
H	-8.075889000	3.417134000	-2.597037000
H	-7.744798000	4.545953000	-3.907914000
C	-7.385677000	5.638828000	-1.370550000
H	-8.024481000	4.987159000	-0.766439000
H	-6.870102000	6.319865000	-0.694234000
H	-8.029248000	6.234727000	-2.028716000

Int8

E= -4854.69111918 Ha

Sum of electronic and thermal Enthalpies= -4851.944708 Ha

Sum of electronic and thermal Free Energies= -4852.249895 Ha

Mo	-0.789455000	-1.903188000	0.796767000
O	-0.895626000	1.861745000	1.367647000
O	-2.390789000	0.510200000	-0.798169000
O	-0.500550000	-1.906572000	-1.590558000
O	-1.262869000	-0.928246000	3.691785000
O	1.749666000	0.629836000	1.738931000
O	1.978028000	-1.609573000	-0.401107000
C	-0.496044000	0.877057000	0.738751000
C	-1.067061000	-1.324265000	2.602232000
C	-1.221909000	0.135796000	-0.322350000
C	-0.260394000	-0.781575000	-0.897605000
C	0.849472000	0.209199000	0.918605000
C	0.963952000	-0.804048000	-0.085531000
Y	1.193998000	-3.387122000	-1.433488000
C	0.729174000	-2.886115000	-5.051453000
C	-4.066395000	-3.435819000	1.924413000
H	-3.877158000	-4.192548000	2.693140000
H	-3.404373000	-2.585307000	2.102991000
H	-5.093567000	-3.080244000	2.048436000
C	-1.817809000	-5.208840000	-0.745087000
C	-2.453061000	-4.472915000	0.294164000
C	1.696104000	-3.403333000	-4.006324000
C	-1.439575000	-4.137676000	1.264202000
H	-1.664300000	-3.922927000	2.301458000
C	-0.186397000	-4.676404000	0.846588000
C	-4.934179000	-5.137799000	0.396072000
H	-4.701275000	-5.953464000	1.087861000
H	-5.921094000	-4.743862000	0.662148000

H	-5.013902000	-5.552924000	-0.607512000
C	1.379472000	-3.099849000	-6.430392000
H	2.330433000	-2.562253000	-6.495515000
H	1.578428000	-4.161953000	-6.605349000
H	0.721187000	-2.735542000	-7.227635000
C	3.598812000	-3.550451000	-2.667936000
C	-3.397027000	-5.394118000	-2.785504000
H	-4.286590000	-5.062083000	-2.253188000
H	-2.951599000	-4.524800000	-3.273654000
H	-3.723350000	-6.087783000	-3.568490000
C	1.874891000	-4.746549000	-3.604131000
H	1.275036000	-5.575773000	-3.947981000
C	0.973977000	-5.057609000	1.740898000
C	-4.223755000	-2.894591000	-0.486701000
H	-5.253374000	-2.552798000	-0.347793000
H	-3.555548000	-2.037549000	-0.342601000
H	-4.100623000	-3.221985000	-1.516468000
C	-0.428427000	-5.285287000	-0.410025000
H	0.264026000	-5.973995000	-0.887221000
C	3.044934000	-4.878655000	-2.794044000
C	2.748075000	-2.679132000	-3.405229000
H	2.887394000	-1.611631000	-3.486763000
C	4.588362000	-6.499982000	-1.412651000
H	5.490412000	-5.897660000	-1.502600000
H	4.128856000	-6.292715000	-0.443567000
H	4.901305000	-7.549549000	-1.402870000
C	0.460874000	-1.392370000	-4.851320000
H	0.030438000	-1.201368000	-3.865097000
H	1.382723000	-0.810786000	-4.945785000
H	-0.239697000	-1.022269000	-5.606694000
C	4.991763000	-3.318361000	-0.543762000
H	4.240414000	-2.726327000	-0.016820000
H	4.843795000	-4.366111000	-0.295877000
H	5.977029000	-3.022854000	-0.165184000
C	-3.893773000	-4.011643000	0.509738000
C	0.873847000	-6.562810000	2.049391000
H	-0.093209000	-6.795081000	2.505113000
H	0.967010000	-7.160538000	1.137597000
H	1.665666000	-6.867675000	2.743624000
C	-2.369985000	-6.081440000	-1.879534000
C	4.964375000	-1.495920000	-2.172727000
H	5.010191000	-1.171885000	-3.217070000
H	4.105088000	-1.024702000	-1.687724000
H	5.872207000	-1.136577000	-1.677669000
C	6.104819000	-3.590528000	-2.810909000
H	6.197528000	-4.673799000	-2.727540000

H	6.024864000	-3.345547000	-3.874883000
H	7.030687000	-3.146467000	-2.427514000
C	4.894982000	-3.027153000	-2.046141000
C	-0.591381000	-3.656489000	-4.999219000
H	-0.443102000	-4.721632000	-5.199365000
H	-1.069420000	-3.552042000	-4.020523000
H	-1.288932000	-3.275764000	-5.752188000
C	-2.963290000	-7.366544000	-1.264528000
H	-2.201751000	-7.879098000	-0.668185000
H	-3.813068000	-7.166482000	-0.614624000
H	-3.292200000	-8.046991000	-2.059002000
C	3.606311000	-6.294363000	-2.570999000
C	-1.234436000	-6.546892000	-2.803852000
H	-0.674094000	-5.699334000	-3.198870000
H	-0.537819000	-7.216492000	-2.293490000
H	-1.653377000	-7.099324000	-3.651221000
C	2.277855000	-4.791230000	0.992355000
H	2.298995000	-5.328988000	0.031488000
H	2.424535000	-3.714710000	0.840642000
H	3.157214000	-5.143436000	1.540038000
C	0.934766000	-4.284436000	3.056951000
H	0.921611000	-3.204477000	2.882848000
H	0.036170000	-4.539978000	3.626267000
H	1.801858000	-4.534016000	3.674625000
C	2.466484000	-7.300659000	-2.332270000
H	1.920917000	-7.072619000	-1.410669000
H	1.748805000	-7.331972000	-3.153533000
H	2.881140000	-8.308217000	-2.224645000
C	4.316782000	-6.689387000	-3.882933000
H	3.616795000	-6.657154000	-4.722835000
H	5.137400000	-6.004030000	-4.108446000
H	4.725109000	-7.704171000	-3.810228000
Y	3.087973000	1.822582000	2.997637000
C	5.518704000	2.892076000	0.148868000
C	3.586536000	-2.328333000	2.811208000
H	3.261928000	-2.849182000	3.714450000
H	2.721185000	-1.858414000	2.336949000
H	3.983590000	-3.071038000	2.119306000
C	4.845459000	0.763537000	4.845927000
C	4.177068000	-0.318362000	4.166515000
C	4.532181000	3.249277000	1.251648000
C	2.800359000	-0.253858000	4.522751000
H	2.027444000	-0.912201000	4.153309000
C	2.581091000	0.835523000	5.393929000
C	5.892404000	-2.169019000	3.568072000
H	5.652709000	-2.735773000	4.473414000

H	6.121312000	-2.890265000	2.775448000
H	6.798566000	-1.597743000	3.755788000
C	5.716064000	4.192527000	-0.660629000
H	4.766752000	4.535993000	-1.081637000
H	6.108578000	4.989136000	-0.020748000
H	6.421718000	4.029248000	-1.483784000
C	2.623023000	4.352441000	2.018592000
C	7.272392000	0.986939000	3.988327000
H	7.207863000	0.119799000	3.334208000
H	7.053357000	1.872343000	3.387300000
H	8.310736000	1.064201000	4.329545000
C	4.806398000	3.812639000	2.522142000
H	5.775473000	3.813897000	3.002291000
C	1.243322000	1.270630000	5.955934000
C	4.989794000	-0.545546000	1.840513000
H	5.547600000	-1.142212000	1.113313000
H	4.046900000	-0.271661000	1.348620000
H	5.573070000	0.355691000	2.039294000
C	3.832151000	1.466740000	5.565051000
H	4.014345000	2.294383000	6.233532000
C	3.658386000	4.514081000	3.015179000
C	3.173384000	3.540323000	0.990611000
H	2.649995000	3.246973000	0.093846000
C	2.547765000	5.798811000	4.976128000
H	1.749958000	6.268520000	4.405484000
H	2.159332000	4.869375000	5.404194000
H	2.789166000	6.469376000	5.808023000
C	4.963883000	1.818808000	-0.793160000
H	4.814204000	0.867398000	-0.282560000
H	4.005277000	2.119671000	-1.225940000
H	5.659797000	1.648832000	-1.621473000
C	0.214453000	4.672919000	2.863000000
H	-0.159115000	3.661306000	2.684576000
H	0.599309000	4.734839000	3.877675000
H	-0.635799000	5.355797000	2.799357000
C	4.687548000	-1.321060000	3.132924000
C	1.437705000	2.277641000	7.092168000
H	2.020278000	1.841689000	7.910063000
H	1.958115000	3.177834000	6.748783000
H	0.466635000	2.589083000	7.490492000
C	6.329992000	0.931876000	5.195237000
C	0.699656000	4.636644000	0.445404000
H	1.371370000	4.914995000	-0.372070000
H	0.496626000	3.565880000	0.408391000
H	-0.245023000	5.155811000	0.280467000
C	1.441023000	6.572209000	1.739589000

H	1.682842000	7.027556000	2.699029000
H	2.223969000	6.842193000	1.023535000
H	0.502039000	7.021839000	1.397799000
C	1.268761000	5.043946000	1.810020000
C	6.889732000	2.463575000	0.673704000
H	7.308697000	3.212063000	1.353733000
H	6.852161000	1.508134000	1.197595000
H	7.587073000	2.352674000	-0.163475000
C	6.710013000	-0.209166000	6.162290000
H	6.100740000	-0.138421000	7.068944000
H	6.551071000	-1.197685000	5.737492000
H	7.764132000	-0.124277000	6.452363000
C	3.813346000	5.531419000	4.155546000
C	6.593045000	2.230796000	5.967655000
H	6.388639000	3.105148000	5.353526000
H	6.002299000	2.299092000	6.885005000
H	7.648482000	2.266035000	6.257489000
C	0.463216000	1.935998000	4.817104000
H	1.025995000	2.802254000	4.434922000
H	0.253829000	1.246372000	3.994362000
H	-0.498619000	2.345195000	5.145298000
C	0.469378000	0.055624000	6.479841000
H	0.261807000	-0.660016000	5.681960000
H	1.035819000	-0.450844000	7.268010000
H	-0.494873000	0.370286000	6.893818000
C	4.867170000	5.073982000	5.165934000
H	4.581222000	4.127998000	5.624208000
H	5.852541000	4.958841000	4.708903000
H	4.968150000	5.821546000	5.959547000
C	4.344107000	6.846145000	3.544893000
H	5.317190000	6.670887000	3.075300000
H	3.679789000	7.252568000	2.783881000
H	4.474200000	7.602573000	4.327907000
Y	-3.681718000	1.946444000	-1.734941000
C	-5.491904000	0.790016000	1.408905000
C	-3.153343000	-1.641155000	-3.629423000
H	-2.381552000	-1.660008000	-4.399791000
H	-2.674316000	-1.438698000	-2.670529000
H	-3.597898000	-2.637187000	-3.578220000
C	-4.188135000	2.034786000	-4.514946000
C	-3.612683000	0.764420000	-4.129996000
C	-5.195387000	1.936783000	0.467114000
C	-2.278633000	1.017748000	-3.717836000
H	-1.589091000	0.269563000	-3.358011000
C	-2.001381000	2.401929000	-3.784928000
C	-4.904289000	-1.178835000	-5.263832000

H	-4.164672000	-1.278901000	-6.064298000
H	-5.296553000	-2.180336000	-5.053276000
H	-5.729068000	-0.575651000	-5.634238000
C	-6.196700000	1.402831000	2.635929000
H	-5.536288000	2.111934000	3.143598000
H	-7.105081000	1.937037000	2.338191000
H	-6.473191000	0.618062000	3.350047000
C	-4.443540000	4.048558000	-0.152969000
C	-6.636128000	1.651327000	-5.417471000
H	-6.974668000	1.179665000	-6.345891000
H	-6.658428000	0.893751000	-4.636495000
H	-7.385580000	2.400357000	-5.154976000
C	-6.018482000	2.428111000	-0.572543000
H	-6.905430000	1.931573000	-0.941387000
C	-0.620461000	3.013195000	-3.641276000
C	-5.246286000	-0.584881000	-2.832062000
H	-5.776804000	-1.536358000	-2.716075000
H	-4.717132000	-0.447097000	-1.874910000
H	-5.996029000	0.204323000	-2.943878000
C	-3.191332000	3.019143000	-4.237934000
H	-3.281311000	4.068637000	-4.480862000
C	-5.597631000	3.739671000	-0.956864000
C	-4.203436000	2.912511000	0.676679000
H	-3.389546000	2.813010000	1.379759000
C	-5.763222000	5.593190000	-2.730130000
H	-5.285852000	6.393901000	-2.168817000
H	-4.991219000	5.085105000	-3.311370000
H	-6.463491000	6.063404000	-3.429354000
C	-4.207976000	0.103916000	1.877530000
H	-3.696554000	-0.390633000	1.051379000
H	-3.499138000	0.817899000	2.303237000
H	-4.437288000	-0.637911000	2.647326000
C	-2.735802000	5.633411000	-1.152154000
H	-2.002410000	4.828284000	-1.263116000
H	-3.290534000	5.716109000	-2.086627000
H	-2.172432000	6.562621000	-1.004276000
C	-4.238445000	-0.626480000	-3.995144000
C	0.292364000	2.369960000	-4.699787000
H	0.373438000	1.292543000	-4.542211000
H	-0.104823000	2.534780000	-5.706385000
H	1.299606000	2.800698000	-4.650413000
C	-5.239885000	2.294980000	-5.610513000
C	-2.718832000	5.167911000	1.264797000
H	-3.293024000	5.015543000	2.183778000
H	-2.032265000	4.328063000	1.151803000
H	-2.124492000	6.078966000	1.387667000

C	-4.532911000	6.539562000	0.383305000
H	-5.158227000	6.865793000	-0.445344000
H	-5.186330000	6.301376000	1.228167000
H	-3.905158000	7.390347000	0.671435000
C	-3.638402000	5.334892000	0.050015000
C	-6.443173000	-0.222570000	0.767223000
H	-7.415618000	0.230094000	0.545733000
H	-6.036321000	-0.619520000	-0.164855000
H	-6.615787000	-1.064644000	1.445359000
C	-4.593817000	1.761795000	-6.911407000
H	-3.624402000	2.244068000	-7.071189000
H	-4.427535000	0.686179000	-6.877914000
H	-5.236486000	1.985277000	-7.771440000
C	-6.506510000	4.596175000	-1.835341000
C	-5.466859000	3.794128000	-5.857928000
H	-5.867279000	4.299840000	-4.981187000
H	-4.549729000	4.303836000	-6.166398000
H	-6.192611000	3.912213000	-6.669716000
C	-0.643971000	4.523098000	-3.891907000
H	-0.967078000	4.742570000	-4.914816000
H	-1.314150000	5.044993000	-3.207592000
H	0.360160000	4.939640000	-3.760727000
C	-0.042672000	2.707885000	-2.255885000
H	-0.668304000	3.111669000	-1.452947000
H	0.040808000	1.630144000	-2.095156000
H	0.955057000	3.144530000	-2.140818000
C	-7.321194000	3.680302000	-2.758307000
H	-6.663296000	2.994259000	-3.291804000
H	-8.055117000	3.087089000	-2.206127000
H	-7.873453000	4.275886000	-3.492575000
C	-7.515183000	5.340897000	-0.938431000
H	-8.090599000	4.622901000	-0.345767000
H	-7.026516000	6.026628000	-0.246307000
H	-8.215735000	5.917662000	-1.554295000

1_Y
E= -4673.17417933 Ha
Sum of electronic and thermal Enthalpies= -4670.440125Ha
Sum of electronic and thermal Free Energies= -4670.744303 Ha

C	-2.486626000	-5.176751000	7.619734000
C	-2.272386000	-6.673325000	7.893458000
C	-0.821319000	-7.157801000	7.890079000
H	-0.801539000	-8.248661000	7.987814000

H	-0.244634000	-6.752593000	8.721001000
H	-0.313589000	-6.897870000	6.958531000
C	-2.896904000	-6.984514000	9.270130000
H	-2.801620000	-8.052469000	9.499860000
H	-3.960184000	-6.726105000	9.273065000
H	-2.415964000	-6.419414000	10.070603000
C	-3.023900000	-7.528650000	6.858179000
H	-2.832469000	-8.589307000	7.050935000
H	-2.693025000	-7.302192000	5.844469000
H	-4.105984000	-7.384882000	6.900153000
C	-1.803839000	-3.974952000	8.048811000
C	-0.484887000	-3.737137000	8.804285000
C	-0.269472000	-2.230533000	9.024199000
H	0.687289000	-2.074572000	9.531206000
H	-1.049942000	-1.795931000	9.655115000
H	-0.236429000	-1.676723000	8.081857000
C	0.789300000	-4.245312000	8.112775000
H	1.648885000	-4.062014000	8.767383000
H	0.954824000	-3.723206000	7.171127000
H	0.766368000	-5.310731000	7.900698000
C	-0.596218000	-4.365275000	10.205375000
H	0.265506000	-4.072121000	10.816098000
H	-0.615498000	-5.455091000	10.167363000
H	-1.507129000	-4.026817000	10.709803000
C	-2.721887000	-2.901030000	7.855301000
H	-2.510413000	-1.862580000	8.065778000
C	-3.964521000	-3.385039000	7.378223000
C	-5.261500000	-2.601948000	7.287797000
C	-6.432983000	-3.519255000	6.926056000
H	-7.362255000	-2.940981000	6.886572000
H	-6.560716000	-4.312898000	7.669170000
H	-6.293754000	-3.988285000	5.949231000
C	-5.545511000	-1.991566000	8.671105000
H	-6.480338000	-1.419145000	8.655030000
H	-4.742795000	-1.319709000	8.983423000
H	-5.633811000	-2.778382000	9.426783000
C	-5.159868000	-1.482317000	6.248461000
H	-6.073498000	-0.878674000	6.237225000
H	-5.027091000	-1.889102000	5.239990000
H	-4.312260000	-0.825375000	6.443740000
C	-3.793424000	-4.769712000	7.204881000
H	-4.576481000	-5.452063000	6.913081000
C	-2.418649000	5.019808000	3.239011000
C	-2.028204000	6.461828000	2.893715000
C	-0.529604000	6.770784000	2.948059000
H	-0.367077000	7.838836000	2.769013000

H	0.040553000	6.227582000	2.196129000
H	-0.109698000	6.533132000	3.927332000
C	-2.554522000	6.756298000	1.474404000
H	-2.336219000	7.792933000	1.191396000
H	-3.638334000	6.610149000	1.436679000
H	-2.104036000	6.096591000	0.730893000
C	-2.725086000	7.448621000	3.848135000
H	-2.373685000	8.465828000	3.648316000
H	-2.513740000	7.212423000	4.893029000
H	-3.810013000	7.452698000	3.715807000
C	-1.892490000	3.731006000	2.837006000
C	-0.581028000	3.302791000	2.170152000
C	-0.588571000	1.787103000	1.934864000
H	0.347888000	1.488579000	1.456620000
H	-1.406779000	1.491377000	1.274086000
H	-0.675008000	1.230666000	2.871055000
C	0.643987000	3.590483000	3.046004000
H	1.549826000	3.245356000	2.539344000
H	0.584952000	3.041497000	3.992574000
H	0.777582000	4.649671000	3.257409000
C	-0.420520000	3.949325000	0.784823000
H	0.463487000	3.537608000	0.285140000
H	-0.296336000	5.030962000	0.827524000
H	-1.294018000	3.735118000	0.160801000
C	-2.899053000	2.771680000	3.128870000
H	-2.811333000	1.709264000	2.955090000
C	-4.049837000	3.403454000	3.654947000
C	-5.413487000	2.751520000	3.775832000
C	-6.462008000	3.742975000	4.284339000
H	-7.432934000	3.246662000	4.383624000
H	-6.582722000	4.579148000	3.587991000
H	-6.195856000	4.154238000	5.259588000
C	-5.830478000	2.297606000	2.364205000
H	-6.829753000	1.847725000	2.385860000
H	-5.132559000	1.556873000	1.966256000
H	-5.849286000	3.148065000	1.675419000
C	-5.357298000	1.525517000	4.690655000
H	-6.321646000	1.006828000	4.703296000
H	-5.121047000	1.804771000	5.720751000
H	-4.598643000	0.812089000	4.360720000
C	-3.732971000	4.773797000	3.744717000
H	-4.421476000	5.544530000	4.056121000
Y	-2.070050000	3.588719000	5.483865000
C	-3.140133000	4.587370000	7.730125000
C	-4.587770000	4.956231000	8.077051000
C	-5.598558000	3.808210000	8.014042000

H	-6.604553000	4.193836000	8.211024000
H	-5.402800000	3.030095000	8.749981000
H	-5.613175000	3.343789000	7.026098000
C	-4.586692000	5.550061000	9.500249000
H	-5.595218000	5.877381000	9.779278000
H	-3.918711000	6.415450000	9.548860000
H	-4.245283000	4.824950000	10.241052000
C	-5.099379000	6.054070000	7.126403000
H	-6.161323000	6.240820000	7.314506000
H	-4.982361000	5.761528000	6.080696000
H	-4.574867000	7.001717000	7.272722000
C	-2.276524000	3.496655000	8.133208000
C	-2.549902000	2.141220000	8.791057000
C	-1.226423000	1.401727000	9.022950000
H	-1.426816000	0.433971000	9.490032000
H	-0.569185000	1.963067000	9.691112000
H	-0.696017000	1.215399000	8.086303000
C	-3.400907000	1.223710000	7.905620000
H	-3.534724000	0.256227000	8.397527000
H	-2.898095000	1.024788000	6.952745000
H	-4.393153000	1.623864000	7.706381000
C	-3.194243000	2.307966000	10.176543000
H	-3.271780000	1.331551000	10.667584000
H	-4.196895000	2.732918000	10.135886000
H	-2.578538000	2.957080000	10.807266000
C	-0.946042000	3.901723000	7.842000000
H	-0.064062000	3.302526000	8.015155000
C	-0.930569000	5.214132000	7.315465000
C	0.308751000	6.077887000	7.185384000
C	-0.036873000	7.478945000	6.677424000
H	0.873863000	8.079011000	6.580006000
H	-0.705397000	7.995517000	7.373729000
H	-0.523876000	7.452600000	5.701079000
C	0.922416000	6.218089000	8.591131000
H	1.810904000	6.859081000	8.558669000
H	1.219890000	5.245311000	8.990512000
H	0.200980000	6.661506000	9.284640000
C	1.336276000	5.419456000	6.261977000
H	2.260674000	6.005468000	6.226927000
H	0.958186000	5.340053000	5.239531000
H	1.591242000	4.411537000	6.597576000
C	-2.279415000	5.610995000	7.224334000
H	-2.611662000	6.590163000	6.915289000
Y	-2.229119000	-3.609704000	5.369908000
C	-3.487192000	-4.600827000	3.185767000
C	-4.955897000	-4.979511000	2.951014000

C	-5.952954000	-3.818692000	2.976203000
H	-6.971756000	-4.208138000	2.875430000
H	-5.796644000	-3.113252000	2.161200000
H	-5.901623000	-3.269060000	3.919371000
C	-5.048512000	-5.694334000	1.587176000
H	-6.075536000	-6.030424000	1.400277000
H	-4.392815000	-6.570214000	1.574240000
H	-4.748126000	-5.043266000	0.764790000
C	-5.424013000	-5.987424000	4.016345000
H	-6.493233000	-6.187687000	3.892385000
H	-5.260576000	-5.599980000	5.022890000
H	-4.905159000	-6.945757000	3.937688000
C	-2.641693000	-3.529017000	2.700957000
C	-2.933798000	-2.250712000	1.904368000
C	-1.616350000	-1.533471000	1.575329000
H	-1.834684000	-0.624711000	1.009128000
H	-0.957007000	-2.153619000	0.961865000
H	-1.075480000	-1.240627000	2.479548000
C	-3.800489000	-1.224449000	2.647298000
H	-3.991837000	-0.367043000	1.992111000
H	-3.282234000	-0.863536000	3.538736000
H	-4.766863000	-1.620819000	2.953334000
C	-3.578108000	-2.607918000	0.554178000
H	-3.642766000	-1.713729000	-0.076578000
H	-4.588107000	-3.003345000	0.664215000
H	-2.976831000	-3.355728000	0.027563000
C	-1.298128000	-3.947376000	2.913963000
H	-0.420726000	-3.363907000	2.677731000
C	-1.256784000	-5.249376000	3.461217000
C	-0.025085000	-6.139205000	3.538158000
C	-0.412830000	-7.568850000	3.925747000
H	0.476475000	-8.207723000	3.935021000
H	-1.128068000	-7.995848000	3.215027000
H	-0.857089000	-7.609845000	4.923030000
C	0.600651000	-6.174862000	2.131449000
H	1.472081000	-6.839584000	2.117751000
H	0.931377000	-5.179222000	1.822608000
H	-0.122189000	-6.534850000	1.392221000
C	1.024249000	-5.617492000	4.526064000
H	1.907582000	-6.266299000	4.515777000
H	0.633791000	-5.594251000	5.546372000
H	1.336050000	-4.600625000	4.285569000
C	-2.602189000	-5.621983000	3.653344000
H	-2.929354000	-6.589999000	3.997946000
C	5.723272000	-0.422802000	3.366120000
C	6.909199000	-1.361839000	3.097077000

C	6.597806000	-2.859559000	3.093599000
H	7.530979000	-3.424092000	2.992062000
H	5.956114000	-3.149223000	2.262031000
H	6.120096000	-3.173342000	4.024865000
C	7.501169000	-0.973828000	1.725785000
H	8.371150000	-1.599671000	1.493511000
H	7.822489000	0.072188000	1.731865000
H	6.771863000	-1.091119000	0.922061000
C	8.016292000	-1.146079000	4.143655000
H	8.837525000	-1.846668000	3.960651000
H	7.639004000	-1.317177000	5.152175000
H	8.439019000	-0.139487000	4.107948000
C	4.340004000	-0.410764000	2.942409000
C	3.469386000	-1.432346000	2.189492000
C	2.059094000	-0.860542000	1.968112000
H	1.446587000	-1.604910000	1.451163000
H	2.078245000	0.037753000	1.344965000
H	1.555661000	-0.618676000	2.908353000
C	3.270678000	-2.790194000	2.879909000
H	2.670028000	-3.437553000	2.231022000
H	2.749660000	-2.673464000	3.829238000
H	4.204734000	-3.309230000	3.076719000
C	4.067610000	-1.652347000	0.787592000
H	3.382660000	-2.253470000	0.178597000
H	5.021682000	-2.179965000	0.824375000
H	4.229159000	-0.695756000	0.280019000
C	3.872981000	0.922369000	3.135731000
H	2.867618000	1.260863000	2.932193000
C	4.917742000	1.755157000	3.605933000
C	4.887222000	3.269286000	3.697474000
C	6.262025000	3.826486000	4.075605000
H	6.221524000	4.919844000	4.123607000
H	7.020401000	3.549115000	3.336508000
H	6.591861000	3.464943000	5.052228000
C	4.516841000	3.822760000	2.310787000
H	4.481908000	4.918311000	2.330529000
H	3.540732000	3.458830000	1.982042000
H	5.255720000	3.513294000	1.564894000
C	3.854361000	3.738420000	4.725923000
H	3.788112000	4.831427000	4.736815000
H	4.129418000	3.418752000	5.736948000
H	2.863533000	3.331587000	4.522615000
C	6.030609000	0.912735000	3.776234000
H	7.014634000	1.245029000	4.067926000
O	-0.071709000	-2.968579000	5.441898000
C	0.198705000	-1.719205000	5.463111000

O	-2.089015000	-1.135668000	5.335090000
C	-0.899928000	-0.716784000	5.416485000
O	-1.008283000	1.746605000	5.478145000
C	-0.352244000	0.618757000	5.482910000
O	2.033379000	1.258284000	5.630602000
C	1.079319000	0.433739000	5.557008000
O	2.623919000	-1.399293000	5.554039000
C	1.404426000	-1.017770000	5.525184000
Y	4.254168000	0.140570000	5.617830000
C	5.732906000	0.721346000	7.806146000
C	6.794499000	1.805437000	8.036551000
C	6.283377000	3.248463000	8.007017000
H	7.128693000	3.938798000	8.100662000
H	5.596998000	3.468739000	8.823637000
H	5.776633000	3.473456000	7.065178000
C	7.462810000	1.533618000	9.399894000
H	8.262813000	2.260216000	9.585880000
H	7.899865000	0.530581000	9.411745000
H	6.749244000	1.593978000	10.223047000
C	7.899231000	1.706559000	6.969308000
H	8.609568000	2.530734000	7.092117000
H	7.480942000	1.760207000	5.963132000
H	8.468305000	0.777048000	7.046504000
C	4.380785000	0.529873000	8.289711000
C	3.422465000	1.423880000	9.088165000
C	2.149074000	0.638458000	9.435435000
H	1.479190000	1.279587000	10.013483000
H	2.370085000	-0.242026000	10.045066000
H	1.608906000	0.314722000	8.541420000
C	2.958211000	2.680276000	8.338286000
H	2.300310000	3.270948000	8.986013000
H	2.396485000	2.404274000	7.442852000
H	3.781053000	3.325906000	8.036253000
C	4.060012000	1.815775000	10.432127000
H	3.320015000	2.323844000	11.061212000
H	4.906738000	2.491794000	10.313520000
H	4.409325000	0.926232000	10.965742000
C	4.066585000	-0.841285000	8.075365000
H	3.119473000	-1.305036000	8.308267000
C	5.171906000	-1.532519000	7.530237000
C	5.320703000	-3.045272000	7.455257000
C	6.753377000	-3.430873000	7.077144000
H	6.858557000	-4.520804000	7.077550000
H	7.478374000	-3.021027000	7.788009000
H	7.015541000	-3.074947000	6.077944000
C	5.026632000	-3.601465000	8.860901000

H	5.158298000	-4.689566000	8.876273000
H	3.998774000	-3.381843000	9.163213000
H	5.698903000	-3.160122000	9.603814000
C	4.348509000	-3.692618000	6.462457000
H	4.456790000	-4.783021000	6.486273000
H	4.540617000	-3.357287000	5.440216000
H	3.311824000	-3.440384000	6.687356000
C	6.171903000	-0.557815000	7.341321000
H	7.174340000	-0.764116000	7.000758000

Int4’

E= -3485.77773364 Ha

Sum of electronic and thermal Enthalpies= -3483.927339Ha

Sum of electronic and thermal Free Energies= -3484.156043 Ha

Mo	-2.651335000	0.658553000	0.385406000
O	-3.073182000	3.571806000	-0.800072000
O	-4.268571000	1.060138000	-2.554711000
O	-2.572211000	-1.573342000	-2.617339000
O	-2.506646000	-1.678543000	2.562275000
O	-0.696157000	2.161914000	2.247804000
O	-0.458199000	-1.183271000	-0.946208000
C	-2.888525000	2.489943000	-0.428798000
C	-2.575749000	-0.887804000	1.720987000
C	-3.449934000	0.521018000	-1.868031000
C	-2.422783000	-0.473149000	-1.962130000
C	-1.491047000	1.532325000	1.572602000
C	-1.424751000	-0.360195000	-0.893009000
Y	-0.870253000	-3.023293000	-2.493823000
C	-1.693999000	-1.883245000	-5.905444000
C	-4.611297000	-2.917204000	-0.189861000
H	-4.346914000	-3.320260000	0.791823000
H	-3.977023000	-2.050692000	-0.394832000
H	-5.646801000	-2.565849000	-0.135419000
C	-2.383306000	-5.365878000	-2.328008000
C	-3.033137000	-4.433399000	-1.439466000
C	-0.530522000	-2.239747000	-5.000397000
C	-2.088742000	-4.117279000	-0.419028000
H	-2.268451000	-3.437395000	0.399390000
C	-0.893403000	-4.847777000	-0.606630000
C	-5.341692000	-5.172407000	-0.860623000
H	-4.926241000	-5.617015000	0.049761000
H	-6.368060000	-4.851057000	-0.648661000
H	-5.388990000	-5.953239000	-1.620723000
C	-1.196547000	-2.000708000	-7.358048000
H	-0.352138000	-1.326225000	-7.532510000
H	-0.863996000	-3.020066000	-7.578889000

H	-1.996789000	-1.741112000	-8.060971000
C	1.400164000	-2.057752000	-3.709190000
C	-4.164109000	-6.029764000	-4.140497000
H	-5.044280000	-5.924170000	-3.507710000
H	-4.032441000	-5.110251000	-4.713325000
H	-4.375795000	-6.834905000	-4.852889000
C	0.090971000	-3.504701000	-4.916485000
H	-0.226162000	-4.376282000	-5.469206000
C	0.229470000	-5.015303000	0.393991000
C	-5.104296000	-3.311276000	-2.553181000
H	-6.159622000	-3.087391000	-2.358814000
H	-4.590223000	-2.376492000	-2.777929000
H	-5.061109000	-3.939782000	-3.436830000
C	-1.069646000	-5.577170000	-1.800244000
H	-0.364547000	-6.295425000	-2.191852000
C	1.309180000	-3.421767000	-4.167282000
C	0.258015000	-1.370510000	-4.221735000
H	0.023843000	-0.333039000	-4.033503000
C	3.499258000	-4.662907000	-3.382993000
H	4.119104000	-3.771656000	-3.308322000
H	3.131756000	-4.916606000	-2.388080000
H	4.154153000	-5.480470000	-3.703991000
C	-2.177449000	-0.450848000	-5.663622000
H	-2.568365000	-0.331716000	-4.652297000
H	-1.368011000	0.270276000	-5.818685000
H	-2.979599000	-0.205641000	-6.367699000
C	2.855823000	-1.908122000	-1.612533000
H	1.973974000	-1.919634000	-0.965655000
H	3.234300000	-2.924178000	-1.690717000
H	3.622600000	-1.305580000	-1.111594000
C	-4.490982000	-3.967591000	-1.306492000
C	-0.110399000	-6.221167000	1.287112000
H	-1.061028000	-6.059506000	1.804285000
H	-0.203481000	-7.131941000	0.687346000
H	0.670665000	-6.380586000	2.040273000
C	-2.902586000	-6.390835000	-3.353047000
C	2.049300000	0.145761000	-2.688781000
H	1.880732000	0.707173000	-3.612610000
H	1.136717000	0.166135000	-2.092042000
H	2.834538000	0.662378000	-2.130375000
C	3.752198000	-1.169144000	-3.865081000
H	4.210517000	-2.129414000	-4.099154000
H	3.488759000	-0.688742000	-4.812814000
H	4.509247000	-0.550693000	-3.368081000
C	2.503159000	-1.292309000	-2.974120000
C	-2.859953000	-2.856417000	-5.698194000

H	-2.550178000	-3.886914000	-5.897736000
H	-3.240988000	-2.795230000	-4.675312000
H	-3.687368000	-2.620871000	-6.377105000
C	-3.182618000	-7.687871000	-2.562671000
H	-2.269258000	-8.050678000	-2.082060000
H	-3.925468000	-7.522617000	-1.778427000
H	-3.556667000	-8.472385000	-3.231304000
C	2.355864000	-4.528587000	-4.394096000
C	-1.832566000	-6.712724000	-4.407683000
H	-1.617230000	-5.839046000	-5.027228000
H	-0.895389000	-7.057493000	-3.969903000
H	-2.196495000	-7.508784000	-5.066387000
C	1.556914000	-5.271190000	-0.318373000
H	1.513342000	-6.160113000	-0.954447000
H	1.818081000	-4.416500000	-0.947200000
H	2.366936000	-5.416042000	0.404995000
C	0.370108000	-3.766220000	1.262555000
H	0.543624000	-2.880376000	0.643889000
H	-0.531295000	-3.580777000	1.853114000
H	1.208201000	-3.874932000	1.959453000
C	1.691661000	-5.912442000	-4.443769000
H	1.237102000	-6.156080000	-3.481049000
H	0.922289000	-5.986224000	-5.213266000
H	2.446342000	-6.674911000	-4.664573000
C	2.972385000	-4.249603000	-5.782241000
H	2.197822000	-4.258548000	-6.554504000
H	3.456991000	-3.270411000	-5.810861000
H	3.720241000	-5.011766000	-6.032143000
Y	1.199421000	3.378631000	2.501626000
C	1.387449000	4.093893000	-1.289467000
C	0.917026000	-0.703048000	2.828658000
H	1.098508000	-1.156752000	3.808055000
H	0.038374000	-0.063837000	2.898617000
H	0.674832000	-1.497156000	2.126509000
C	3.474985000	2.266615000	3.234123000
C	2.463526000	1.231256000	3.250117000
C	1.172412000	4.613468000	0.118521000
C	1.580973000	1.541780000	4.318383000
H	0.721523000	0.950491000	4.593323000
C	2.001607000	2.706675000	4.999176000
C	3.285204000	-0.986612000	2.263390000
H	3.509064000	-1.359458000	3.267969000
H	2.967802000	-1.835395000	1.649360000
H	4.209354000	-0.600595000	1.834461000
C	1.253520000	5.312254000	-2.225146000
H	0.271972000	5.781793000	-2.110714000

H	2.018095000	6.063048000	-2.000937000
H	1.367809000	5.003725000	-3.270361000
C	0.036575000	5.629262000	1.880370000
C	4.959887000	1.935388000	1.127382000
H	4.605250000	0.931287000	0.904346000
H	4.389460000	2.645142000	0.524988000
H	6.003102000	1.988772000	0.798191000
C	2.107364000	5.294284000	0.932485000
H	3.160003000	5.390955000	0.707519000
C	1.570623000	3.054781000	6.415631000
C	1.828252000	0.557909000	0.906107000
H	1.402506000	-0.237393000	0.290278000
H	1.036704000	1.321286000	0.910780000
H	2.698744000	0.963913000	0.394812000
C	3.142841000	3.166787000	4.301343000
H	3.744904000	4.013004000	4.601008000
C	1.440624000	5.960425000	2.010657000
C	-0.086139000	4.805439000	0.728598000
H	-1.014079000	4.403356000	0.354219000
C	1.867905000	7.098028000	4.290078000
H	0.814511000	7.221486000	4.536815000
H	2.220584000	6.171359000	4.747234000
H	2.409656000	7.926334000	4.759161000
C	0.320328000	3.064627000	-1.673274000
H	0.283893000	2.219917000	-0.979786000
H	-0.672116000	3.518034000	-1.698894000
H	0.522968000	2.666613000	-2.671596000
C	-0.986807000	5.123146000	4.098931000
H	-1.046081000	4.038470000	3.918484000
H	-0.040170000	5.358193000	4.589816000
H	-1.788884000	5.338494000	4.814149000
C	2.155335000	0.051925000	2.320806000
C	2.419129000	2.153010000	7.337280000
H	2.253974000	1.096793000	7.105559000
H	3.485413000	2.363861000	7.209247000
H	2.157040000	2.322404000	8.387831000
C	4.878178000	2.297597000	2.612998000
C	-2.458481000	5.339745000	2.133979000
H	-2.658961000	5.808300000	1.166956000
H	-2.410894000	4.257776000	1.988990000
H	-3.309863000	5.544476000	2.790856000
C	-1.435383000	7.382570000	3.041652000
H	-0.636511000	7.873468000	3.595104000
H	-1.567152000	7.913356000	2.093946000
H	-2.356569000	7.498774000	3.622796000
C	-1.177116000	5.892908000	2.781010000

C	2.778714000	3.490560000	-1.470919000
H	3.568076000	4.190523000	-1.178605000
H	2.886698000	2.579090000	-0.881270000
H	2.941389000	3.224800000	-2.519653000
C	5.748085000	1.327848000	3.437123000
H	5.760059000	1.630281000	4.488871000
H	5.369054000	0.305804000	3.389411000
H	6.779493000	1.330543000	3.066271000
C	2.153873000	7.074159000	2.787371000
C	5.506319000	3.693572000	2.724425000
H	4.924263000	4.431070000	2.166837000
H	5.605774000	4.033678000	3.758394000
H	6.512984000	3.671383000	2.295400000
C	1.840204000	4.511357000	6.794061000
H	2.875493000	4.802262000	6.590099000
H	1.177844000	5.196291000	6.261622000
H	1.665049000	4.654435000	7.865317000
C	0.089512000	2.739726000	6.655827000
H	-0.560076000	3.334808000	6.009316000
H	-0.133940000	1.683417000	6.481589000
H	-0.176326000	2.962688000	7.694424000
C	3.679707000	6.944133000	2.668602000
H	4.031523000	6.001435000	3.092584000
H	4.027638000	7.017306000	1.635084000
H	4.154184000	7.757572000	3.226586000
C	1.766968000	8.414228000	2.132164000
H	2.063017000	8.414493000	1.078583000
H	0.694198000	8.600349000	2.173655000
H	2.279371000	9.243633000	2.633098000

TS3’
E= -3485.76529753 Ha
Sum of electronic and thermal Enthalpies= -3483.916418 Ha
Sum of electronic and thermal Free Energies= -3484.143987 Ha
Imag_freq=-127.8 cm-1

Mo	-2.257201000	-0.141254000	1.318370000
O	-4.411371000	1.884596000	0.507391000
O	-3.391191000	1.373882000	-2.030197000
O	-2.048029000	-1.152833000	-2.358484000
O	-2.295298000	-2.219914000	3.730383000
O	-0.505591000	1.704812000	3.029981000
O	-0.186645000	-1.615968000	-0.496323000
C	-3.461310000	1.170418000	0.409848000
C	-2.259132000	-1.535270000	2.800995000
C	-2.948146000	0.679844000	-1.142832000
C	-2.070667000	-0.479810000	-1.269794000

C	-1.240715000	0.976226000	2.390303000
C	-1.150835000	-0.837546000	-0.179506000
Y	-0.747230000	-3.000676000	-2.324637000
C	-1.163774000	-1.441904000	-5.649536000
C	-4.728328000	-2.602881000	-0.089812000
H	-4.620608000	-3.212811000	0.812449000
H	-3.976383000	-1.809088000	-0.058723000
H	-5.711599000	-2.123486000	-0.060249000
C	-2.645684000	-5.056622000	-2.373040000
C	-3.218086000	-4.097497000	-1.456889000
C	-0.132810000	-2.075820000	-4.735402000
C	-2.311771000	-3.989885000	-0.365364000
H	-2.446734000	-3.339301000	0.486157000
C	-1.223718000	-4.873184000	-0.527224000
C	-5.682081000	-4.537033000	-1.232663000
H	-5.441502000	-5.197056000	-0.392914000
H	-6.657712000	-4.077294000	-1.038042000
H	-5.783850000	-5.154248000	-2.123938000
C	-0.618269000	-1.528087000	-7.086574000
H	0.346153000	-1.015939000	-7.163396000
H	-0.471740000	-2.569458000	-7.391171000
H	-1.314960000	-1.059585000	-7.791443000
C	1.717034000	-2.318314000	-3.340121000
C	-4.299945000	-5.218345000	-4.390365000
H	-5.206704000	-4.963715000	-3.845842000
H	-3.912970000	-4.310532000	-4.854876000
H	-4.590779000	-5.904021000	-5.193988000
C	0.275508000	-3.427861000	-4.742563000
H	-0.137660000	-4.189156000	-5.388275000
C	-0.213839000	-5.261130000	0.534355000
C	-4.941481000	-2.500596000	-2.540827000
H	-5.958945000	-2.111172000	-2.421204000
H	-4.253335000	-1.653864000	-2.566370000
H	-4.889388000	-2.996461000	-3.507163000
C	-1.420190000	-5.500021000	-1.778356000
H	-0.806945000	-6.296486000	-2.174529000
C	1.434084000	-3.609074000	-3.920374000
C	0.734066000	-1.416813000	-3.843500000
H	0.651605000	-0.374494000	-3.577300000
C	3.288249000	-5.251898000	-3.055679000
H	4.052373000	-4.496441000	-2.882604000
H	2.786718000	-5.462228000	-2.110455000
H	3.807954000	-6.166853000	-3.360548000
C	-1.399438000	0.028850000	-5.293179000
H	-1.785516000	0.134363000	-4.277214000
H	-0.473645000	0.607341000	-5.384643000

H	-2.130560000	0.464405000	-5.982607000
C	3.077641000	-2.499547000	-1.172122000
H	2.182849000	-2.371268000	-0.554909000
H	3.270712000	-3.564007000	-1.277874000
H	3.925695000	-2.062901000	-0.632926000
C	-4.606432000	-3.442513000	-1.374230000
C	-0.720377000	-6.546859000	1.212231000
H	-1.707713000	-6.382743000	1.654639000
H	-0.808129000	-7.360966000	0.485792000
H	-0.034124000	-6.864584000	2.006455000
C	-3.239093000	-5.895082000	-3.518046000
C	2.677599000	-0.305313000	-2.191502000
H	2.650103000	0.310911000	-3.094124000
H	1.748545000	-0.154699000	-1.639506000
H	3.506801000	0.053209000	-1.572550000
C	4.191305000	-1.847393000	-3.350676000
H	4.497692000	-2.863424000	-3.597046000
H	4.057376000	-1.303878000	-4.291381000
H	5.013897000	-1.377196000	-2.798847000
C	2.896876000	-1.788238000	-2.519771000
C	-2.490643000	-2.203232000	-5.574098000
H	-2.344760000	-3.261430000	-5.812139000
H	-2.930645000	-2.123541000	-4.575896000
H	-3.216428000	-1.796753000	-6.287438000
C	-3.846925000	-7.162448000	-2.880102000
H	-3.075996000	-7.727837000	-2.347847000
H	-4.630931000	-6.915062000	-2.161805000
H	-4.279031000	-7.810297000	-3.652065000
C	2.287748000	-4.867533000	-4.149579000
C	-2.139100000	-6.355757000	-4.485919000
H	-1.655552000	-5.500156000	-4.961268000
H	-1.368563000	-6.952790000	-3.995692000
H	-2.579322000	-6.977596000	-5.272728000
C	1.150600000	-5.535133000	-0.096674000
H	1.089602000	-6.322405000	-0.853992000
H	1.542058000	-4.633165000	-0.573689000
H	1.877468000	-5.853052000	0.659276000
C	-0.071515000	-4.161521000	1.591265000
H	0.190384000	-3.202838000	1.133565000
H	-1.004614000	-4.029476000	2.144725000
H	0.702554000	-4.434710000	2.316942000
C	1.395627000	-6.102775000	-4.341316000
H	0.801033000	-6.289440000	-3.444289000
H	0.712672000	-6.005821000	-5.186694000
H	2.019426000	-6.983668000	-4.526560000
C	3.063666000	-4.636443000	-5.463838000

H	2.370138000	-4.465849000	-6.292370000
H	3.715595000	-3.762439000	-5.393406000
H	3.682503000	-5.509315000	-5.703943000
Y	1.286228000	3.041499000	2.615476000
C	0.960448000	3.435844000	-1.245234000
C	1.756568000	-1.035222000	3.179655000
H	2.153174000	-1.380405000	4.139440000
H	0.765384000	-0.606713000	3.343290000
H	1.622626000	-1.905773000	2.532875000
C	3.760701000	2.377190000	3.195875000
C	2.943459000	1.192846000	3.370274000
C	0.803625000	4.003378000	0.153985000
C	2.120431000	1.427467000	4.501147000
H	1.391712000	0.729456000	4.883402000
C	2.382877000	2.698616000	5.058335000
C	3.997438000	-0.836416000	2.200847000
H	4.485182000	-1.125558000	3.137001000
H	3.745437000	-1.749727000	1.654577000
H	4.719183000	-0.285338000	1.599875000
C	0.436359000	4.527887000	-2.201366000
H	-0.603451000	4.777480000	-1.971673000
H	1.034542000	5.440710000	-2.115911000
H	0.483567000	4.177842000	-3.238556000
C	-0.360736000	4.975717000	1.925881000
C	4.907029000	2.327074000	0.886589000
H	4.626852000	1.299807000	0.658441000
H	4.158943000	2.990381000	0.445345000
H	5.859415000	2.522121000	0.382213000
C	1.655593000	4.918844000	0.812780000
H	2.645746000	5.188267000	0.472143000
C	1.967629000	3.126432000	6.454473000
C	2.028788000	0.399709000	1.196929000
H	1.813828000	-0.444022000	0.537841000
H	1.024458000	0.804955000	1.396254000
H	2.615850000	1.125051000	0.629788000
C	3.371550000	3.285447000	4.232633000
H	3.841315000	4.239977000	4.422296000
C	0.962988000	5.562300000	1.893224000
C	-0.411183000	4.017123000	0.876395000
H	-1.273083000	3.410266000	0.638461000
C	1.198564000	7.154018000	3.937793000
H	0.132982000	7.224190000	4.146037000
H	1.626484000	6.381491000	4.580040000
H	1.647926000	8.109084000	4.229961000
C	0.105830000	2.181154000	-1.431259000
H	0.356121000	1.401883000	-0.707941000

H	-0.956221000	2.409692000	-1.328218000
H	0.245666000	1.768176000	-2.434682000
C	-1.259209000	4.812880000	4.277610000
H	-1.096866000	3.730128000	4.344638000
H	-0.379225000	5.325574000	4.664463000
H	-2.099082000	5.040844000	4.943517000
C	2.717016000	-0.047060000	2.495612000
C	3.027736000	2.548804000	7.414145000
H	3.080982000	1.459819000	7.322510000
H	4.018319000	2.955331000	7.187555000
H	2.781777000	2.796759000	8.452991000
C	5.040865000	2.612546000	2.385123000
C	-2.757562000	4.323153000	2.380638000
H	-3.080704000	4.554876000	1.362469000
H	-2.522420000	3.258302000	2.429494000
H	-3.609718000	4.498558000	3.044464000
C	-2.085538000	6.656160000	2.741671000
H	-1.376435000	7.389771000	3.122337000
H	-2.312542000	6.914153000	1.702715000
H	-3.008355000	6.757813000	3.322811000
C	-1.580537000	5.207163000	2.827269000
C	2.413822000	3.138036000	-1.609437000
H	3.059993000	4.007688000	-1.451736000
H	2.811965000	2.300732000	-1.034902000
H	2.482228000	2.865169000	-2.666843000
C	6.158865000	1.756693000	3.009637000
H	6.292699000	2.025813000	4.062007000
H	5.937187000	0.690884000	2.965330000
H	7.106777000	1.931720000	2.487955000
C	1.524620000	6.870531000	2.469022000
C	5.490763000	4.077408000	2.481393000
H	4.732577000	4.751269000	2.077179000
H	5.718658000	4.379034000	3.507339000
H	6.403928000	4.212548000	1.893519000
C	1.936687000	4.645812000	6.619574000
H	2.876074000	5.111467000	6.305666000
H	1.121564000	5.087788000	6.043226000
H	1.774946000	4.907023000	7.670464000
C	0.596663000	2.553163000	6.831954000
H	-0.185685000	2.900653000	6.151608000
H	0.597876000	1.459570000	6.816229000
H	0.325140000	2.866459000	7.845382000
C	3.058167000	6.902226000	2.387294000
H	3.500137000	6.109310000	2.995291000
H	3.432169000	6.810810000	1.364904000
H	3.420489000	7.859280000	2.775805000

C	0.992235000	8.011733000	1.578959000
H	1.313924000	7.863745000	0.543506000
H	-0.097661000	8.056039000	1.583899000
H	1.377781000	8.977765000	1.924746000

Int5'

E= -3485.76641329 Ha

Sum of electronic and thermal Enthalpies= -3483.916284 Ha

Sum of electronic and thermal Free Energies= -3484.145912 Ha

Mo	-2.178617000	0.105164000	1.110276000
O	-4.253752000	2.107230000	0.331852000
O	-3.586047000	1.232218000	-2.342935000
O	-2.049704000	-1.074290000	-2.470984000
O	-3.096875000	-2.032206000	3.277976000
O	-0.431627000	1.774950000	3.025409000
O	-0.215363000	-1.560317000	-0.654101000
C	-3.370745000	1.329577000	0.058740000
C	-2.775777000	-1.269397000	2.474257000
C	-3.055267000	0.750636000	-1.356575000
C	-2.110426000	-0.388872000	-1.398209000
C	-1.149291000	1.127949000	2.289459000
C	-1.138884000	-0.754982000	-0.313724000
Y	-0.774720000	-2.940633000	-2.468782000
C	-1.154909000	-1.477072000	-5.834950000
C	-4.826070000	-2.469927000	-0.457850000
H	-4.757262000	-3.024332000	0.483341000
H	-4.086949000	-1.667877000	-0.438661000
H	-5.814709000	-2.002568000	-0.500950000
C	-2.622767000	-5.022885000	-2.504665000
C	-3.247472000	-4.030356000	-1.661762000
C	-0.128641000	-2.064466000	-4.886056000
C	-2.395985000	-3.862070000	-0.533587000
H	-2.578839000	-3.175290000	0.279442000
C	-1.291731000	-4.736569000	-0.603910000
C	-5.715428000	-4.477984000	-1.530082000
H	-5.520375000	-5.077306000	-0.634637000
H	-6.703515000	-4.016163000	-1.423096000
H	-5.760795000	-5.155930000	-2.380942000
C	-0.554310000	-1.526691000	-7.251534000
H	0.378652000	-0.955590000	-7.293792000
H	-0.332392000	-2.556623000	-7.549500000
H	-1.252851000	-1.100567000	-7.981051000
C	1.697727000	-2.225326000	-3.449375000
C	-4.195221000	-5.326316000	-4.577483000
H	-5.125525000	-5.048685000	-4.086448000
H	-3.801724000	-4.446513000	-5.088224000

H	-4.445489000	-6.068832000	-5.343162000
C	0.310024000	-3.406886000	-4.847568000
H	-0.075281000	-4.196670000	-5.476093000
C	-0.328218000	-5.067501000	0.519099000
C	-4.920054000	-2.510184000	-2.913566000
H	-5.946714000	-2.128924000	-2.871684000
H	-4.244880000	-1.651989000	-2.939620000
H	-4.809503000	-3.055832000	-3.847557000
C	-1.419336000	-5.420239000	-1.834040000
H	-0.775617000	-6.224537000	-2.161182000
C	1.452844000	-3.538908000	-3.994895000
C	0.706037000	-1.361083000	-3.997236000
H	0.596785000	-0.313756000	-3.762360000
C	3.299461000	-5.115449000	-3.011211000
H	4.058780000	-4.350676000	-2.858175000
H	2.768189000	-5.272462000	-2.071055000
H	3.827527000	-6.045315000	-3.249319000
C	-1.482323000	-0.022834000	-5.481357000
H	-1.915549000	0.060393000	-4.481964000
H	-0.586103000	0.604981000	-5.532630000
H	-2.205289000	0.377881000	-6.199876000
C	3.033898000	-2.302414000	-1.257491000
H	2.131081000	-2.166857000	-0.654768000
H	3.243889000	-3.366452000	-1.322443000
H	3.870815000	-1.832916000	-0.729226000
C	-4.641483000	-3.383528000	-1.681088000
C	-0.818293000	-6.370115000	1.177471000
H	-1.836865000	-6.246475000	1.557866000
H	-0.826263000	-7.192336000	0.455013000
H	-0.168339000	-6.651984000	2.014623000
C	-3.159004000	-5.930991000	-3.624672000
C	2.602736000	-0.153176000	-2.357024000
H	2.571411000	0.430687000	-3.280985000
H	1.667113000	0.000414000	-1.816242000
H	3.420652000	0.242548000	-1.746000000
C	4.160743000	-1.706505000	-3.442019000
H	4.493133000	-2.725338000	-3.638402000
H	4.028347000	-1.207921000	-4.407465000
H	4.964675000	-1.194198000	-2.900216000
C	2.854135000	-1.642892000	-2.630673000
C	-2.439998000	-2.307837000	-5.807892000
H	-2.237192000	-3.349415000	-6.075739000
H	-2.897403000	-2.283822000	-4.814722000
H	-3.176894000	-1.916002000	-6.517740000
C	-3.773077000	-7.170415000	-2.938908000
H	-3.016818000	-7.689804000	-2.342587000

H	-4.590532000	-6.896827000	-2.268990000
H	-4.161648000	-7.869740000	-3.688798000
C	2.332301000	-4.790565000	-4.152642000
C	-2.017198000	-6.427163000	-4.523846000
H	-1.534601000	-5.592372000	-5.036230000
H	-1.253788000	-6.976874000	-3.972081000
H	-2.418746000	-7.104778000	-5.284972000
C	1.084225000	-5.287071000	-0.025351000
H	1.096996000	-6.067826000	-0.791914000
H	1.478872000	-4.367600000	-0.465882000
H	1.770194000	-5.591521000	0.773323000
C	-0.298297000	-3.957248000	1.574546000
H	-0.022181000	-2.997275000	1.129594000
H	-1.275191000	-3.845212000	2.053244000
H	0.424535000	-4.205351000	2.359786000
C	1.467063000	-6.047308000	-4.324986000
H	0.858520000	-6.221136000	-3.434649000
H	0.799651000	-5.988163000	-5.185856000
H	2.110975000	-6.920816000	-4.472769000
C	3.145336000	-4.598529000	-5.450331000
H	2.475168000	-4.479498000	-6.306763000
H	3.776430000	-3.708313000	-5.398489000
H	3.789254000	-5.467161000	-5.633118000
Y	1.345740000	3.152044000	2.566439000
C	0.913145000	3.604895000	-1.278973000
C	1.822950000	-0.922918000	3.039567000
H	2.226292000	-1.284571000	3.990467000
H	0.835343000	-0.491528000	3.218454000
H	1.678379000	-1.784119000	2.382317000
C	3.837171000	2.485179000	3.055923000
C	3.026906000	1.297682000	3.240659000
C	0.794623000	4.148278000	0.133915000
C	2.239402000	1.514769000	4.399609000
H	1.523481000	0.809811000	4.793594000
C	2.518532000	2.778460000	4.966340000
C	4.063226000	-0.725294000	2.060620000
H	4.553820000	-1.009184000	2.996928000
H	3.809838000	-1.641818000	1.520959000
H	4.783723000	-0.177135000	1.455562000
C	0.366915000	4.715289000	-2.200933000
H	-0.665645000	4.963464000	-1.939109000
H	0.969837000	5.624879000	-2.114750000
H	0.385574000	4.384312000	-3.245208000
C	-0.321946000	5.090415000	1.952483000
C	4.954092000	2.429664000	0.723861000
H	4.687890000	1.396542000	0.506376000

H	4.195494000	3.078770000	0.279972000
H	5.900627000	2.632879000	0.211884000
C	1.663265000	5.053870000	0.784207000
H	2.643185000	5.331159000	0.421039000
C	2.147175000	3.185235000	6.381004000
C	2.091652000	0.529157000	1.068608000
H	1.877093000	-0.305011000	0.397995000
H	1.087417000	0.932147000	1.275749000
H	2.674107000	1.264063000	0.510403000
C	3.480019000	3.377907000	4.117935000
H	3.955084000	4.329893000	4.306479000
C	0.999665000	5.679499000	1.893501000
C	-0.399814000	4.148007000	0.890220000
H	-1.268438000	3.545368000	0.666257000
C	1.290045000	7.240444000	3.954419000
H	0.230586000	7.303212000	4.194027000
H	1.738895000	6.460339000	4.572472000
H	1.744047000	8.192825000	4.248054000
C	0.051793000	2.355056000	-1.462982000
H	0.330616000	1.559287000	-0.768712000
H	-1.005814000	2.575918000	-1.306290000
H	0.151005000	1.965150000	-2.480564000
C	-1.155469000	4.887159000	4.322885000
H	-0.990438000	3.803446000	4.367359000
H	-0.265308000	5.394382000	4.693427000
H	-1.976259000	5.101922000	5.016264000
C	2.784889000	0.066826000	2.359363000
C	3.236654000	2.592739000	7.297838000
H	3.287166000	1.505378000	7.187250000
H	4.219717000	3.003420000	7.047361000
H	3.022919000	2.823947000	8.347592000
C	5.099369000	2.730948000	2.218609000
C	-2.706413000	4.428660000	2.458808000
H	-3.058020000	4.676705000	1.454354000
H	-2.471589000	3.362545000	2.479731000
H	-3.539689000	4.591309000	3.149349000
C	-2.024425000	6.755345000	2.840250000
H	-1.304915000	7.482996000	3.212636000
H	-2.279389000	7.029028000	1.811900000
H	-2.931239000	6.847875000	3.447453000
C	-1.517672000	5.305456000	2.888986000
C	2.355196000	3.308065000	-1.686956000
H	3.009161000	4.172475000	-1.532653000
H	2.764070000	2.459730000	-1.136508000
H	2.394113000	3.051152000	-2.749828000
C	6.235721000	1.891933000	2.833794000

H	6.384022000	2.171674000	3.881428000
H	6.022310000	0.823637000	2.802620000
H	7.173497000	2.070217000	2.295225000
C	1.576511000	6.979082000	2.473547000
C	5.532925000	4.202122000	2.294476000
H	4.762668000	4.863370000	1.891774000
H	5.769552000	4.518301000	3.313886000
H	6.437802000	4.341306000	1.694871000
C	2.121964000	4.701821000	6.570601000
H	3.051226000	5.172609000	6.234870000
H	1.289205000	5.152859000	6.027372000
H	1.993376000	4.946361000	7.630001000
C	0.788351000	2.606216000	6.792342000
H	-0.015059000	2.964475000	6.142746000
H	0.788340000	1.513035000	6.759711000
H	0.549167000	2.903862000	7.818472000
C	3.107453000	7.010042000	2.351530000
H	3.563213000	6.200517000	2.926588000
H	3.453570000	6.943729000	1.317369000
H	3.482812000	7.956301000	2.753747000
C	1.021808000	8.133346000	1.614724000
H	1.315679000	8.000533000	0.569002000
H	-0.067485000	8.178489000	1.649223000
H	1.417615000	9.093795000	1.964312000

References

(1) Mondal, A.; Price, C. G. T.; Tang, J.; Layfield, R. A. Targeted Synthesis of End-On Dinitrogen-Bridged Lanthanide Metallocenes and Their Reactivity as Divalent Synthons. *J. Am. Chem. Soc.* **2023**, *145*, 20121-20131.
(2) Mondal, A.; Price, C. G. T.; Steiner, A.; Tang, J.; Layfield, R. A. Reduction of Hexaazatrinaphthylenes by Masked Divalent Lanthanide Dinitrogen Reagents. *Inorg. Chem.* **2025**, *64*, 13309-13317.
(3) Dolomanov, O. V.; Bourhis, L. J.; Gildea, R. J.; Howard, J. A. K.; Puschmann, H. OLEX2: a complete structure solution, refinement and analysis program. *J. Appl. Crystallogr.* **2009**, *42* (2), 339-341.
(4) Bourhis, L. J.; Dolomanov, O. V.; Gildea, R. J.; Howard, J. A. K.; Puschmann, H. The anatomy of a comprehensive constrained, restrained refinement program for the modern computing environment – Olex2 dissected. *Acta Crystallogr. A* **2015**, *71* (1), 59-75.
(5) Sheldrick, G. M. Crystal structure refinement with SHELXL. *Acta Crystallographica Section C* **2015**, *71*, 3-8.
(6) Bain, G. A.; Berry, J. F. Diamagnetic Corrections and Pascal's Constants. *J. Chem. Ed.* **2008**, *85*, 532-532.
(7) Chilton, N. F.; Anderson, R. P.; Turner, L. D.; Soncini, A.; Murray, K. S. PHI: A powerful new program for the analysis of anisotropic monomeric and exchange-coupled polynuclear d- and f-block complexes. *J. Comput. Chem.* **2013**, *34*, 1164-1175.
(8) Neese, F.; Wennmohs, F.; Becker, U.; Riplinger, C. The ORCA quantum chemistry program package. *J. Chem. Phys.* **2020**, *152*, 224108-224108.
(9) Perdew, J. P.; Burke, K.; Ernzerhof, M. Generalized Gradient Approximation Made Simple. *Phys. Rev. Lett.* **1996**, *77*, 3865-3868.
(10) Perdew, J. P.; Burke, K.; Ernzerhof, M. Generalized Gradient Approximation Made Simple. *Phys. Rev. Lett.* **1997**, *78* (7), 1396-1396.
(11) Tao, J.; Perdew, J. P.; Staroverov, V. N.; Scuseria, G. E. Climbing the Density Functional Ladder: Nonempirical Meta--Generalized Gradient Approximation Designed for Molecules and Solids. *Phys. Rev. Lett.* **2003**, *91*, 146401-146401.
(12) Neese, F.; Olbrich, G. Efficient use of the resolution of the identity approximation in time-dependent density functional calculations with hybrid density functionals. *Chem. Phys. Lett.* **2002**, *362*, 170-178.
(13) Aravena, D.; Neese, F.; Pantazis, D. A. Improved Segmented All-Electron Relativistically Contracted Basis Sets for the Lanthanides. *J. Chem. Theor. Comput.* **2016**, *12*, 1148-1156.
(14) Chmela, J.; Harding, M. E. Optimized auxiliary basis sets for density fitted post-Hartree–Fock calculations of lanthanide containing molecules. *Mol. Phys.* **2018**, *116*, 1523-1538.
(15) Rolfes, J. D.; Neese, F.; Pantazis, D. A. All-electron scalar relativistic basis sets for the elements Rb–Xe. *J, Comput, Chem,* **2020**, *41*, 1842-1849.
(16) Izsák, R.; Neese, F. An overlap fitted chain of spheres exchange method. *J. Chem. Phys.* **2011**, *135*, 144105-144105.
(17) Neese, F.; Wennmohs, F.; Hansen, A.; Becker, U. Efficient, approximate and parallel Hartree–Fock and hybrid DFT calculations. A ‘chain-of-spheres’ algorithm for the Hartree–Fock exchange. *Chem. Phys.* **2009**, *356*, 98-109.
(18) Takano, Y.; Houk, K. N. Benchmarking the Conductor-like Polarizable Continuum Model (CPCM) for Aqueous Solvation Free Energies of Neutral and Ionic Organic Molecules. *J. Chem. Theory Comput.* **2005**, *1*, 70-77.

(19) Garcia-Ratés, M.; Neese, F. Effect of the Solute Cavity on the Solvation Energy and its Derivatives within the Framework of the Gaussian Charge Scheme. *J. Comput. Chem.* **2020**, *41*, 922-939.
(20) Dolg, M.; Stoll, H.; Savin, A.; Preuss, H. Energy-adjusted pseudopotentials for the rare earth elements. *Theor. Chim. Acta* **1989**, *75*, 173-194.
(21) Dolg, M.; Stoll, H.; Preuss, H. A combination of quasirelativistic pseudopotential and ligand field calculations for lanthanoid compounds. *Theor. Chem. Acc.* **1993**, *85*, 441-450.
(22) Hariharan, P. C.; Pople, J. A. The influence of polarization functions on molecular orbital hydrogenation energies. *Theor. Chem. Acc.* **1973**, *28*, 213-222.
(23) Grimme, S.; Ehrlich, S.; Goerigk, L. Effect of the damping function in dispersion corrected density functional theory. *J. Comput. Chem.* **2011**, *32*, 1456-1465.
(24) Hehre, W. J.; Ditchfield, R.; Pople, J. A. Self—Consistent Molecular Orbital Methods. XII. Further Extensions of Gaussian—Type Basis Sets for Use in Molecular Orbital Studies of Organic Molecules. *J. Chem. Phys.* **1972**, *56*, 2257-2261.
(25) Frisch, M. J.; Trucks, G. W.; Schlegel, H. B.; Scuseria, G. E.; Robb, M. A.; Cheeseman, J. R.; Scalmani, G.; Barone, V.; Mennucci, B.; Petersson, G. A.; et al. Gaussian09 Revision E.01. **2009**.
